# The effect of inflammatory proteins on COVID-19 is mediated by blood metabolites: A Mendelian randomization study

**DOI:** 10.1097/MD.0000000000041852

**Published:** 2025-03-14

**Authors:** Yuling Chen, Chang Chen

**Affiliations:** a Department of Clinical Laboratory, Beijing Anzhen Nanchong Hospital of Capital Medical University & Nanchong Central Hospital, The Second Clinical Medical College of North Sichuan Medical College, Nanchong, Sichuan, China; b Medical Department, Nanchong Guoning Mental Health Hospital, Nanchong, Sichuan, China.

**Keywords:** blood metabolites, COVID-19, IL-10, IL-18, inflammatory proteins, PD-L1

## Abstract

Several studies have suggested that inflammatory proteins may be associated with Coronavirus disease 2019 (COVID-19). However, the specific causal relationship between the 2 and whether blood metabolites act as mediators remains unclear. Therefore, the purpose of the present study is to investigate the causal relationship between inflammatory proteins and COVID-19 and to identify and quantify the role of blood metabolites as potential mediators. Two-sample Mendelian randomization (MR) and 2-step mediated MR analyses were used to investigate the causal relationships between 91 inflammatory proteins, 486 blood metabolites and COVID-19. A random-effects inverse variance weighted (IVW) approach was used as the primary analytical method, supplemented by weighted medians, MR-Egger and MR multivariate residual sums, and outliers to test MR hypotheses. Our results showed that 2 inflammatory proteins (interleukin-10 and interleukin-18) were positively associated with COVID-19 risk, while 1 inflammatory protein (PD-L1) was negatively associated. Further validation was performed using sensitivity analysis. The results of mediated MR showed that Betaine was a mediator of PD-L1 to COVID-19 with a mediation ratio of 15.92%. Our study suggests a genetic causality between specific inflammatory proteins and COVID-19, highlights the potential mediating role of the blood metabolite betaine, and contributes to a deeper understanding of the mechanism of action of severe COVID-19.

## 1. Introduction

Coronavirus disease 2019 (COVID-19), caused by Severe Acute Respiratory Syndrome Coronavirus type 2 (SARS-CoV-2), has dealt a far-reaching blow to healthcare systems, societies, and economic development worldwide.^[1]^ In the initial phase of the global outbreak of the novel COVID-19, characterized by a paucity of knowledge regarding SARS-CoV-2, there has been a pervasive atmosphere of public alarm and distress.^[2]^

Its most common symptoms are dry cough, fever, muscle pain, and in severe cases, even accumulation of cardiovascular, neurological, gastrointestinal and skin systems.^[3]^ SARS-CoV-2 is a simplex RNA virus belonging to the family of coronaviruses in the genus β-coronavirus. Viral virulence and function are determined primarily by 4 strategic proteins in the genome: nucleocapsid protein (N), membrane protein (M), spiculin (S), and envelope protein (E).^[4]^ Protein S mediates SARS-CoV-2 infection of reservoir cells by engaging with angiotensin-converting enzyme 2, which blocks the renin-angiotensin-aldosterone pathway, leading to elevated levels of angiotensin II and angiotensin-converting enzyme 2. This may also be the main reason for the elevated pro-inflammatory cytokines and acute respiratory distress seen in COVID-19 patients.^[5]^

COVID-19 produces a severe inflammatory process that leads to prolonged symptoms after the disease subsides.^[6]^ One of the key causative factors is immune dysregulation. It has been found that the severity of COVID-19 is related to the levels of pro-inflammatory cytokines and cellular immune features.^[7]^ It was found that after SARS-CoV-2 infection, patients had increased level of various cytokines in their plasma, including IL-1β, IL-1 RA, IL 4, IL-7, IL-8, interleukin-10 (IL-10), MIP-1α, MCP-1, IFN-γ, G-CSF, and TNF-α. These elevated levels correlate with the severity of the disease, and may lead to inflammatory responses and immune system suppression of function.^[8]^ Therefore, control of inflammatory protein factors is crucial in COVID-19. In addition, the potential pathways of inflammatory proteins and COVID-19 have not been clearly studied. Previous studies have found that many blood metabolites are altered in COVID-19.^[9]^ Thus, blood metabolites may be potential mediators between inflammatory proteins and COVID-19.

Mendelian randomization (MR) is a method that uses single nucleotide polymorphisms (SNPs) as genetically instrumented variables to assess the causal effect of exposure on outcomes. Because genetic variants are randomly assigned, MR studies are less susceptible to confounding than traditional observational studies.^[10]^ Therefore, we aimed to determine whether inflammatory proteins are causally related to COVID-19 and assess the extent to which blood metabolites mediate the effects of inflammatory proteins on COVID-19.

## 2. Materials and methods

### 2.1. Study design

**Figure 1.**
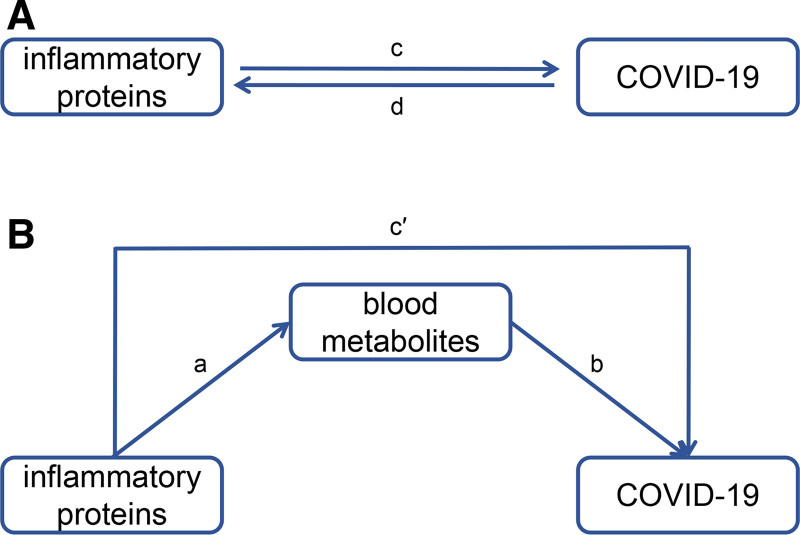
(A) The total effect between inflammatory proteins and COVID-19: c is the total effect using inflammatory proteins as the exposure and COVID-19 as the outcome; d is the total effect using COVID-19 as the exposure and inflammatory proteins as the outcome. (B) Analysis of the mediation of blood metabolites in the pathway from inflammatory proteins to COVID-19: a is the causal effect of inflammatory proteins on blood metabolites; b is the causal effect of blood metabolites on COVID-19; c′ is the direct effect of inflammatory proteins on COVID-19. COVID-19 = Coronavirus disease 2019.

The data used within our analyses were obtained form released publically available databases; as such, no additional ethical approvals were required for this study. In this study, we first explored the causal relationship between 91 inflammatory proteins and COVID-19 by 2-sample MR (Fig. 1A). Subsequently, we used mediated MR (2-step MR) to investigate in depth the etiological relationship between specific inflammatory proteins mediated by blood metabolite-related factors and COVID-19 (Fig. 1B). Two-step MR were conducted to assess the potential role of intermediate variables in the etiology of disease. The objective was to determine whether inflammatory factors contribute to the development of disease through intermediate variables. In the first step, the MR results between inflammatory factors and intermediate variables were calculated. Subsequently, the MR results between intermediate variables and SARS-CoV-2 infection were calculated. Finally, the direct and indirect effects between inflammatory factors and SARS-CoV-2 infection were integrated and calculated.

### 2.2. Data sources

The data used in our study are publicly available and the subjects are of European ancestry. Inflammatory protein data were obtained from the EBI GWAS Catalog (accession numbers GCST90274758 to GCST90274848) for 14,824 participants in a genome-wide protein quantitative trait loci study of 91 inflammation-related plasma proteins. Data on blood metabolites were obtained from the metabolomics GWAS database (https://metabolomics.helmholtz-muenchen.de/gwas/), identifying 486 blood metabolites associated with human genetic variants. Data for COVID-19 were obtained from the FennGenn Alliance’s GWAS Summary Data Source (https://www.finngen.fi/en)^[11]^ and included 2856 cases and 405,232 controls.

### 2.3. Genetic selection

We used the “TwoSampleMR” software package to screen SNPs.^[11]^ Hoping to obtain a moderate number of SNPs associated with inflammatory proteins and blood metabolites, we relaxed the significance threshold *P* < 1e^−5^ to screen SNPs.^[12]^ These SNPs were then clustered based on linkage disequilibrium (kb = 10,000 kb and *r*^2^ = 0.001). To determine the presence of weak instrumental variable bias, we calculated the *F* statistic to quantify the strength of the instrumental variable, where an *F* statistic >10 means that the likelihood of weak instrumental variable bias as low.^[13]^

### 2.4. MR analysis

MR analysis combined the “mr” feature with 5 methods, including inverse variance weighted (IVW), MR Egger, weighted median, simple mode, and weighted mode. The present study focuses on the findings of the IVW method, which is the most powerful method in MR analysis for detecting causality.^[14]^ In order to guarantee the statistical significance of the results, it was necessary for the *P* value to be <.05. The odds ratio (OR) was then computed, larger than 1 as a risk factor and <1 as a protectiveness factor. Only results that were consistent across the 5 methods of analysis were deemed to be meaningful. Outcomes were represented in scatterplots, forest plots and funnel plots. False discovery rate (FDR) correction was performed in each MR and the FDR threshold was set to *q* < 0.1. Correlation was deemed suggestive when *P* < .05 and *q* ≥ 0.1.^[15]^

### 2.5. Sensitivity analysis

To determine the robustness of the analyses, sensitivity analyses were carried out using the heterogeneity test, the horizontal multivariate validity check, and the leave-one-out sensitivity test (LOO) method. In the heterogeneity test, a *P* value larger than .05 means no heterogeneity exists. In the horizontal multivariate validity test, a *P* value larger than .05 means no horizontal multivariate validity exists. LOO was used to see if there were outliers in the effects of each SNP.

### 2.6. Intermediary effect

Using 2-step MR for mediation analysis, the total effect was decomposed into indirect and direct effects.^[16]^ The total effect of inflammatory proteins on COVID-19 was decomposed into the direct effect of inflammatory proteins on COVID-19 (a × b), and the indirect effect mediated by inflammatory factors through mediators (c′ = c − a × b). The percentage mediated by the mediator effect was calculated by dividing the indirect effect by the total effect.

## 3. Results

### 3.1. MR results of inflammatory proteins and COVID-19

**Figure 2.**
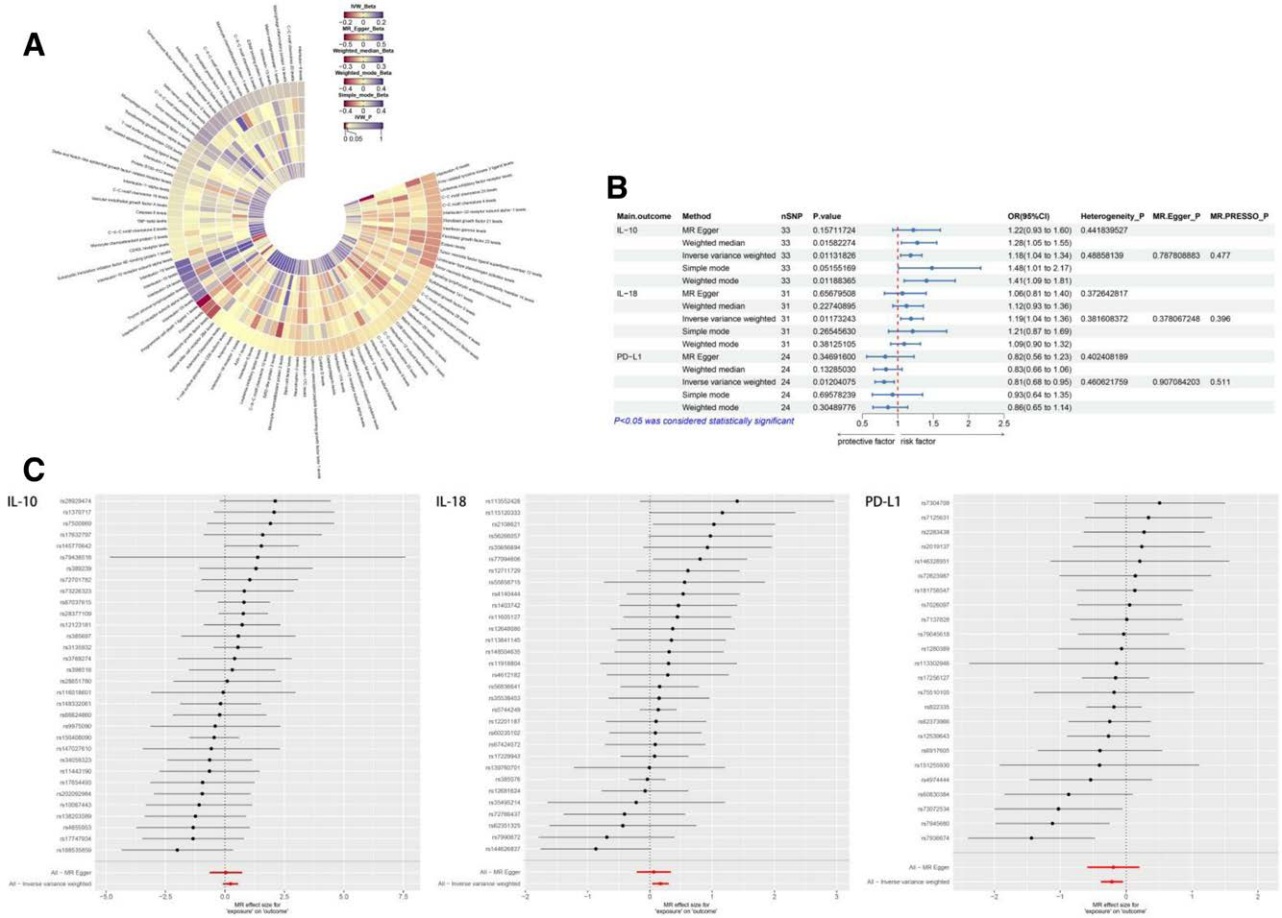
(A) Circular heat map of the causal relationship between 91 inflammatory proteins and COVID-19. (B) MR results of 3 suggestive-related inflammatory proteins causally associated with COVID-19. (C) Forest plot of the causal relationship between IL-10, IL-18, PD-L1, and COVID-19. COVID-19 = Coronavirus disease 2019, IL-10 = interleukin-10, IL-18 = interleukin-18, PD-L1 = programmed death ligand-1.

We investigated the MR association between 91 inflammatory proteins and COVID-19 (Fig. 2A). The MR results for IL-10, interleukin-10 (IL-18) and programmed death ligand-1 (PD-L1) with COVID-19 demonstrated *P* values of <.05 for IVW, and revealed consistent causal effects across the 5 methods employed (Fig. 2B). A positive significant correlation between COVID-19 and 2 inflammatory proteins were suggested: IL-10 (OR = 1.18, 95% CI: 1.04–1.34, *P* = .01) and IL-18 (OR = 1.19, 95% CI: 1.04–1.36, *P* = .01). The results suggest that they may be risk factors for COVID-19 (Fig. 2B, C; Fig. S1, Supplement Digital Content, http://links.lww.com/MD/O525). In contrast, an inflammatory protein, PD-L1 (OR = 0.81, 95% CI: 0.68–0.95, *P* = .01), may be associated with a reduced risk of COVID-19 (Fig. 2B, C; Fig. S1, Supplement Digital Content, http://links.lww.com/MD/O525). Sensitivity analyses using heterogeneity tests, horizontal multiple validity tests, and LOO methods were conducted to validate these results (Fig. 2B; Fig. S1, Supplement Digital Content, http://links.lww.com/MD/O525).

The COVID-19 data SNPs were screened and there were no matching SNPs when *P* < 5e^−8^, so SNPs were screened with *P* < 5e^−5^. Then, clustering was performed based on chain disequilibrium (kb = 10,000 and *r*^2^ = 0.001). Reverse causality of COVID-19 with 3 suggestively related inflammatory proteins IL-10, IL-18, and PD-L1 showed none to be significant (*P* > .05; Table 1).

**Table 1:** COVID-19 with IL-10, IL-18, and PD-L1 inverse MR results.

Outcome	Exposure	Method	nsnp	*P* value
COVID-19	IL-10	MR Egger	79	.894325273
		Weighted median	79	.866466321
		Inverse variance weighted	79	.640528132
		Simple mode	79	.807741282
		Weighted mode	79	.879241311
COVID-19	IL-18	MR Egger	79	.197038294
		Weighted median	79	.832102581
		Inverse variance weighted	79	.935671718
		Simple mode	79	.880464734
		Weighted mode	79	.803519955
COVID-19	PD-L1	MR Egger	79	.459788317
		Weighted median	79	.240645677
		Inverse variance weighted	79	.149393487
		Simple mode	79	.189947377
		Weighted mode	79	.388898703

COVID-19 = Coronavirus disease 2019, IL-10 = interleukin-10, IL-18 = interleukin-18, MR = Mendelian randomization, PD-L1 = programmed death ligand-1, nsnp = number of SNPs.

### 3.2. MR findings of inflammatory proteins and blood metabolites

**Figure 3.**
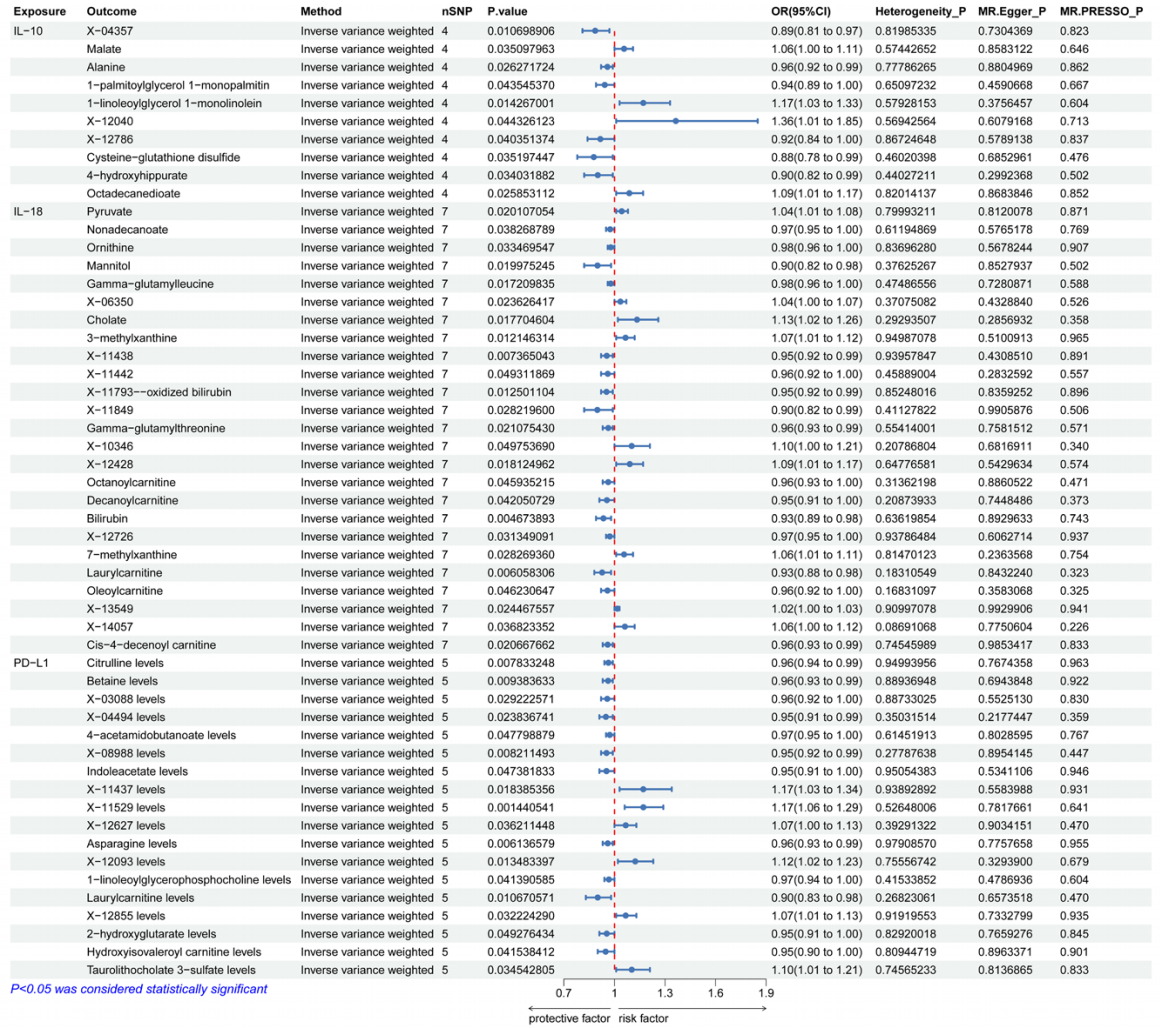
MR results on the causal relationship between 3 suggestive-related inflammatory proteins and blood metabolites. IL-10 = interleukin-10, IL-18 = interleukin-18, MR = Mendelian randomization, PD-L1 = programmed death ligand-1.

Three inflammatory proteins were associated with 486 blood metabolites with MR, and the results suggested that IL-10 was associated with 10 metabolites including 4-hydroxyhippurate, octadecanedioate, and Malate. IL-18 is associated with 25 metabolites including gamma-glutamylthreonine, 3-methylxanthine, and ornithine. PD-L1 was associated with 18 metabolites including Betaine, 4-acetamidobutanoate, and 2-hydroxyglutarate (Fig. 3; Figs. S2–S4, Supplemental Digital Content, http://links.lww.com/MD/O526). In order to check the above results, the sensitivity analyses were carried out (Fig. 3; Figs. S2–S4, Supplemental Digital Content, http://links.lww.com/MD/O526).

### 3.3. MR results of blood metabolites and COVID-19

**Figure 4.**
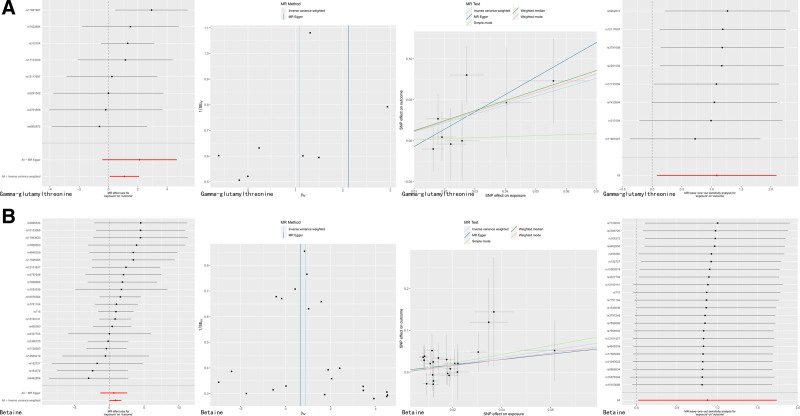
(A) Gamma-glutamylthreonine and COVID-19 MR findings. (B) Betaine and COVID-19 MR findings. COVID-19 = Coronavirus disease 2019, MR = Mendelian randomization.

MR association between blood metabolites and COVID-19, the results suggested that gamma-glutamylthreonine (OR = 2.98, 95% CI: 1.09–8.13, *P* = .034) and betaine (OR = 2.41, 95% CI: 1.02–5.69, *P* = .045) might be risk factors for COVID-19 (Table 2; Fig. 4). A sensitivity analysis was also performed to confirm this result (Fig. 4).

**Table 2:** MR results of blood metabolites and COVID-19.

Outcome	Method	nsnp	*P* value	Beta	Heterogeneity *P*	MR-Egger *P*	MR-PRESSO *P*
Gamma-glutamylthreonine	MR Egger	8	.153	2.122	.730		
	Weighted median	8	.075	1.226			
	IVW	8	.034	1.090	.738	.420	.854
	Simple mode	8	.910	0.125			
	Weighted mode	8	.230	1.169			
Betaine	MR Egger	22	.518	0.645	.913		
	Weighted median	22	.140	0.879			
	IVW	22	.045	0.879	.935	.793	.954
	Simple mode	22	.271	1.062			
	Weighted mode	22	.303	0.762			

COVID-19 = Coronavirus disease 2019, IVW = inverse variance weighted, MR = Mendelian randomization, nsnp = number of SNPs.

### 3.4. Intermediary effect

We performed a 2-step MR analysis to examine the mediating pathways from IL-18 to COVID-19 by gamma-glutamylthreonine, and PD-L1 to COVID-19 by Betaine. The results suggest that betaine may be mediating the causation between PD-L1 and COVID-19, with a mediation rate of 15.92% (Table 3).

**Table 3:** Mediation of COVID-19 by blood metabolites through inflammatory proteins.

Outcome	Mediator	Total effect	Mediation effect	Direct effect	Mediated proportion (%)
PD-L1	Betaine	−0.22	−0.04	−0.18	15.92

COVID-19 = Coronavirus disease 2019, PD-L1 = programmed death ligand-1.

## 4. Discussion

Within a full-scale mediated MR study, a cause-and-effect association between 3 inflammatory proteins and COVID-19 has been identified. Mediated MR results showed that betaine may account for 15.92% of the effect of PD-L1 on COVID-19. This analysis highlighted the link between inflammatory proteins and COVID-19, highlighting the mediating role of the blood metabolite, betaine.

A substantial body of literature has emerged examining the role of inflammatory factors and blood metabolites in the SARS-CoV-2 pandemic. Infection with SARS-CoV-2 has the potential to trigger a widespread systemic inflammatory response, which may result in severe complications such as multi-organ dysfunction, particularly acute respiratory distress syndrome, myocarditis and sepsis. During infection, there was a notable increase in the production and release of pro-inflammatory cytokines, including IL-8, TNF-α, and IL-6. These cytokines collectively drove an excessive inflammatory response.^[17,18]^ In particular, this phenomenon was observed in critically ill patients with COVID-19, where it contributed to immune system dysregulation. Similarly, significant elevations of pro-inflammatory factors, including IL-1β, IL-6, CCL4, and CXCL10, as well as abnormal increases in serum IL-6 levels, were observed in a mouse model of H1N1 influenza virus infection.^[19]^ In light of these observations, investigators treated patients with confirmed or suspected SARS-CoV-2 infection with IL-1 or IL-6 antagonists and found that these inhibitors were effective in mitigating the acute inflammatory response.^[20]^ Specifically, SARS-CoV-2 infection promotes the secretion of TNF, IL-6, and other pro-inflammatory cytokines through activation of the IL-1 pathway, which in turn has deleterious effects on lung and systemic health.^[21]^ Consequently, the inhibition of hyperactive inflammatory factors may prove an efficacious therapeutic strategy for the attenuation of severe inflammatory responses triggered by the virus and the improvement of clinical prognosis.

In recent years, a substantial body of research has been published on the alterations in metabolites observed in patients with COVID-19. A number of studies have demonstrated that tryptophan metabolism is significantly impaired^[22]^ and that butyric acid levels were significantly reduced^[23]^ in patients diagnosed with COVD-19. Furthermore, a reduction in the expression of transmembrane serine protease 2 may contribute to a reduction in the severity of the disease, due to a decrease in the levels of the SARS-CoV-2 virus in the body.^[24]^ With regard to metabolites, there was evidence of enrichment in ribonucleic acid and lactic acid in patients with SARS-CoV-2 infection. The accumulation of ribonucleic acid may be indicative of intestinal damage and associated alterations in metabolic pathways, thereby suggesting that it may play a pivotal role in the pathological process of SARS-CoV-2 infection. Lactic acid, a byproduct of anaerobic metabolism, may play a pivotal role in the immune response and pathological progression of SARS-CoV-2 infection by modulating the immune response and influencing intestinal microecological imbalance.^[25]^

The present study has identified a significant correlation between IL-10, IL-18, PD-L1, gamma-glutamylthreonine, betaine, and COVD-19, which may provide a novel insight into the underlying mechanisms involved in the disease process.

Interleukin-10 (IL-10) was an essential anti-inflammatory factor involved in the regulation of a wide range of cellular functions and played a central role in infection by limiting or terminating the immune response to inflammation.^[26]^ It is frequently elevated in inflammatory and tumor microenvironments. A notable correlation has been established between the CD14^+^ HLA-DR^−^ immunosuppressive myeloid-derived dendritic cells subpopulation and the systemic cytokine IL-10.^[27]^ Inflammatory cytokines were detected in serum samples from COVID-19 patients, and IL-10 levels were significantly elevated,^[28]^ and expression increased with increasing disease severity.^[28]^ It has therefore been suggested that IL-10 associated with poorer prognosis and mortality in COVID-19 patients was a predictor of disease.^[29]^ However, other studies found reduced levels of IL-10 during COVID-19 infection, with recovered patients expressing higher levels of IL-10 compared to SARS-CoV-2 infected patients.^[30]^ Therefore, the results regarding the expression level of IL-10 among COVID-19 patients have not been consistent. And our results supported the idea that IL-10 may be a risk factor for COVID-19.

Interleukin-18 (IL-18) was a cytokine that stimulated IFN-γ production by Th 1 cells, belonging to the IL-1 family, and was produced by macrophages early in the inflammatory immune response, playing a broad role in defense against infection.^[31]^ SARS-CoV-2 infection and replication in lung macrophages were key drivers of disease.^[32]^ Activation of inflammatory vesicles during SARS-CoV-2 infection released pro-inflammatory factors, including IL-18, causing excessive inflammatory load in the lungs and leading to tissue damage. Studies have shown that IL-18 levels were elevated in COVID-19 patients and correlated with disease severity and poor clinical outcomes.^[33]^ Therefore, it has been suggested that IL-18 may be a marker for predicting poor prognosis in COVID-19.^[34]^ The enzymes and metabolites involved in the extracellular degradation and intracellular synthesis of glutathione constituted the gamma-glutamyl cycle.^[35]^ Gamma-glutamyl peptide was a key product of this cycle and had been identified as a potential biomarker for aging, liver disease, and cancer.^[36–38]^ Gamma-glutamylthreonine was a product of circulating catalytic catabolism. The current study found gamma-glutamylthreonine to be a risk factor for prostate cancer,^[39]^ a protective factor for systemic lupus erythematosus,^[40]^ and the best categorical variable for new cases of tuberculosis.^[41]^ Although our study implied that gamma-glutamylthreonine may not be a mediator of the causal relationship between IL-18 and COVID-19, it also suggested that gamma-glutamylthreonine may exacerbate the course of COVID-19.

The most important diagnostic feature of COVID-19 was lymphocyte depletion, especially T-cell depletion. Whereas immunosuppressive molecules (programmed death-1 [PD-1] and programmed death ligand-1 [PD-L1]) expressed on the surface of many cells including T-cells, B-cells, monocytes, macrophages, and T-cells of natural killers, respectively, have played an important role in the innate immune response, especially the adaptive immune response.^[42]^ PD-L1 was a negative regulator of T cell activation.^[43]^ Many studies have reported that severe and critically ill COVID-19 patients exhibit dysregulation of the PD-1/PD-L1 axis with significantly elevated blood levels of PD-L1.^[44]^ Many researchers have identified increased PD-L1 expression as directly correlating with disease severity^[45]^ and as a prognostic marker and therapeutic target for severe COVID-19.^[46]^ However knocking down PD-L1 expression resulted in the development of autoimmune disease.^[47]^ Blocking the PD-1/PD-L1 axis may theoretically increase the period of overactive immunity in COVID-19 and worsen outcomes.^[48]^ A recent study found that while PD-L1 promotes immune escape in the early stages of COVID-19, it may suppress excessive immune responses as the disease progresses.^[49]^ And our big data-based screening suggested that PD-L1 may inhibit the development of COVID-19. This provided a new idea for its use as a disease prognostic molecule and improved our understanding of the underlying mechanisms of immune system function. Betaine, known as trimethylglycine, a metabolite of choline, was involved in methylation as a methyl donor and possessed anti-inflammatory properties in many diseases.^[50]^ Our study suggests that betaine played a mediating effect in the protective effect of PD-L1 against COVID-19 with a mediating percentage of 15.92%.

This was the first study to conduct a large-scale MR analysis of the causal relationship between inflammatory proteins, blood metabolites and COVID-19. The initial results of our study suggest a fact that there was a causal relationship between inflammatory proteins and COVID-19, in which blood metabolites may play a mediating role, which provided further new ideas for the treatment and prevention of COVID-19. However, our study has some limitations. Firstly, participation in this were European populations, which may produce racial differences. Second, the results did not pass the FDR correction and were indicative, requiring further cellular, animal and clinical experiments for validation.

## 5. Conclusions

Our MR study showed a potential causal relationship between inflammatory proteins, blood metabolites and COVID-19. Specifically, Betaine mediated the regulatory effect of PD-L1 on COVID-19. Our results provided genetic evidence for the immune mechanism of COVID-19 and new possibilities for disease prevention and treatment.

## Acknowledgments

The research community is thanked for making summary statistics from genome-wide association studies publicly available.

## Author contributions

**Conceptualization:** Yuling Chen, Chang Chen.

**Data curation:** Chang Chen.

**Formal analysis:** Chang Chen.

**Funding acquisition:** Yuling Chen.

**Investigation:** Yuling Chen.

**Methodology:** Yuling Chen.

**Project administration:** Yuling Chen.

**Resources:** Yuling Chen.

**Software:** Chang Chen.

**Supervision:** Yuling Chen.

**Validation:** Yuling Chen, Chang Chen.

**Visualization:** Yuling Chen.

**Writing – original draft:** Yuling Chen.

**Writing – review & editing:** Yuling Chen, Chang Chen.

## Supplementary Material


