# Supplementary material for: The effect of inflammatory proteins on COVID-19 is mediated by blood metabolites: A Mendelian randomization study
Source: Medicine (Baltimore). 2025 Mar 14;104(11):e41852. doi: 10.1097/MD.0000000000041852 (PMC11922457; doi:10.1097/MD.0000000000041852)
Supplement: Supplementary file 1 [file medi-104-e41852-s001.docx]

**S1. Resultant plots of 3 suggestively associated inflammatory proteins with COVID-19. (A), funnel plot (B), scatter plot (C), leave-one-out plot**

1. **
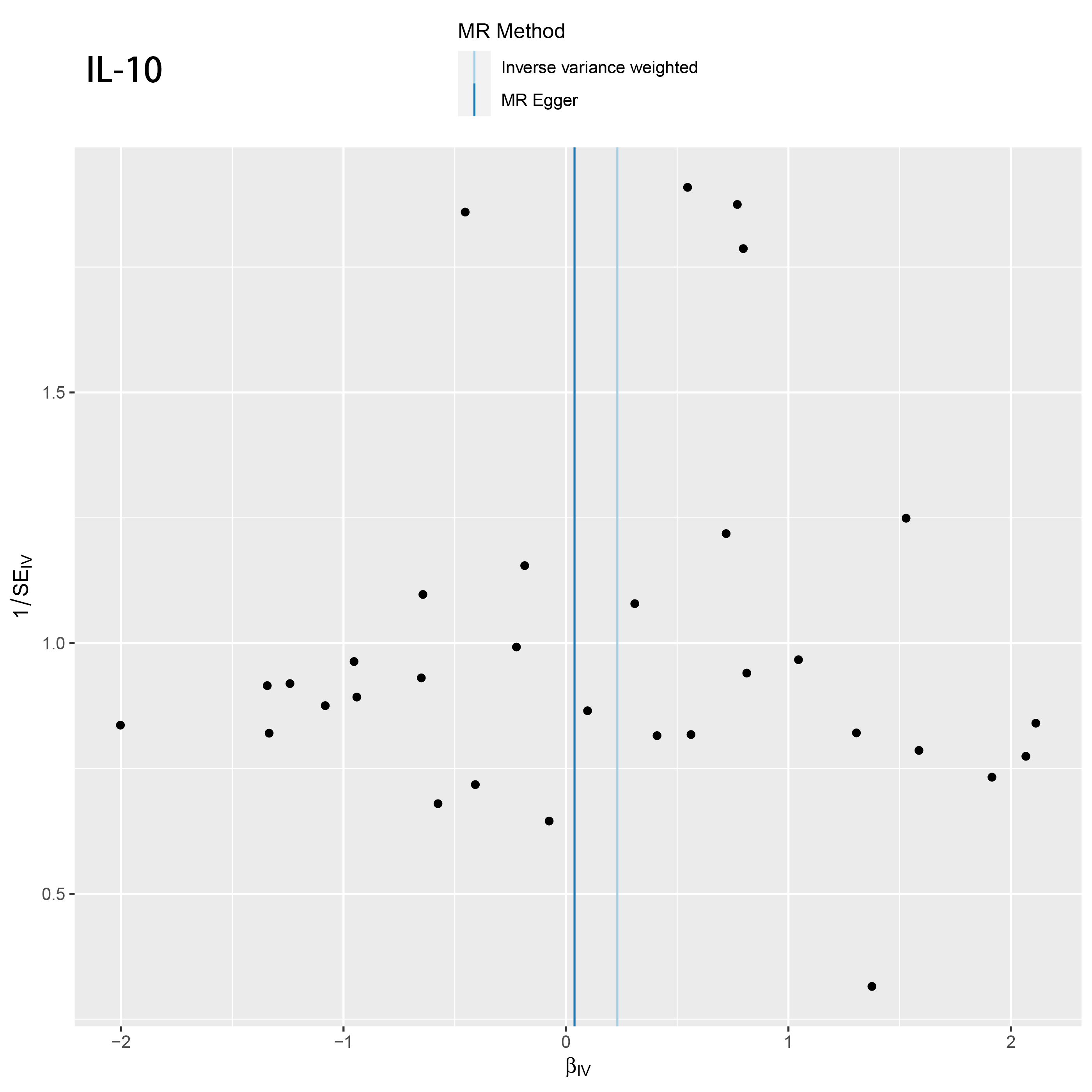

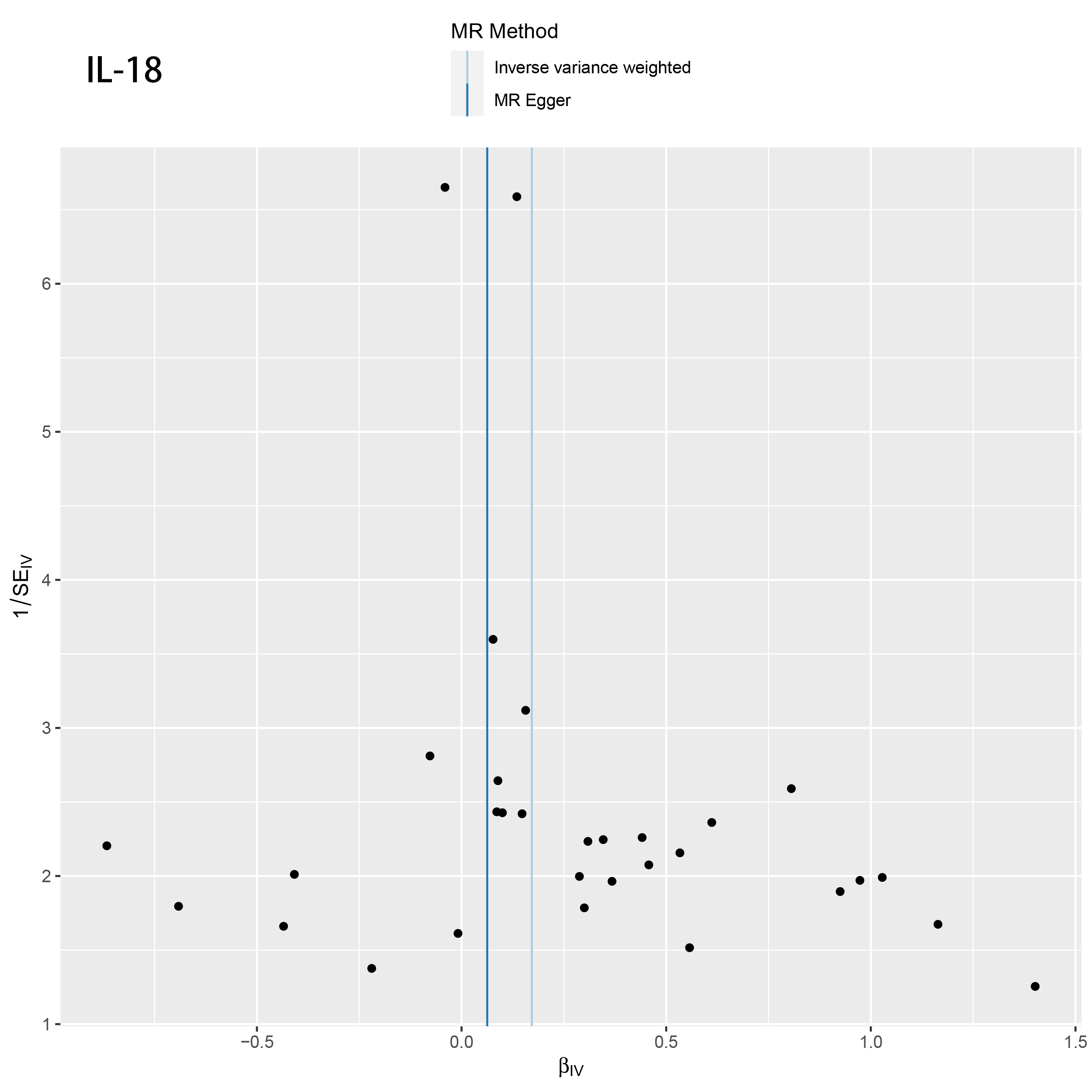

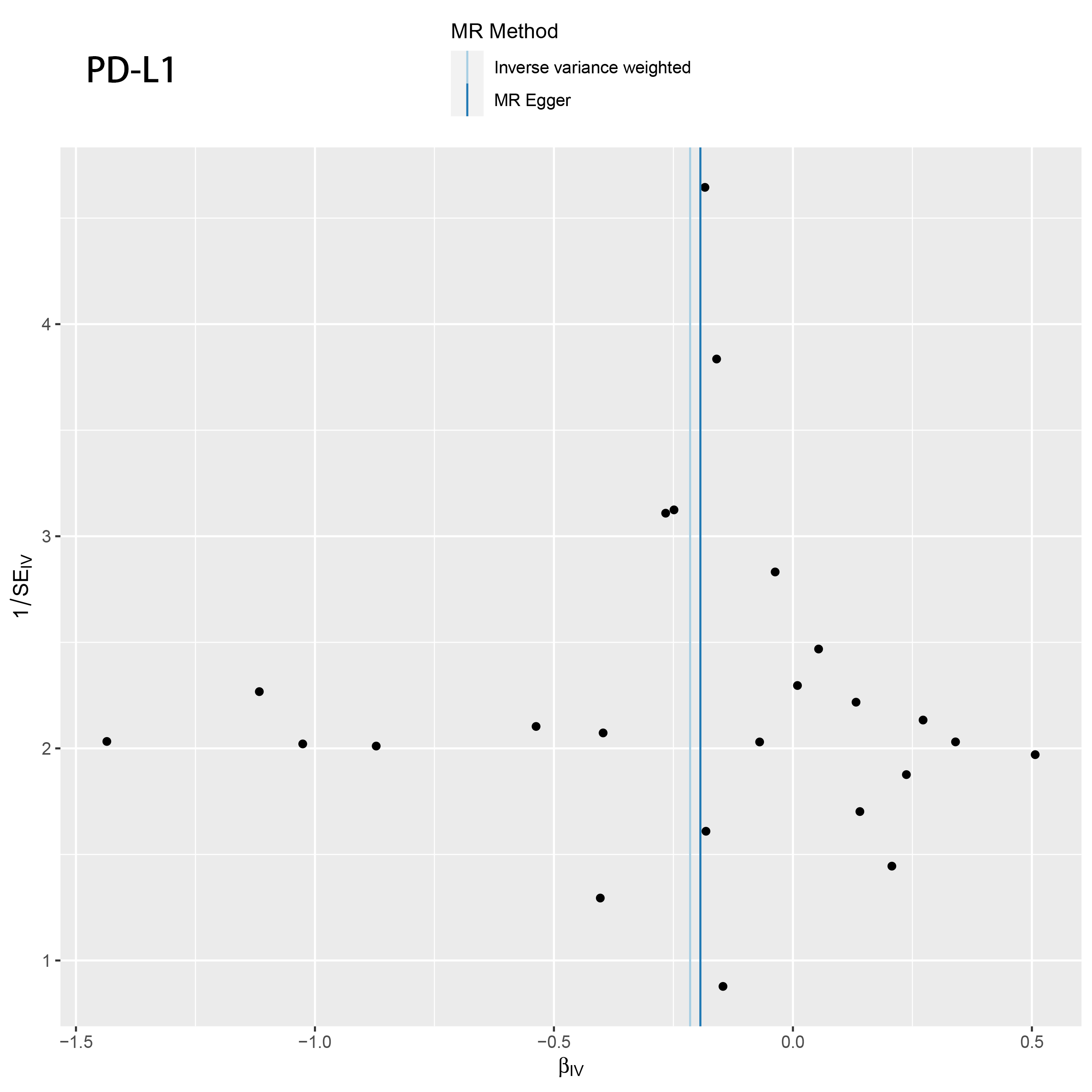
**
2. **
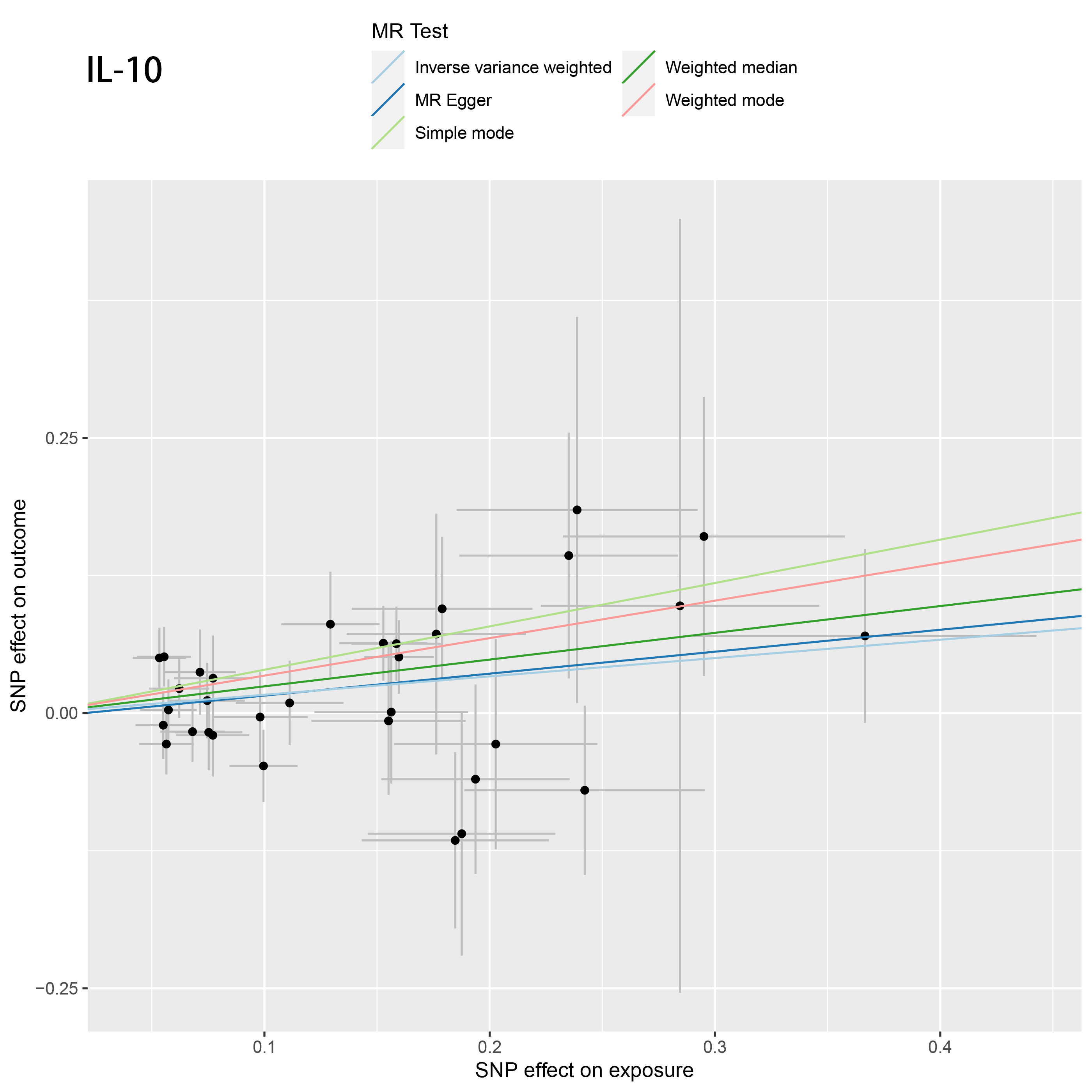

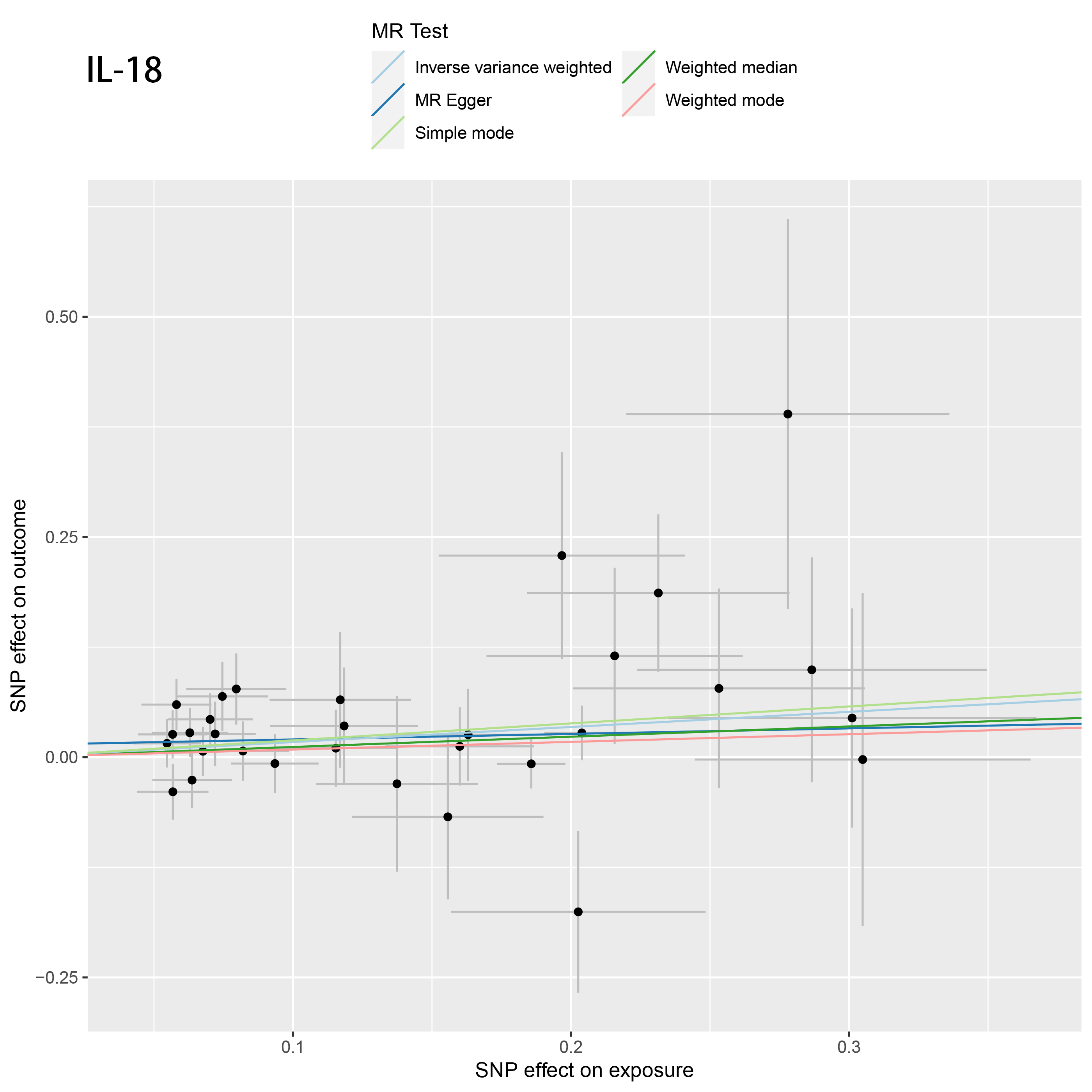

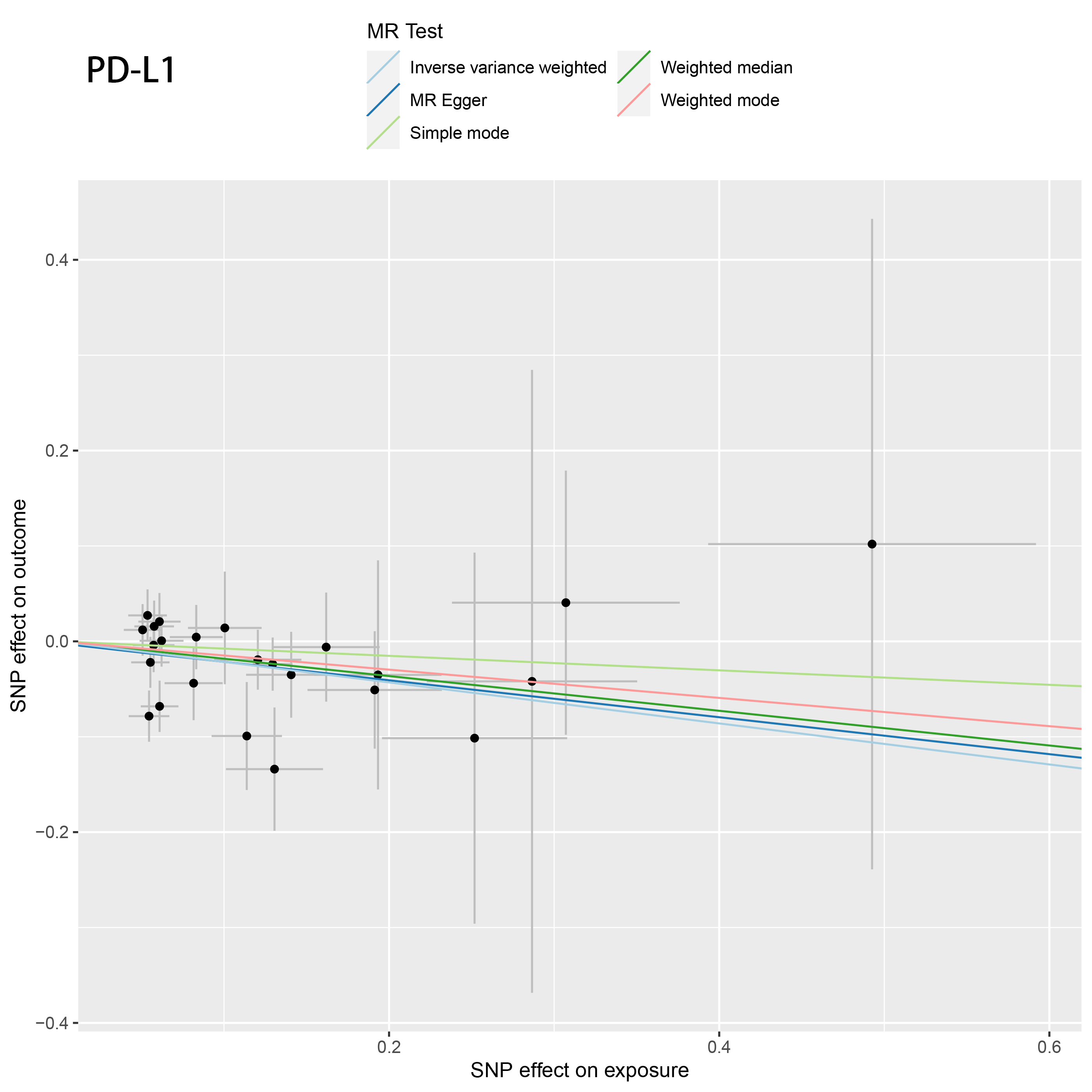
**
3. **
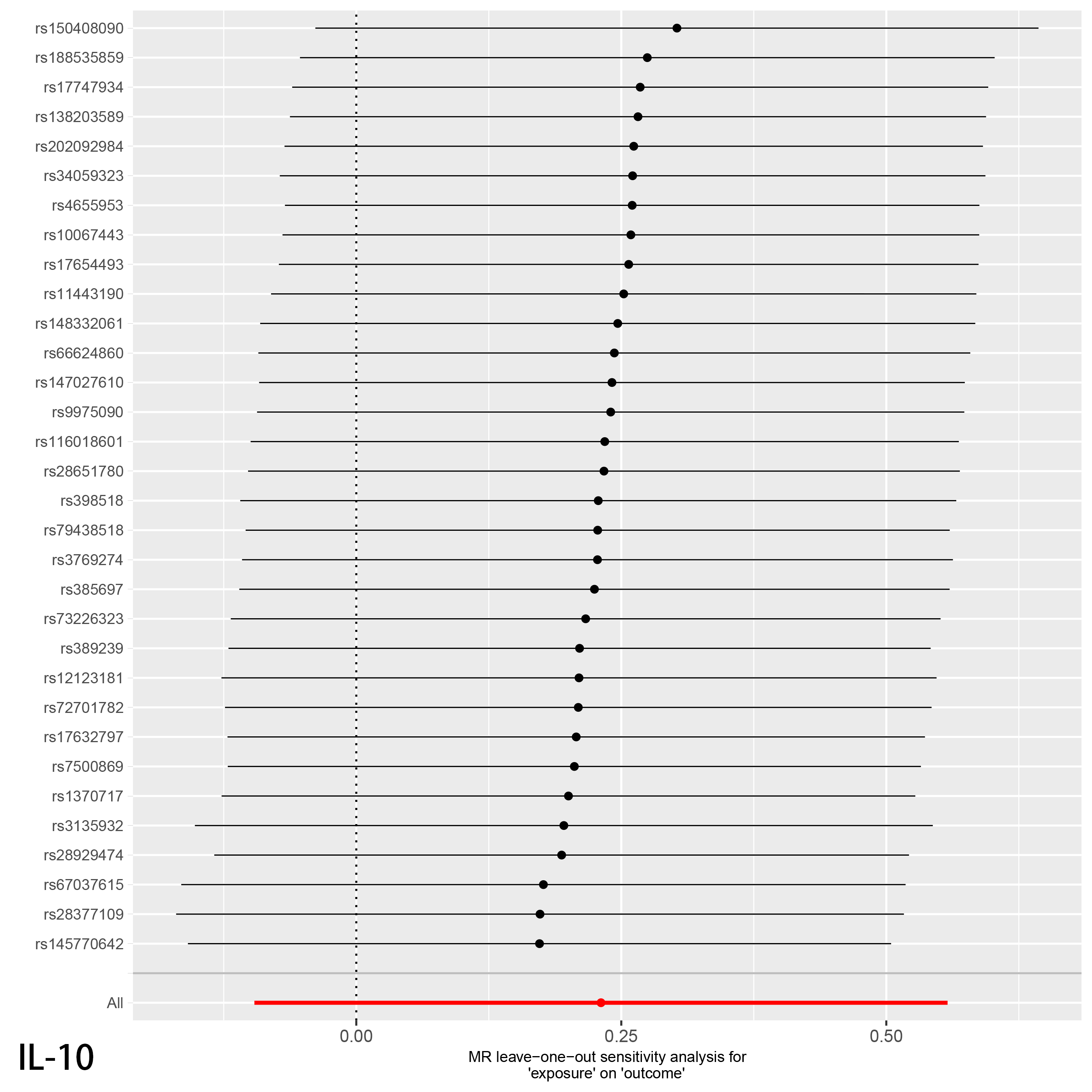

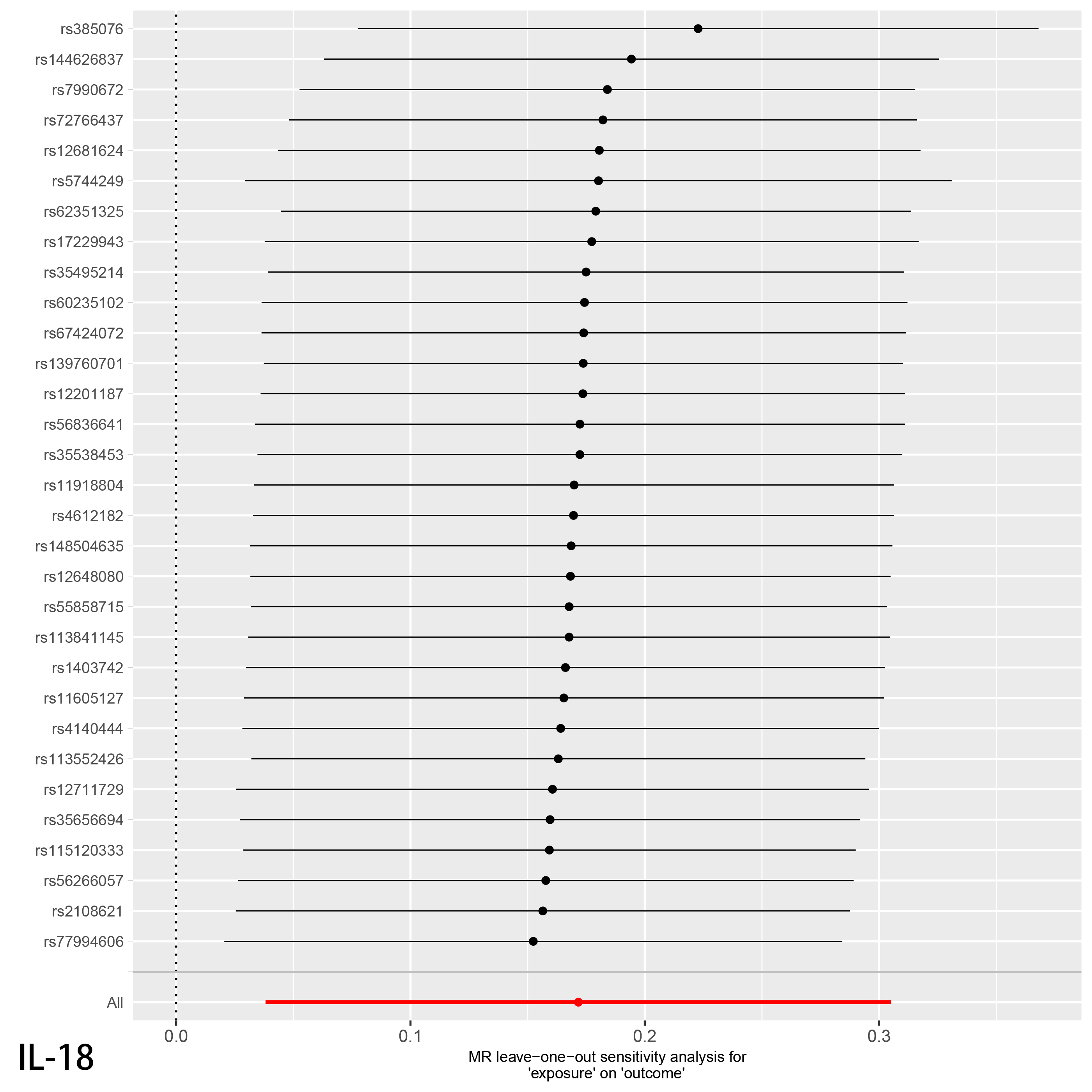

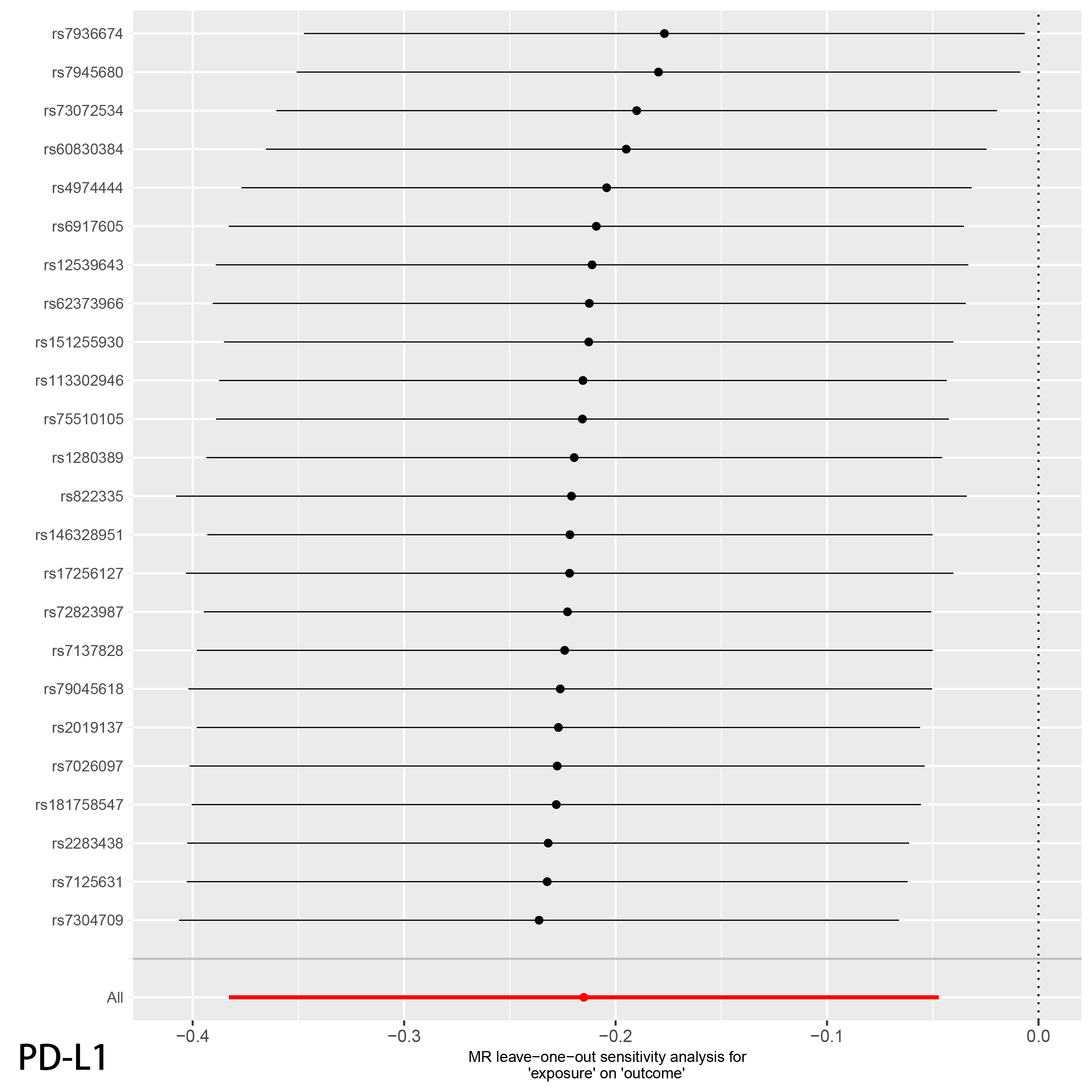
**
