# Supplementary material for: The effect of inflammatory proteins on COVID-19 is mediated by blood metabolites: A Mendelian randomization study
Source: Medicine (Baltimore). 2025 Mar 14;104(11):e41852. doi: 10.1097/MD.0000000000041852 (PMC11922457; doi:10.1097/MD.0000000000041852)
Supplement: Supplementary file 2 [file medi-104-e41852-s002.pdf]

S2. IL-10 versus blood metabolites result plots. (A), forest plots (B), funnel plots (C), scatter plots (D), leave-one-out plots

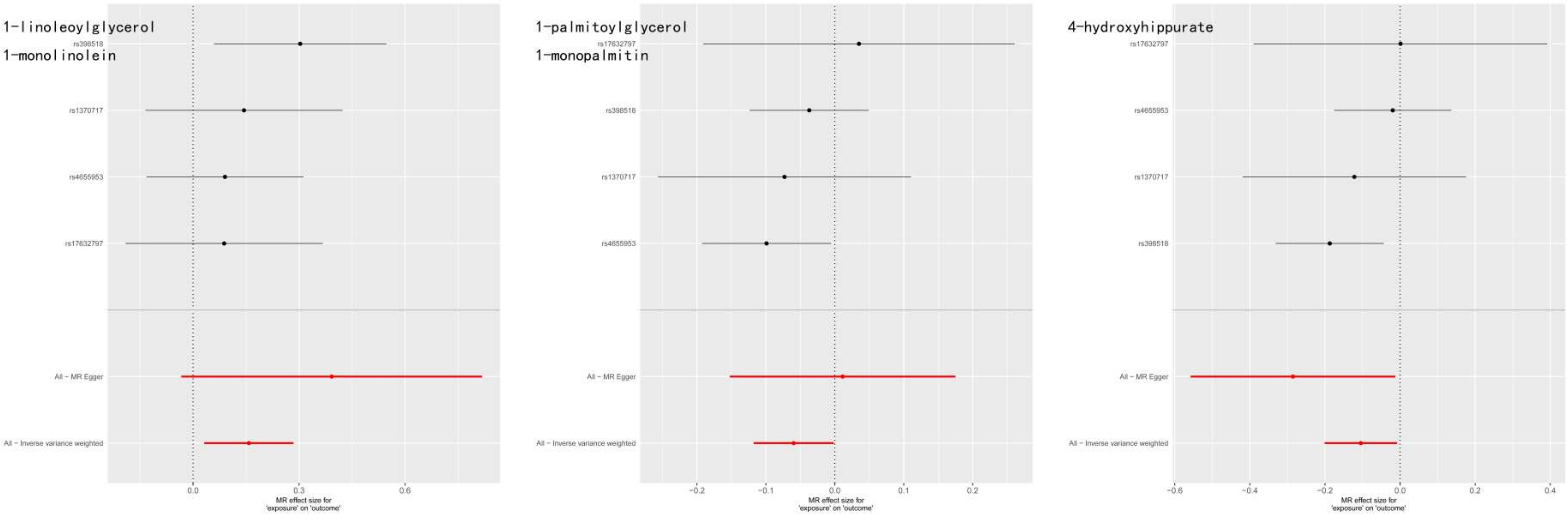

A.

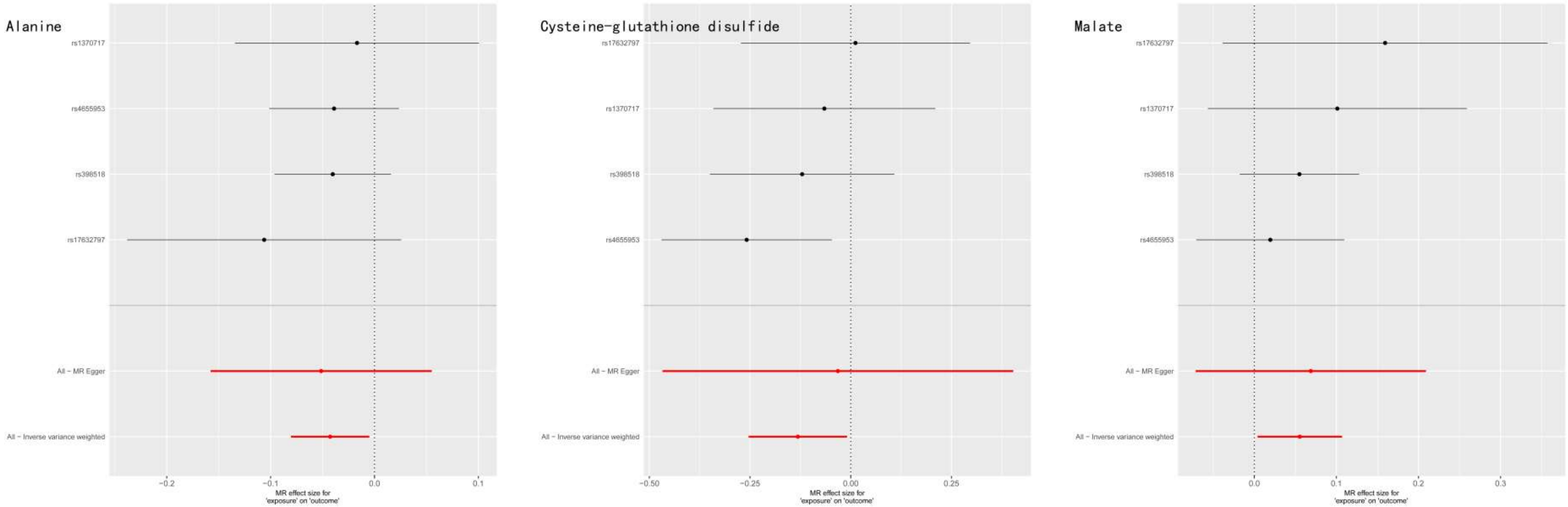

Alanine
rs1370717
rs4655953
rs398518
rs17632797
All − MR Egger
All − Inverse variance weighted
−0.2
−0.1
0.0
0.1
MR effect size for
'exposure' on 'outcome'
Cysteine-glutathione disulfide
rs17632797
rs1370717
rs398518
rs4655953
All − MR Egger
All − Inverse variance weighted
−0.50
−0.25
0.00
0.25
MR effect size for
'exposure' on 'outcome'
Malate
rs17632797
rs1370717
rs398518
rs4655953
All − MR Egger
All − Inverse variance weighted
0.0
0.1
0.2
0.3
MR effect size for
'exposure' on 'outcome'

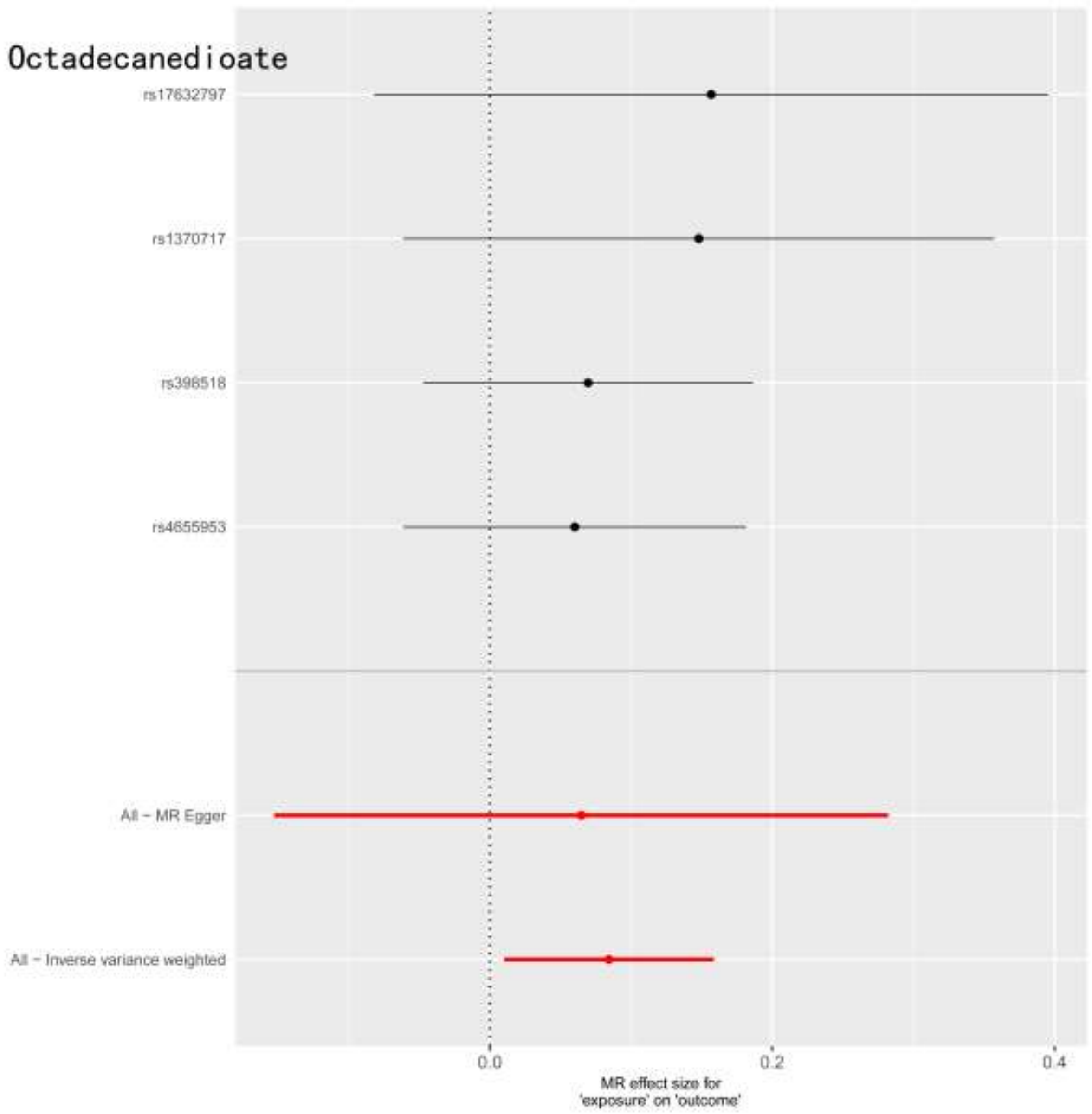

Octadecanedioate
rs17632797
rs1370717
rs398518
rs4655953
All − MR Egger
All − Inverse variance weighted
0.0
0.2
0.4
MR effect size for
'exposure' on 'outcome'

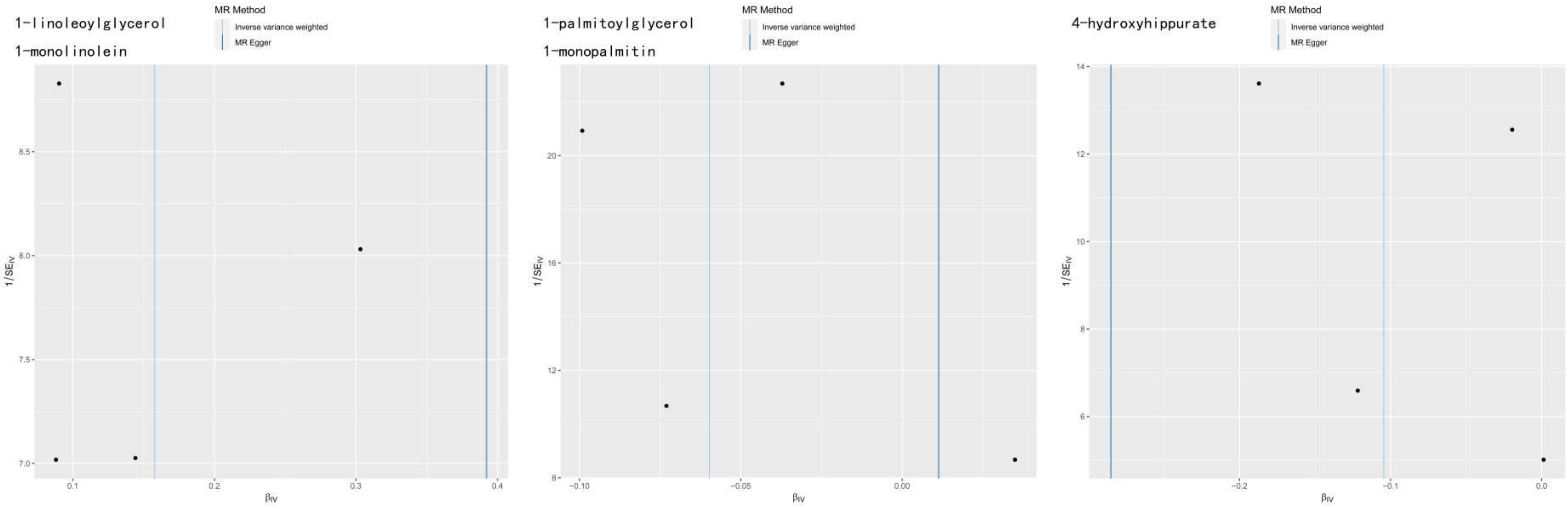
1-linoleoylglycerol
1-monolinolein
MR Method
Inverse variance weighted
MR Egger
1/SE_IV
β_IV
1-palmitoylglycerol
1-monopalmitin
MR Method
Inverse variance weighted
MR Egger
1/SE_IV
β_IV
4-hydroxyhippurate
MR Method
Inverse variance weighted
MR Egger
1/SE_IV
β_IV

B.

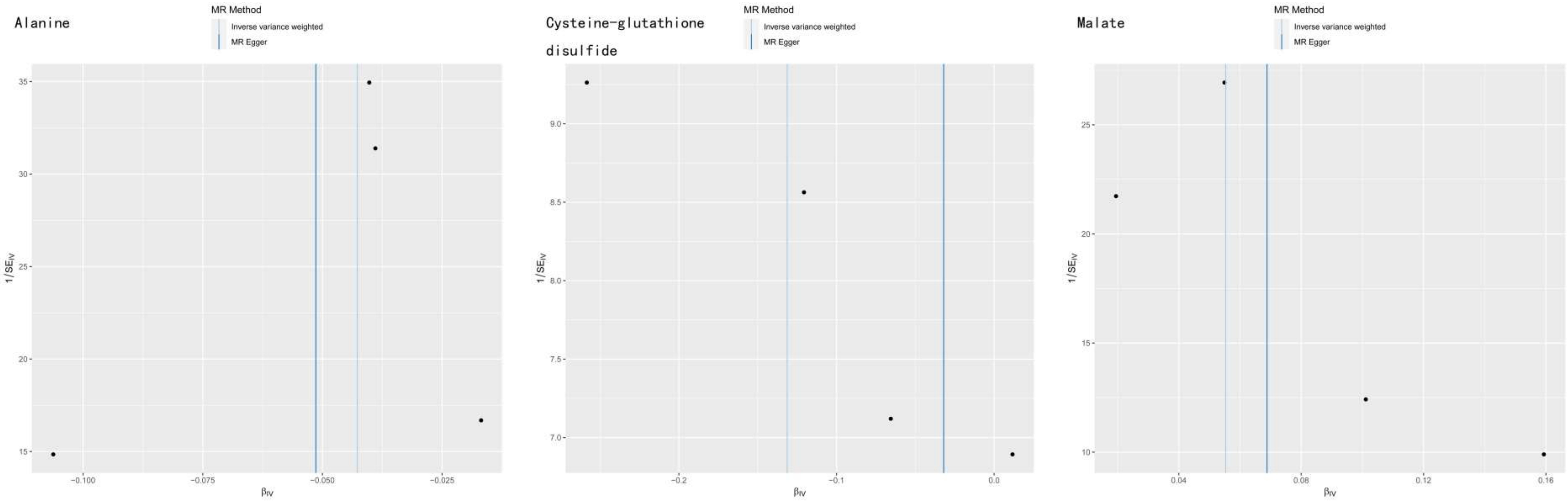
Alanine
MR Method
Inverse variance weighted
MR Egger
1/SE_IV
β_IV
Cysteine-glutathione disulfide
MR Method
Inverse variance weighted
MR Egger
1/SE_IV
β_IV
Malate
MR Method
Inverse variance weighted
MR Egger
1/SE_IV
β_IV

Octadecanedioate

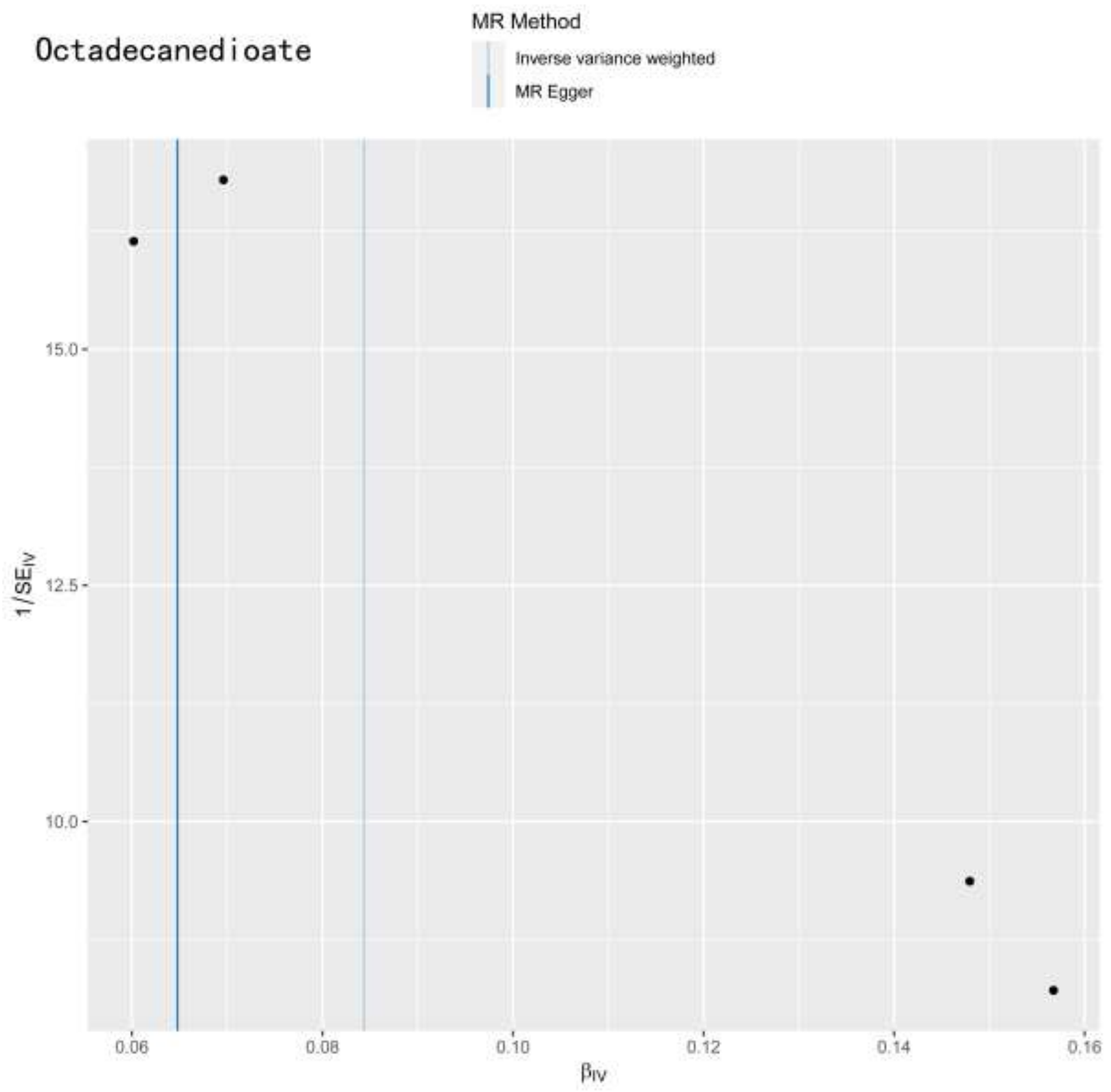

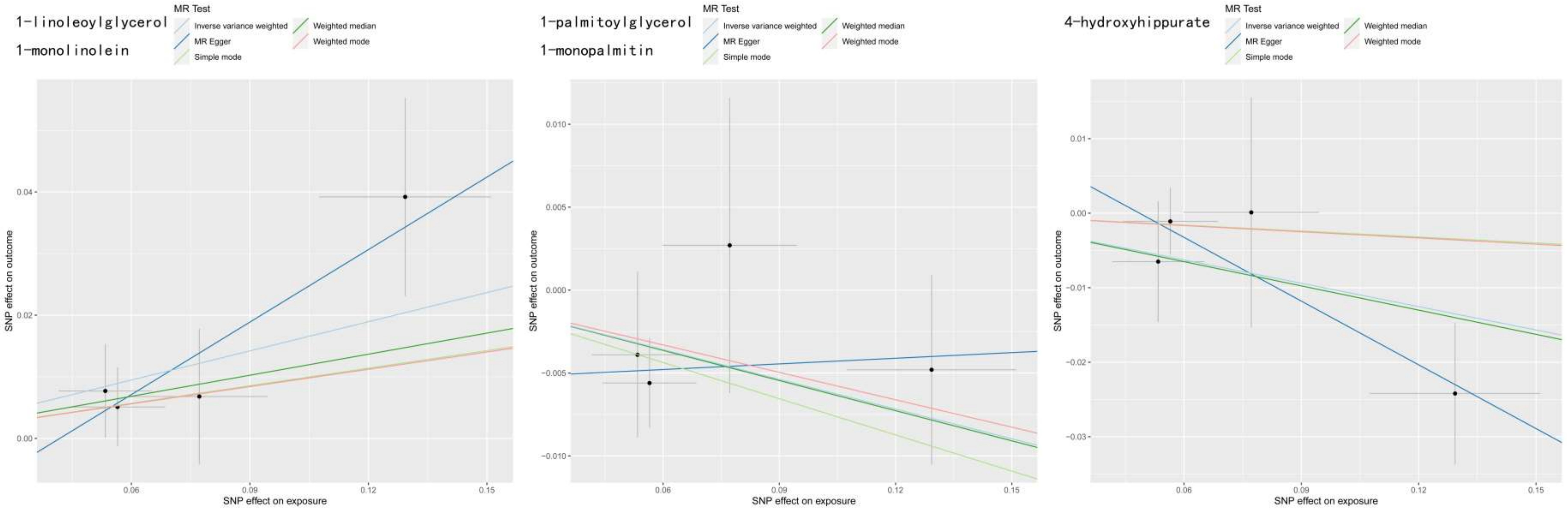

1-linoleoylglycerol
1-monolinolein
MR Test
Inverse variance weighted
MR Egger
Simple mode
Weighted median
Weighted mode
SNP effect on outcome
SNP effect on exposure
1-palmitoylglycerol
1-monopalmitin
4-hydroxyhippurate

C.

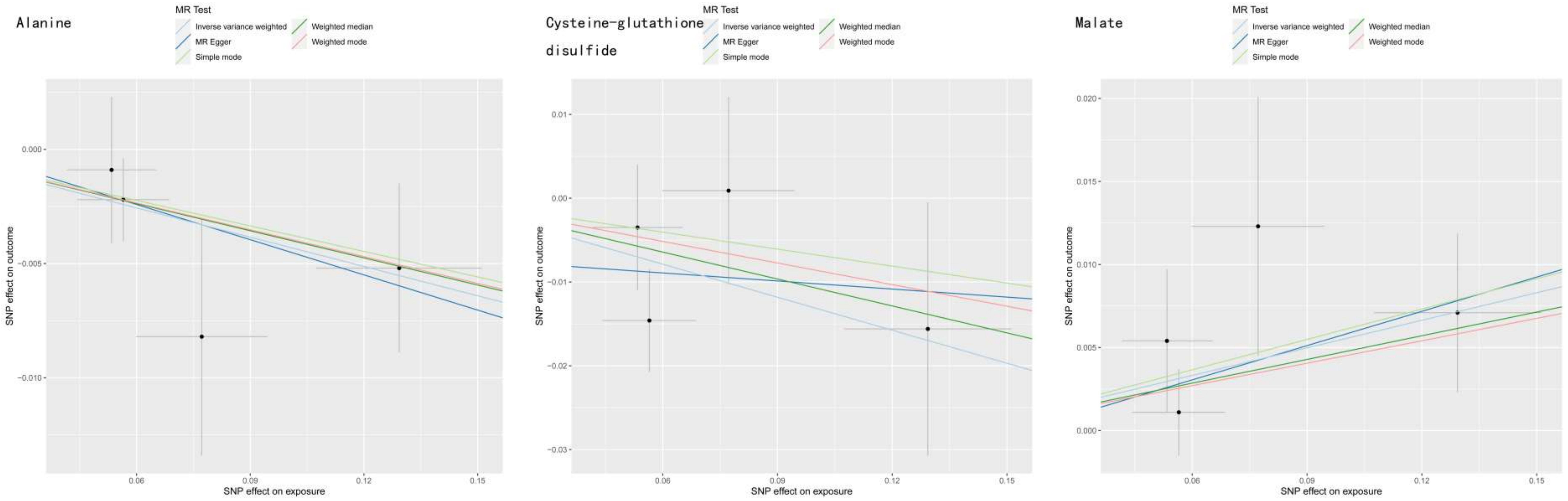

Alanine
MR Test
Inverse variance weighted
MR Egger
Simple mode
Weighted median
Weighted mode
SNP effect on outcome
SNP effect on exposure
Cysteine-glutathione disulfide
Malate

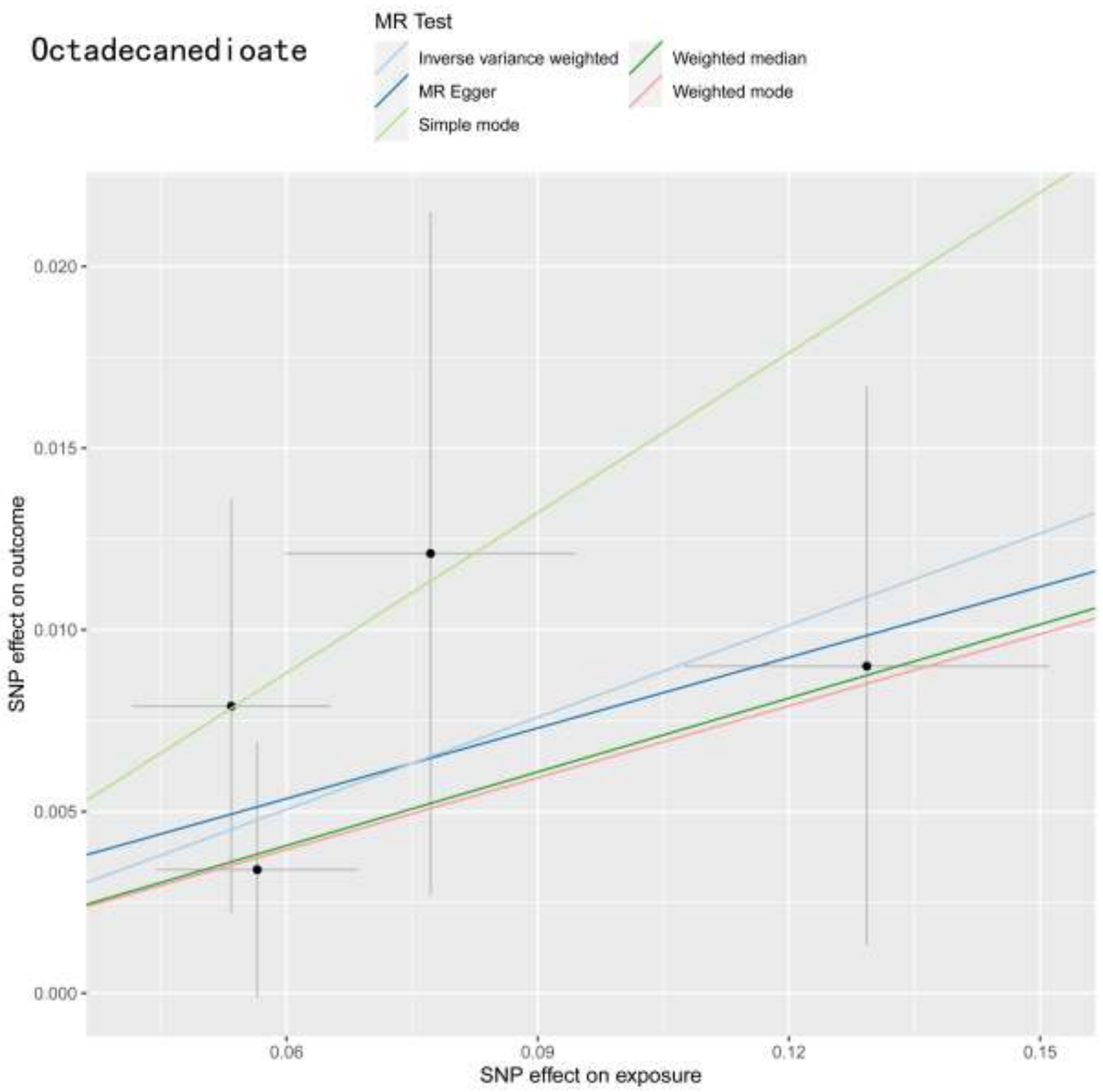

Octadecanedioate
MR Test
Inverse variance weighted
MR Egger
Simple mode
Weighted median
Weighted mode
0.020
0.015
0.010
0.005
0.000
SNP effect on outcome
0.06
0.09
0.12
0.15
SNP effect on exposure

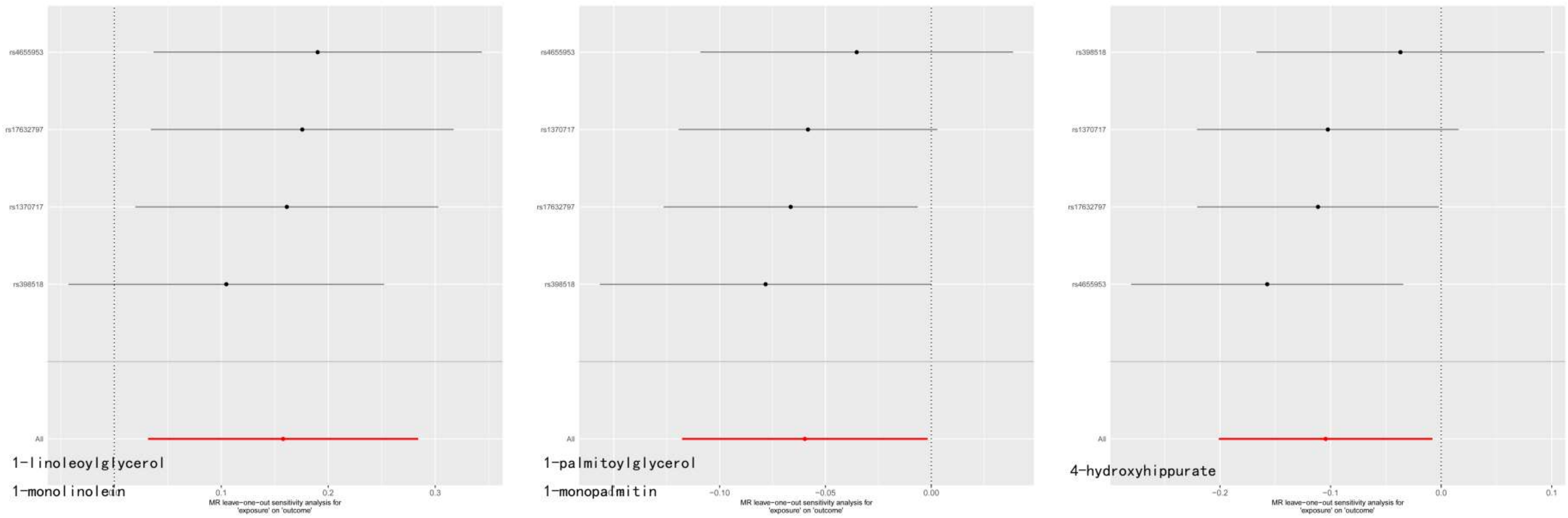
rs4655953
rs17632797
rs1370717
rs398518
All
1-linoleoylglycerol
1-monolinolein
0.1
0.2
0.3
MR leave-one-out sensitivity analysis for
'exposure' on 'outcome'
rs4655953
rs1370717
rs17632797
rs398518
All
1-palmitoylglycerol
1-monopalmitin
−0.10
−0.05
0.00
MR leave-one-out sensitivity analysis for
'exposure' on 'outcome'
rs398518
rs1370717
rs17632797
rs4655953
All
4-hydroxyhippurate
−0.2
−0.1
0.0
0.1
MR leave-one-out sensitivity analysis for
'exposure' on 'outcome'

D.

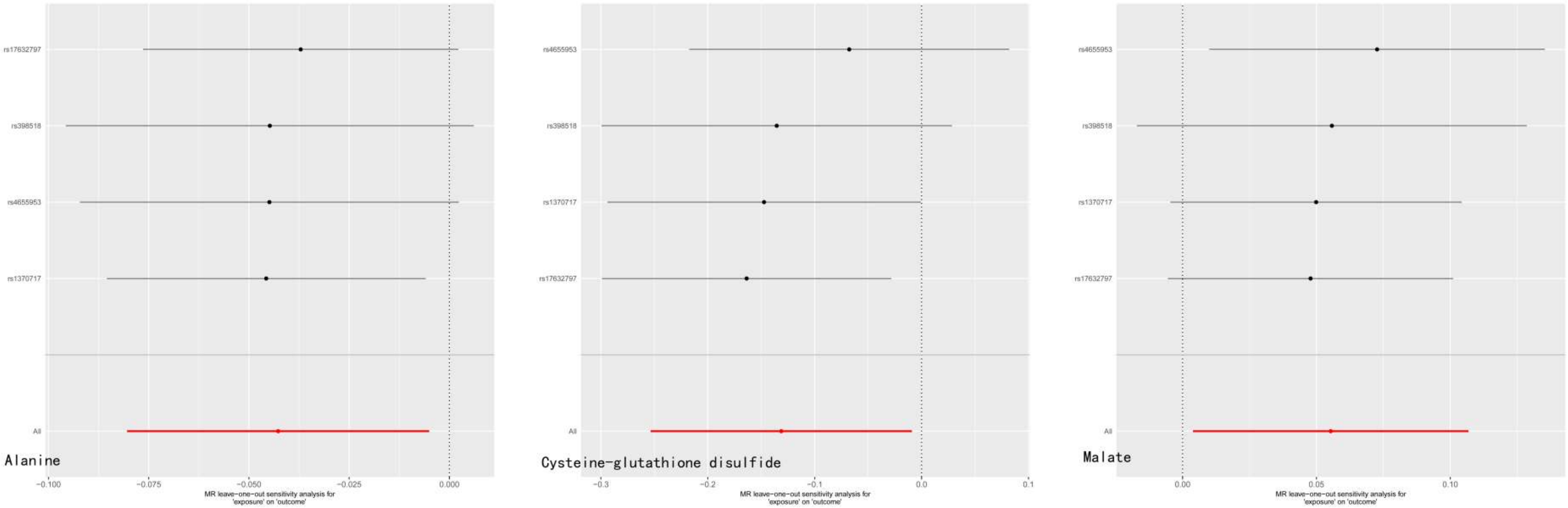
Alanine
rs17632797
rs398518
rs4655953
rs1370717
All
−0.100
−0.075
−0.050
−0.025
0.000
MR leave−one−out sensitivity analysis for 'exposure' on 'outcome'
Cysteine-glutathione disulfide
rs4655953
rs398518
rs1370717
rs17632797
All
−0.3
−0.2
−0.1
0.0
0.1
MR leave−one−out sensitivity analysis for 'exposure' on 'outcome'
Malate
rs4655953
rs398518
rs1370717
rs17632797
All
0.00
0.05
0.10
MR leave−one−out sensitivity analysis for 'exposure' on 'outcome'

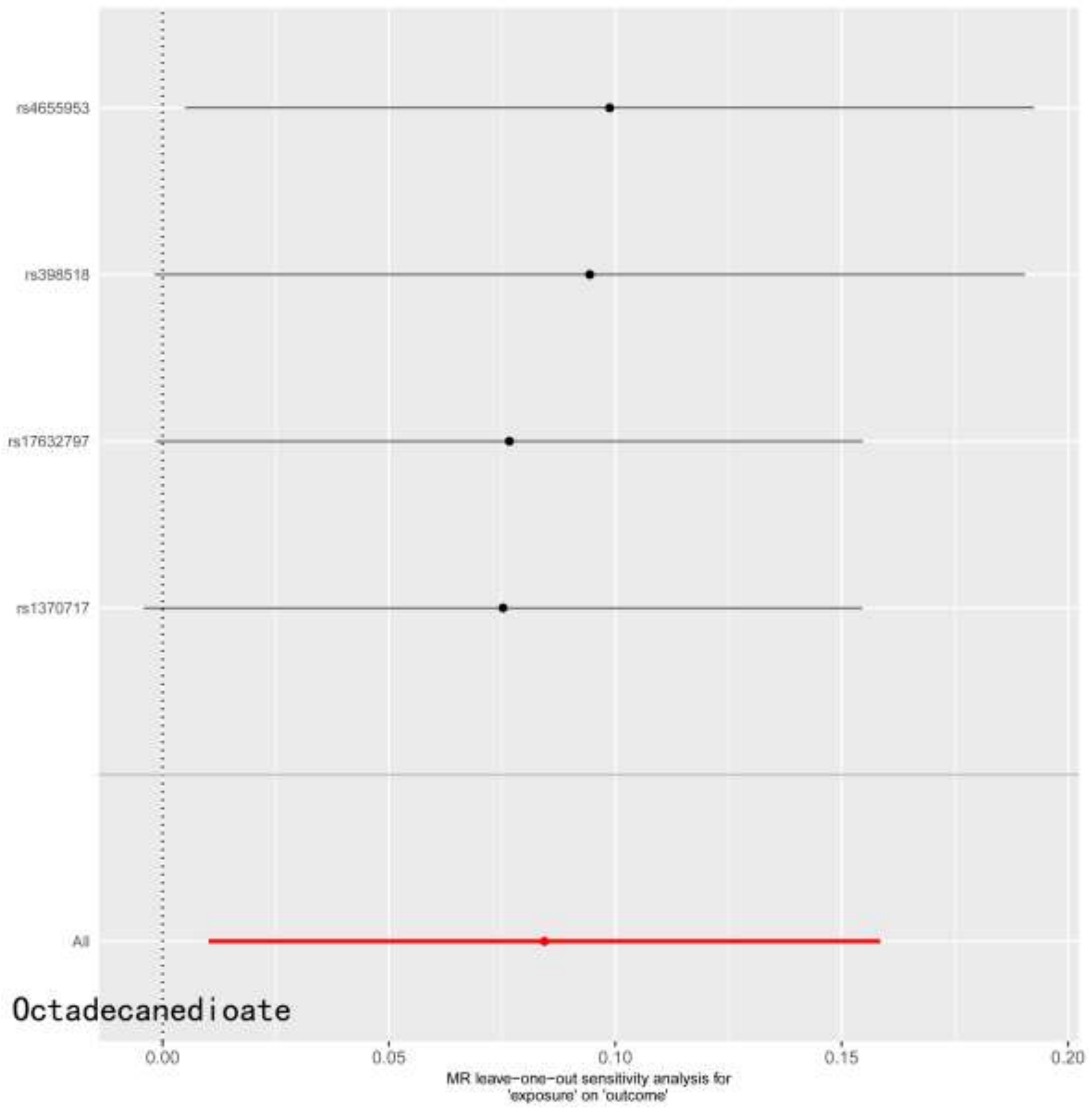

rs4655953
rs398518
rs17632797
rs1370717
All
Octadecanedioate
0.00
0.05
0.10
0.15
0.20
MR leave−one−out sensitivity analysis for
'exposure' on 'outcome'

S3. IL-18 versus blood metabolites result plots. (A), forest plots (B), funnel plots (C), scatter plots (D), leave-one-out plots

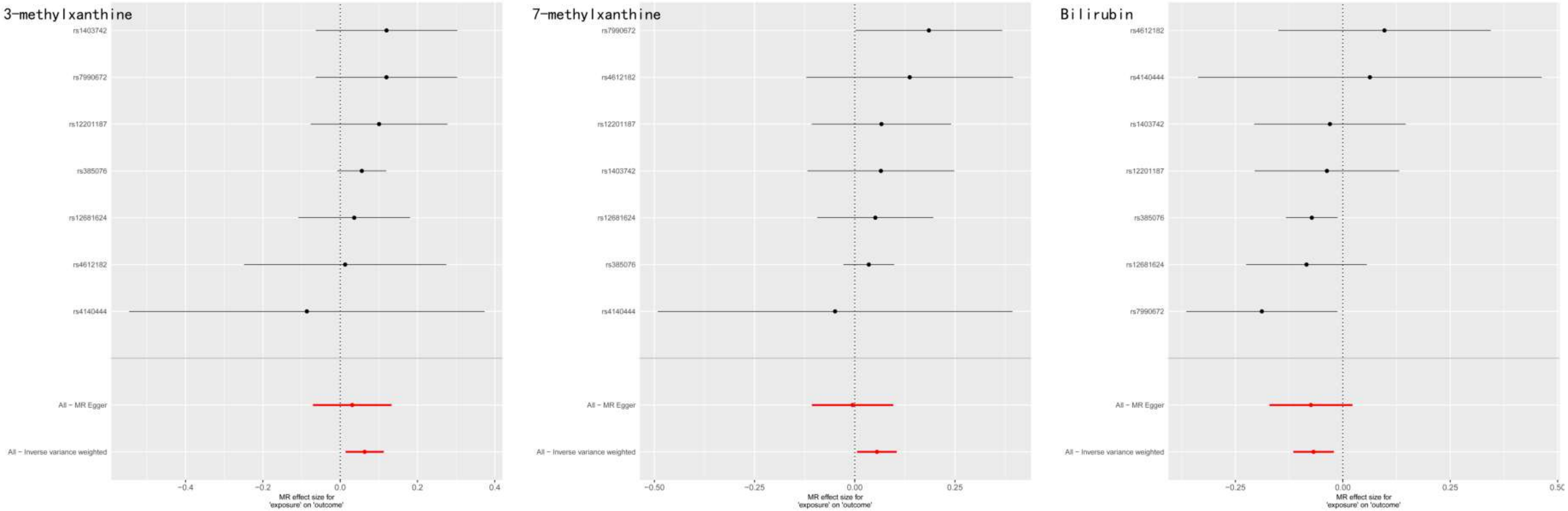

A.

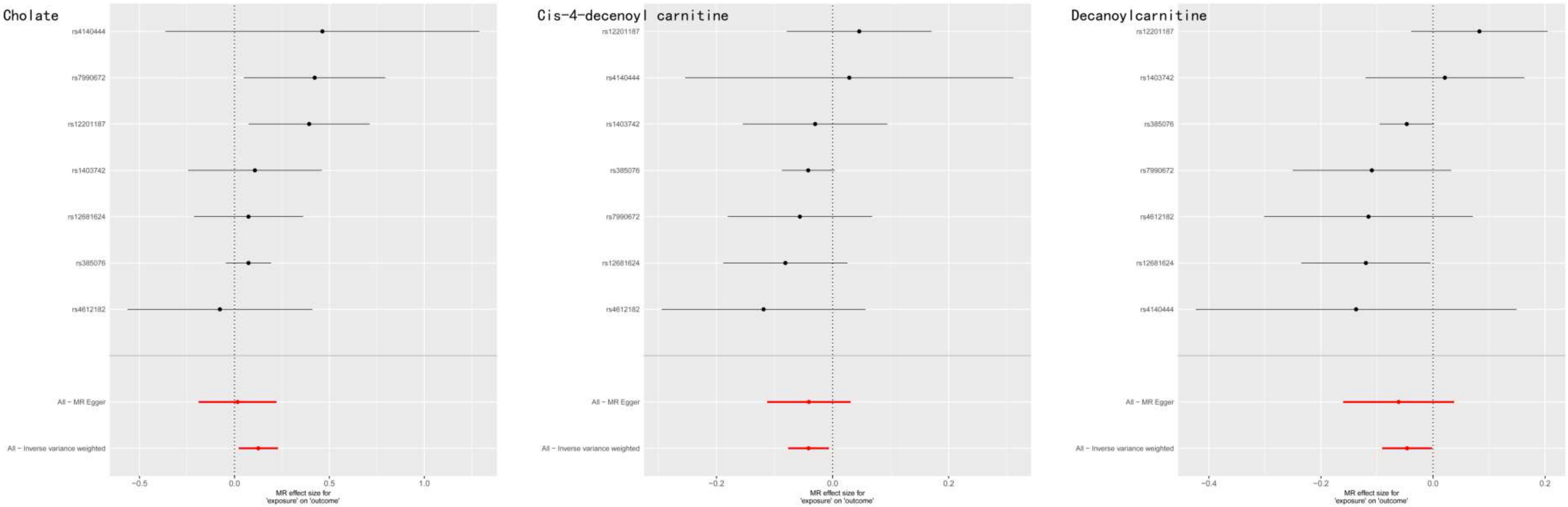

Cholate
rs4140444
rs7990672
rs12201187
rs1403742
rs12681624
rs385076
rs4612182
All − MR Egger
All − Inverse variance weighted
−0.5
0.0
0.5
1.0
MR effect size for
'exposure' on 'outcome'
Cis-4-decenoyl carnitine
rs12201187
rs4140444
rs1403742
rs385076
rs7990672
rs12681624
rs4612182
All − MR Egger
All − Inverse variance weighted
−0.2
0.0
0.2
MR effect size for
'exposure' on 'outcome'
Decanoylcarnitine
rs12201187
rs1403742
rs385076
rs7990672
rs4612182
rs12681624
rs4140444
All − MR Egger
All − Inverse variance weighted
−0.4
−0.2
0.0
0.2
MR effect size for
'exposure' on 'outcome'

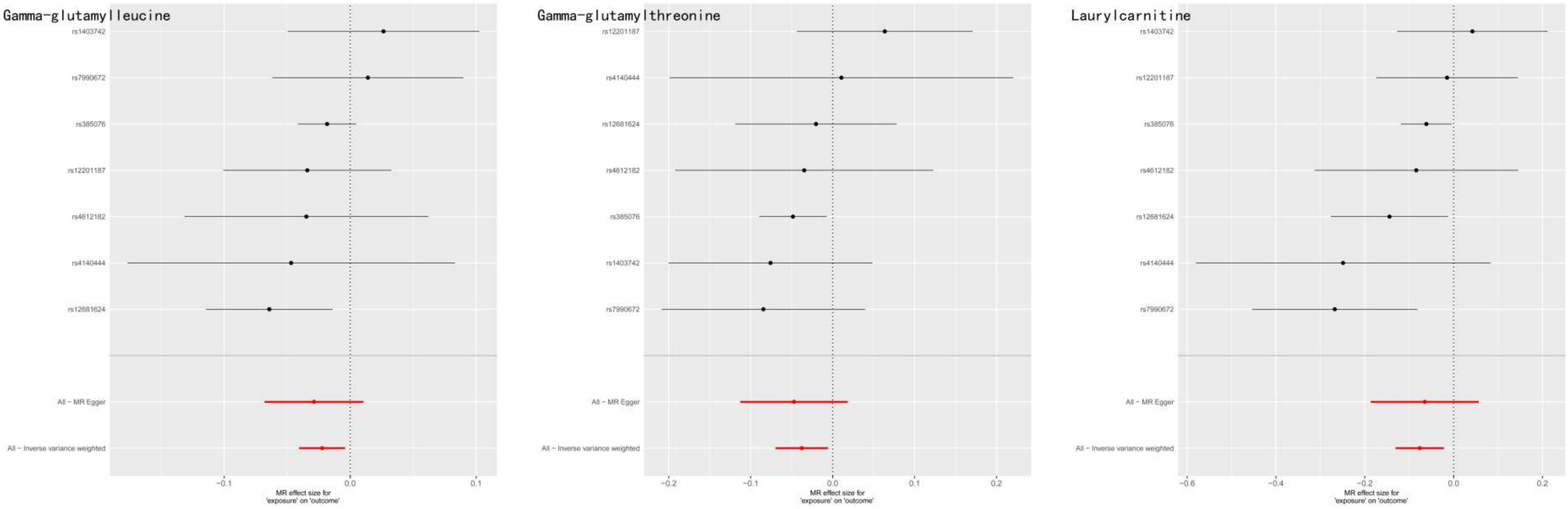
Gamma-glutamylleucine
rs1403742
rs7990672
rs385076
rs12201187
rs4612182
rs4140444
rs12681624
All − MR Egger
All − Inverse variance weighted
−0.1
0.0
0.1
MR effect size for
'exposure' on 'outcome'
Gamma-glutamylthreonine
rs12201187
rs4140444
rs12681624
rs4612182
rs385076
rs1403742
rs7990672
All − MR Egger
All − Inverse variance weighted
−0.2
−0.1
0.0
0.1
0.2
MR effect size for
'exposure' on 'outcome'
Laurylcarnitine
rs1403742
rs12201187
rs385076
rs4612182
rs12681624
rs4140444
rs7990672
All − MR Egger
All − Inverse variance weighted
−0.6
−0.4
−0.2
0.0
0.2
MR effect size for
'exposure' on 'outcome'

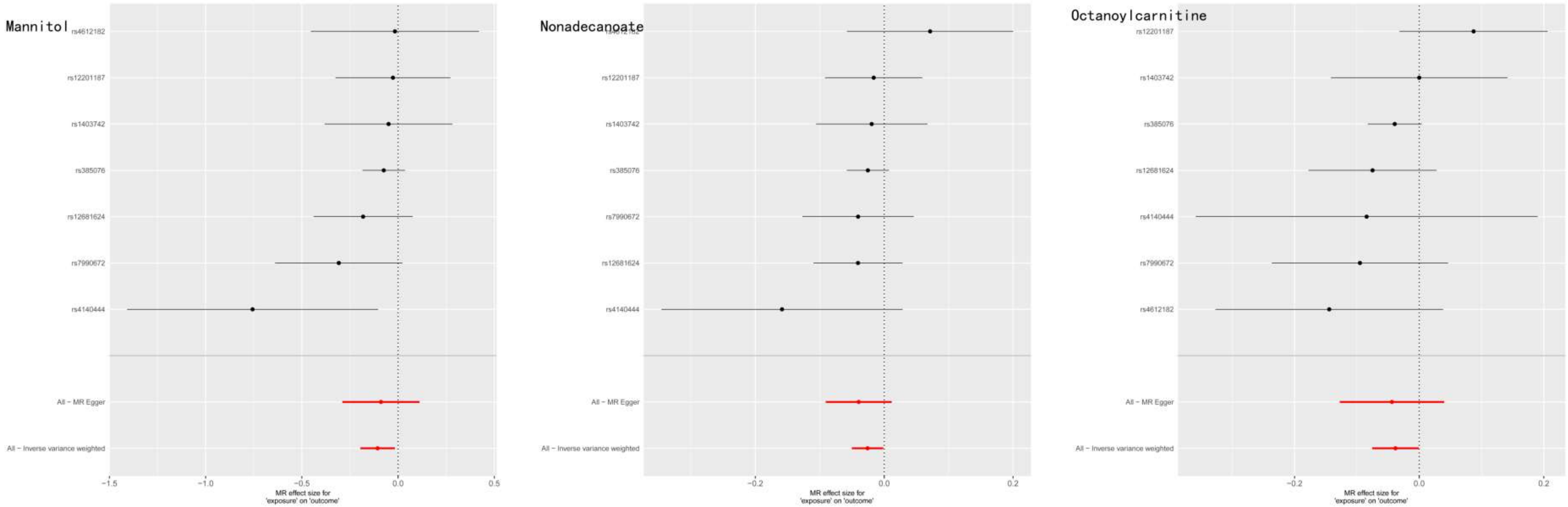

Mannitol
rs4612182
rs12201187
rs1403742
rs385076
rs12681624
rs7990672
rs4140444
All − MR Egger
All − Inverse variance weighted
−1.5
−1.0
−0.5
0.0
0.5
MR effect size for
'exposure' on 'outcome'
Nonadecanoate
rs12201187
rs1403742
rs385076
rs7990672
rs12681624
rs4140444
All − MR Egger
All − Inverse variance weighted
−0.2
0.0
0.2
MR effect size for
'exposure' on 'outcome'
Octanoylcarnitine
rs12201187
rs1403742
rs385076
rs12681624
rs4140444
rs7990672
rs4612182
All − MR Egger
All − Inverse variance weighted
−0.2
0.0
0.2
MR effect size for
'exposure' on 'outcome'

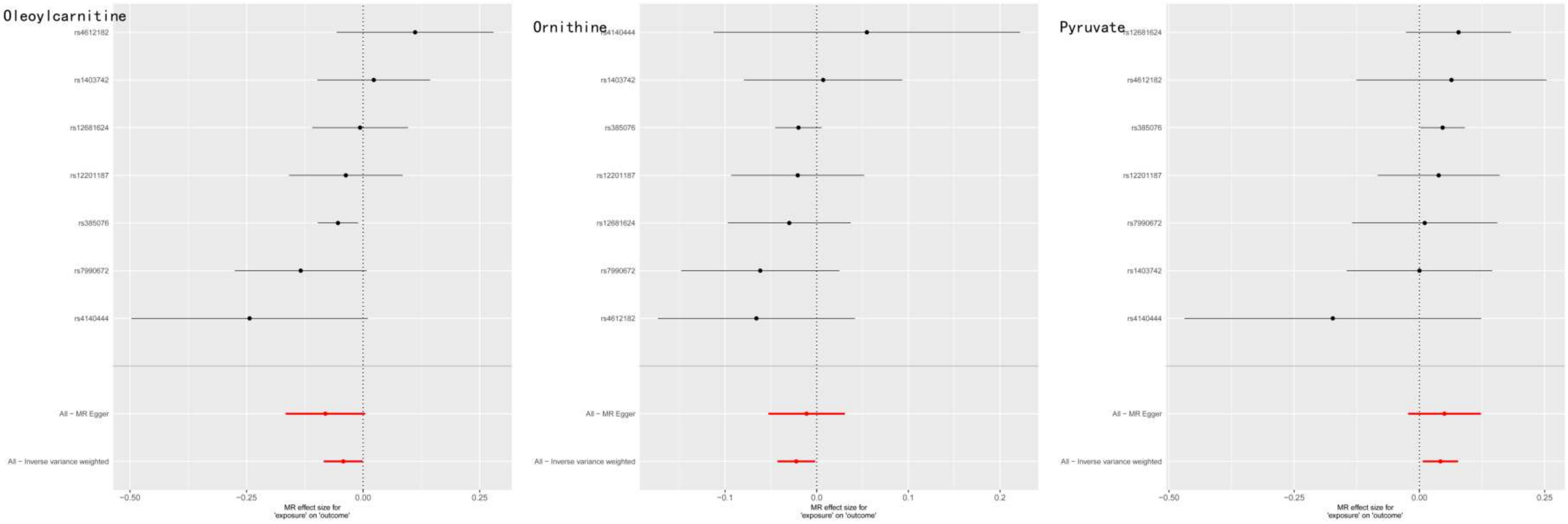

Oleoylcarnitine
rs4612182
rs1403742
rs12681624
rs12201187
rs385076
rs7990672
rs4140444
All − MR Egger
All − Inverse variance weighted
−0.50
−0.25
0.00
0.25
MR effect size for
'exposure' on 'outcome'
Ornithine
rs4140444
rs1403742
rs385076
rs12201187
rs12681624
rs7990672
rs4612182
All − MR Egger
All − Inverse variance weighted
−0.1
0.0
0.1
0.2
MR effect size for
'exposure' on 'outcome'
Pyruvate
rs12681624
rs4612182
rs385076
rs12201187
rs7990672
rs1403742
rs4140444
All − MR Egger
All − Inverse variance weighted
−0.50
−0.25
0.00
0.25
MR effect size for
'exposure' on 'outcome'

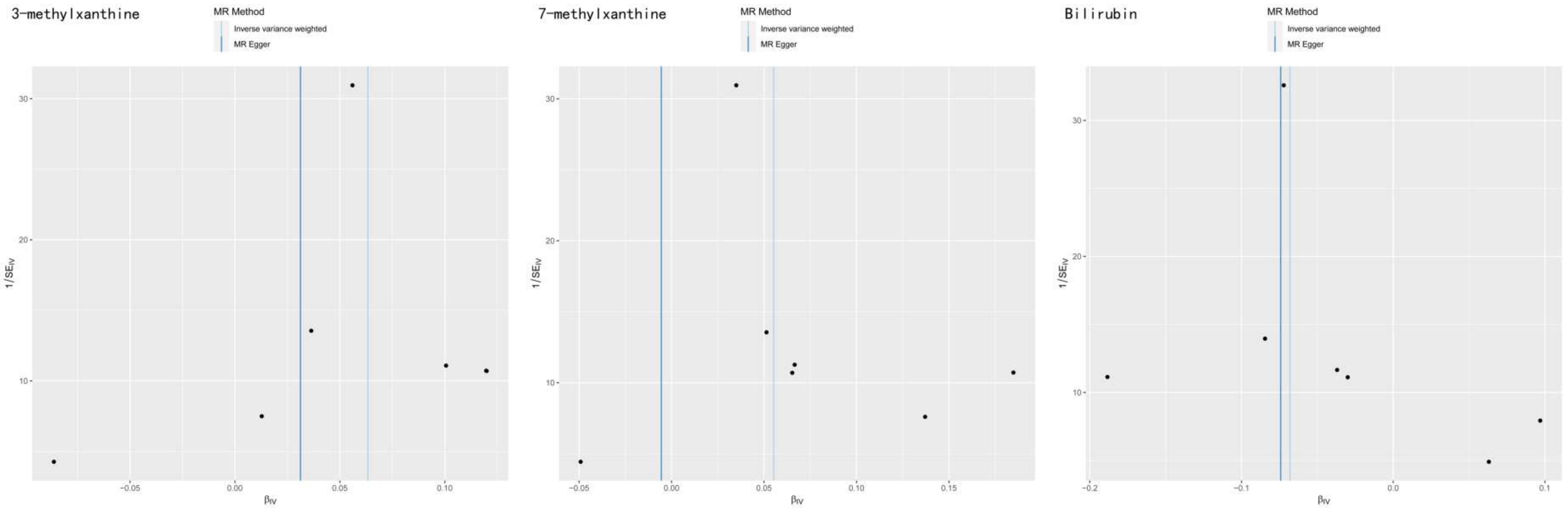
3-methylxanthine
MR Method
Inverse variance weighted
MR Egger
1/SEIV
βIV
7-methylxanthine
Bilirubin
B.

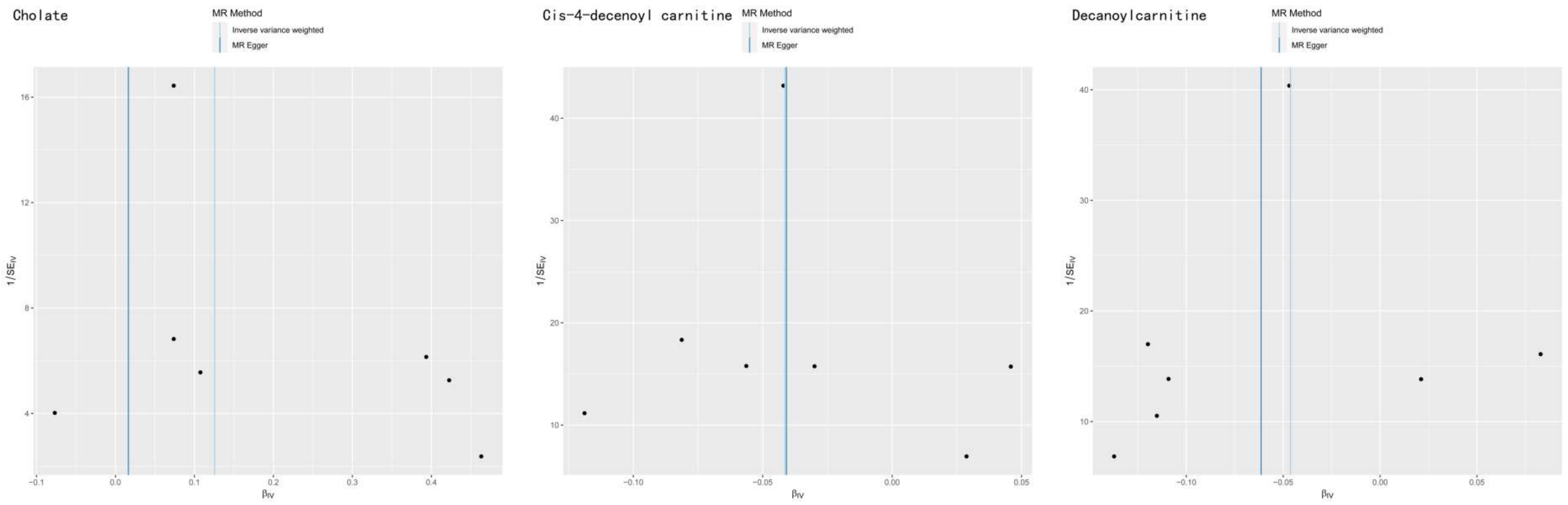
Cholate
MR Method
Inverse variance weighted
MR Egger
1/SE_IV
β_IV
Cis-4-decenoyl carnitine
MR Method
Inverse variance weighted
MR Egger
1/SE_IV
β_IV
Decanoylcarnitine
MR Method
Inverse variance weighted
MR Egger
1/SE_IV
β_IV

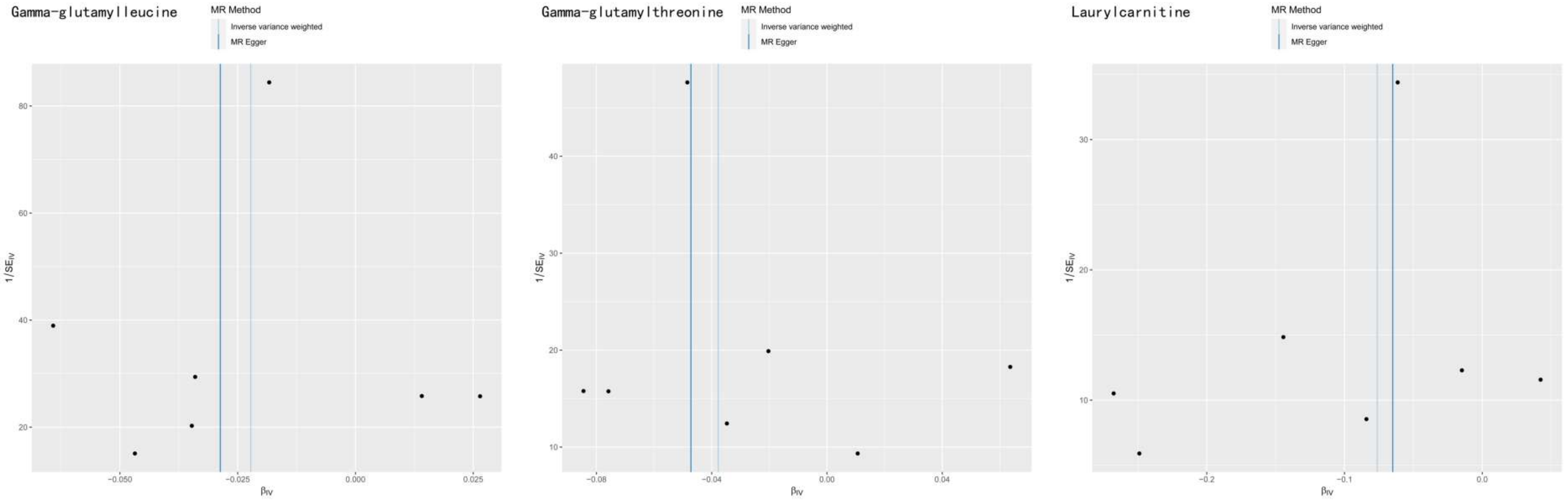
Gamma-glutamylleucine
MR Method
Inverse variance weighted
MR Egger
1/SE_IV
β_IV
Gamma-glutamylthreonine
MR Method
Inverse variance weighted
MR Egger
1/SE_IV
β_IV
Laurylcarnitine
MR Method
Inverse variance weighted
MR Egger
1/SE_IV
β_IV

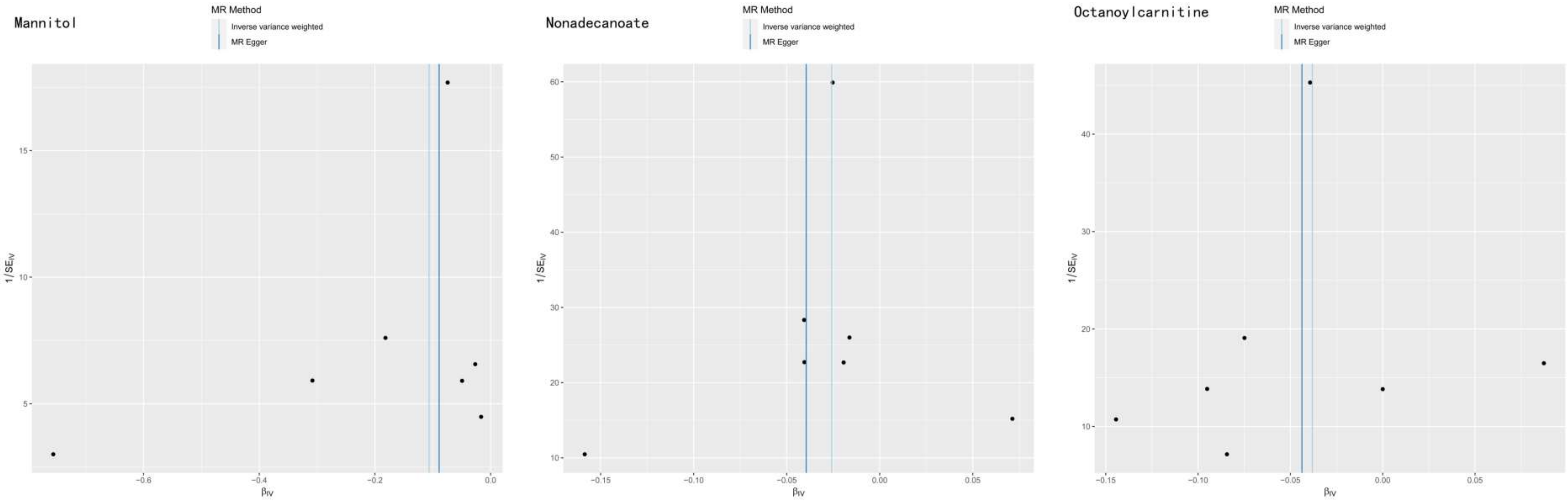
Mannitol
MR Method
Inverse variance weighted
MR Egger
1/SEIV
βIV
−0.6
−0.4
−0.2
0.0
5
10
15
Nonadecanoate
MR Method
Inverse variance weighted
MR Egger
1/SEIV
βIV
−0.15
−0.10
−0.05
0.00
0.05
10
20
30
40
50
60
Octanoylcarnitine
MR Method
Inverse variance weighted
MR Egger
1/SEIV
βIV
−0.15
−0.10
−0.05
0.00
0.05
10
20
30
40

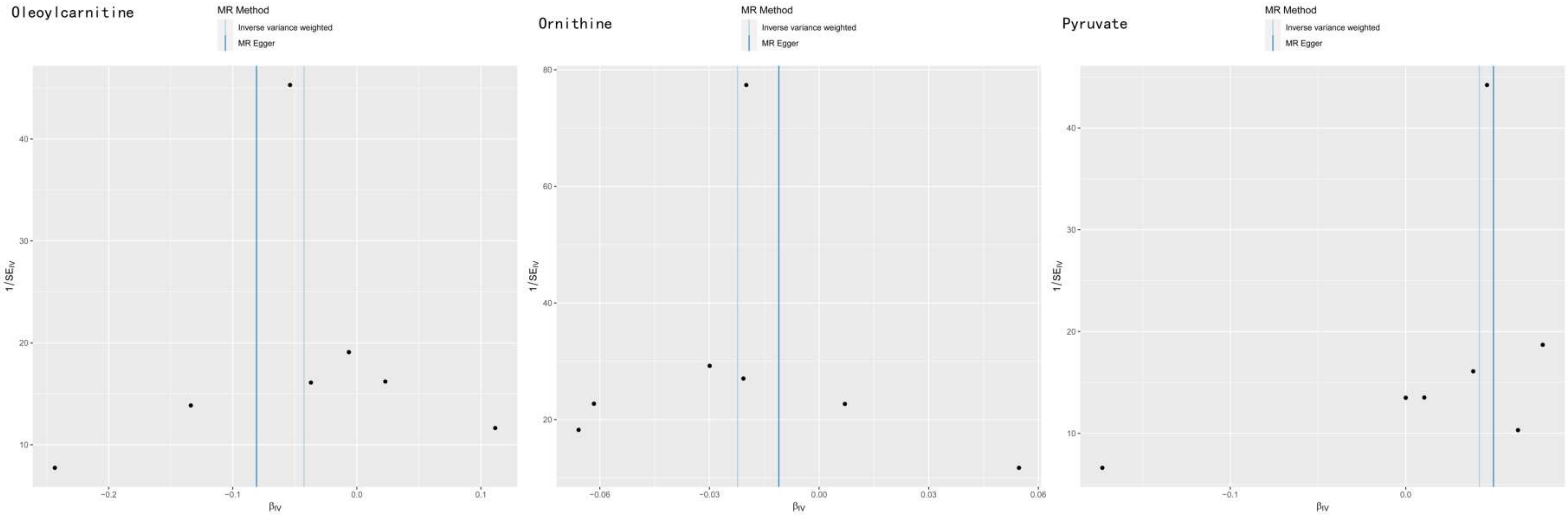

Oleoylcarnitine
MR Method
Inverse variance weighted
MR Egger
1/SE_IV
β_IV
Ornithine
MR Method
Inverse variance weighted
MR Egger
1/SE_IV
β_IV
Pyruvate
MR Method
Inverse variance weighted
MR Egger
1/SE_IV
β_IV

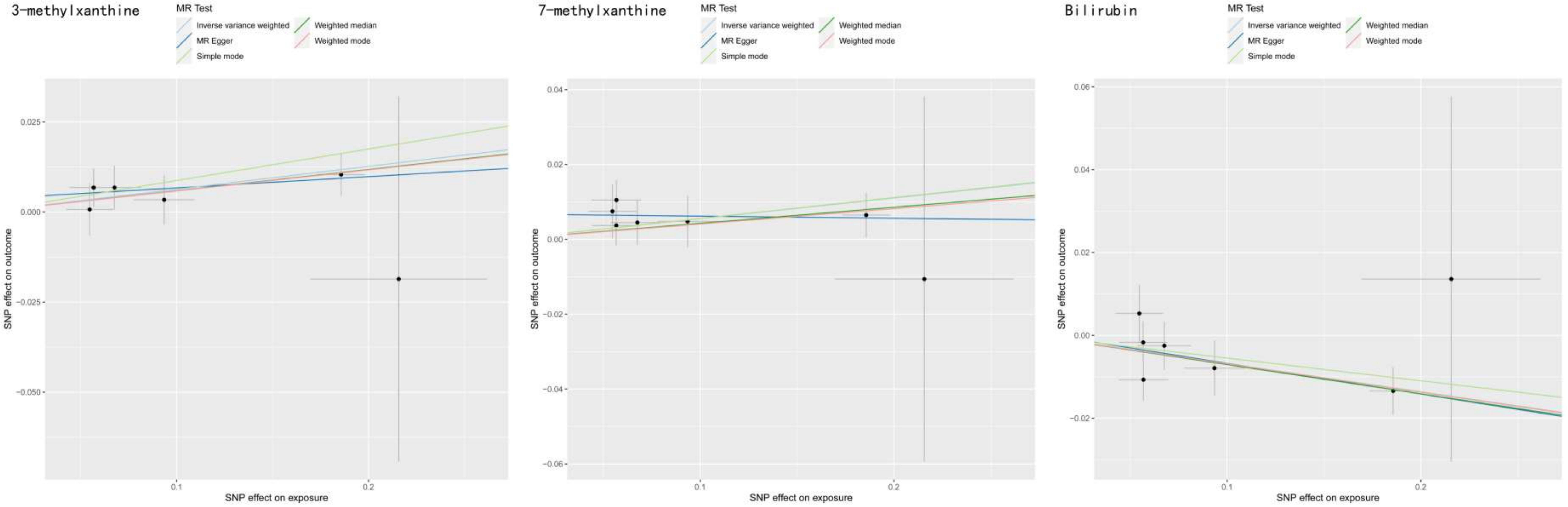
3-methylxanthine
MR Test
Inverse variance weighted
MR Egger
Simple mode
Weighted median
Weighted mode
0.025
0.000
−0.025
−0.050
SNP effect on outcome
0.1
0.2
SNP effect on exposure
7-methylxanthine
MR Test
Inverse variance weighted
MR Egger
Simple mode
Weighted median
Weighted mode
0.04
0.02
0.00
−0.02
−0.04
−0.06
SNP effect on outcome
0.1
0.2
SNP effect on exposure
Bilirubin
MR Test
Inverse variance weighted
MR Egger
Simple mode
Weighted median
Weighted mode
0.06
0.04
0.02
0.00
−0.02
SNP effect on outcome
0.1
0.2
SNP effect on exposure

c.

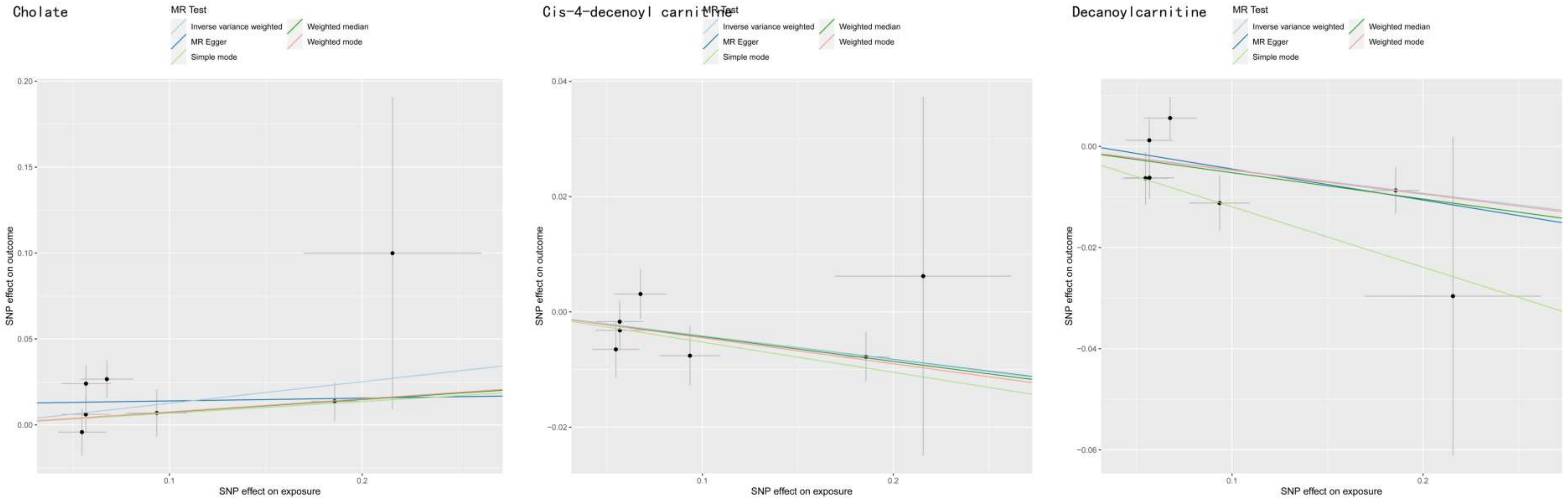

Cholate
MR Test
Inverse variance weighted
MR Egger
Simple mode
Weighted median
Weighted mode
0.20
0.15
0.10
0.05
0.00
0.1
0.2
SNP effect on outcome
SNP effect on exposure
Cis-4-decenoyl carnitine
0.04
0.02
0.00
-0.02
Decanoylcarnitine
-0.02
-0.04
-0.06

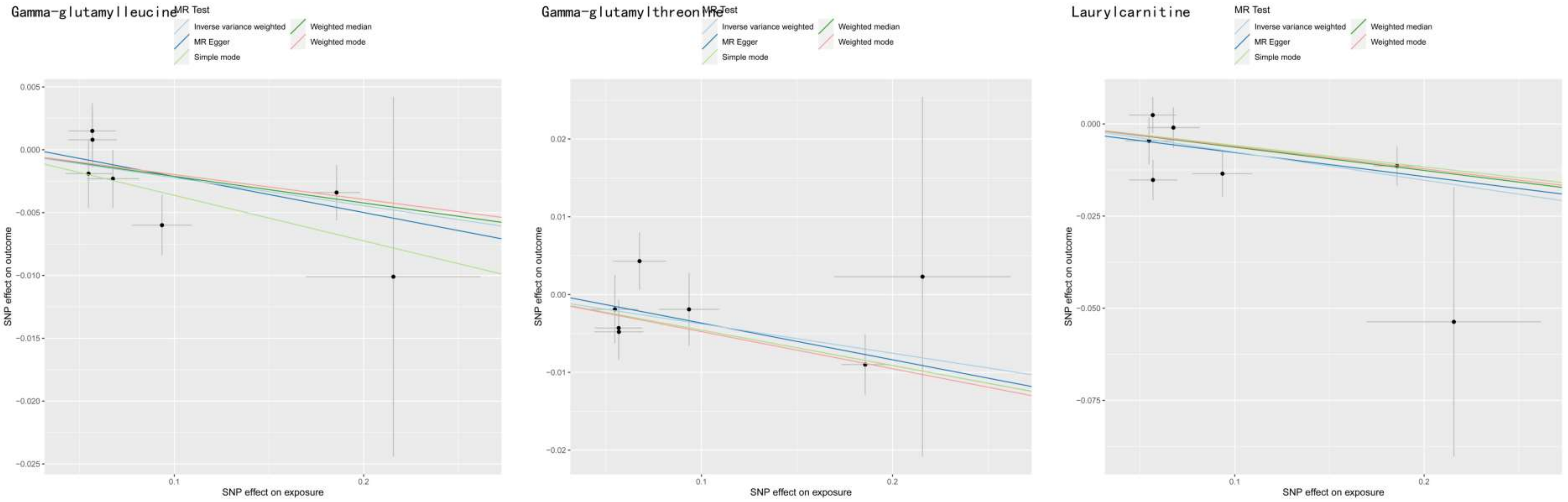

Gamma-glutamylleucine
MR Test
Inverse variance weighted
MR Egger
Simple mode
Weighted median
Weighted mode
SNP effect on outcome
SNP effect on exposure
0.005
0.000
−0.005
−0.010
−0.015
−0.020
−0.025
0.1
0.2
Gamma-glutamylthreonine
MR Test
Inverse variance weighted
MR Egger
Simple mode
Weighted median
Weighted mode
SNP effect on outcome
SNP effect on exposure
0.02
0.01
0.00
−0.01
−0.02
0.1
0.2
Laurylcarnitine
MR Test
Inverse variance weighted
MR Egger
Simple mode
Weighted median
Weighted mode
SNP effect on outcome
SNP effect on exposure
0.000
−0.025
−0.050
−0.075
0.1
0.2

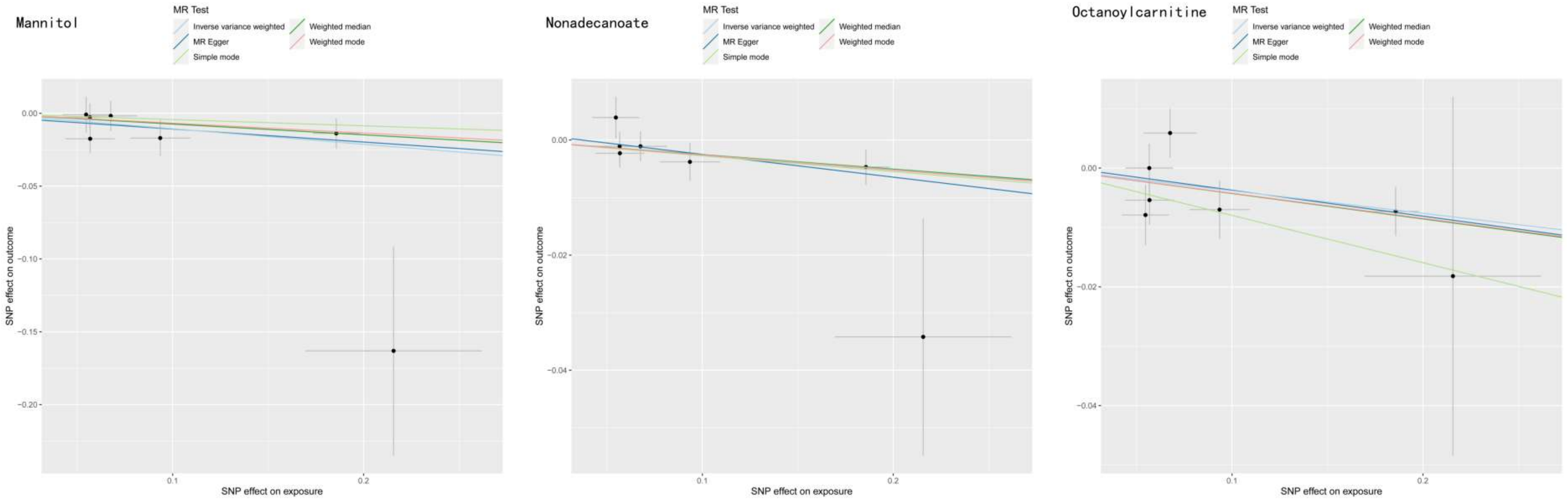

Mannitol
Nonadecanoate
Octanoylcarnitine
MR Test
Inverse variance weighted
MR Egger
Simple mode
Weighted median
Weighted mode
SNP effect on outcome
SNP effect on exposure
0.00
-0.05
-0.10
-0.15
-0.20
0.1
0.2
0.00
-0.02
-0.04
0.00
-0.02
-0.04

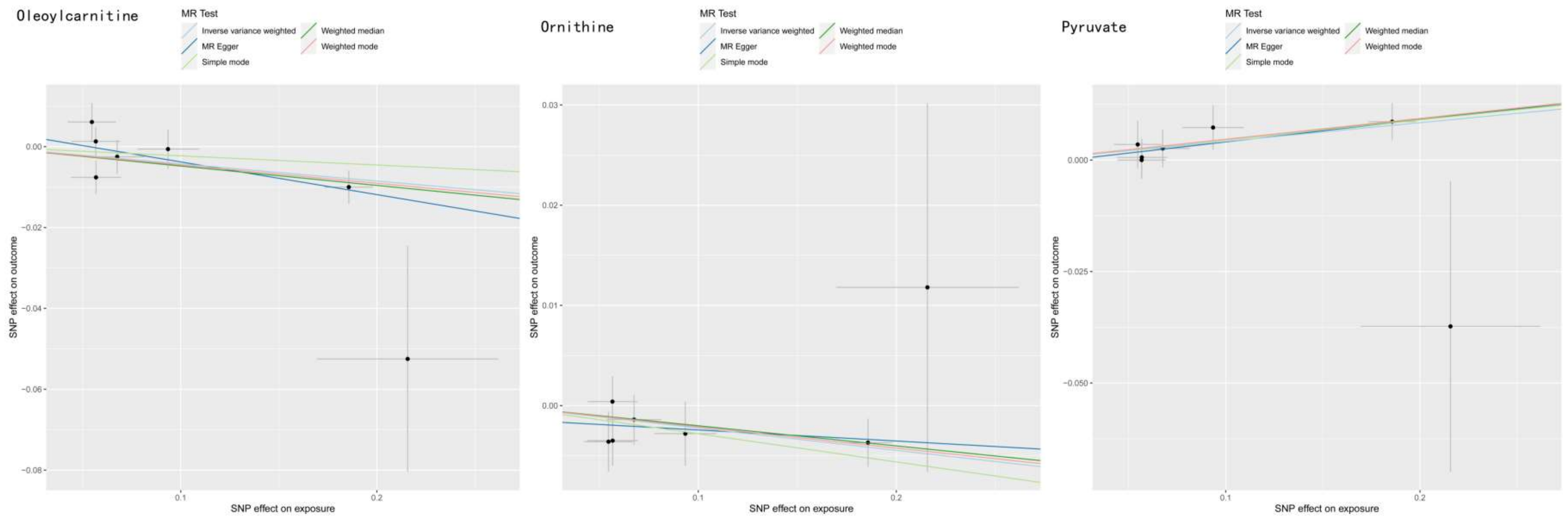
Oleoylcarnitine
MR Test
Inverse variance weighted
MR Egger
Simple mode
Weighted median
Weighted mode
0.00
−0.02
−0.04
−0.06
−0.08
SNP effect on outcome
0.1
0.2
SNP effect on exposure
Ornithine
MR Test
Inverse variance weighted
MR Egger
Simple mode
Weighted median
Weighted mode
0.03
0.02
0.01
0.00
SNP effect on outcome
0.1
0.2
SNP effect on exposure
Pyruvate
MR Test
Inverse variance weighted
MR Egger
Simple mode
Weighted median
Weighted mode
0.000
−0.025
−0.050
SNP effect on outcome
0.1
0.2
SNP effect on exposure

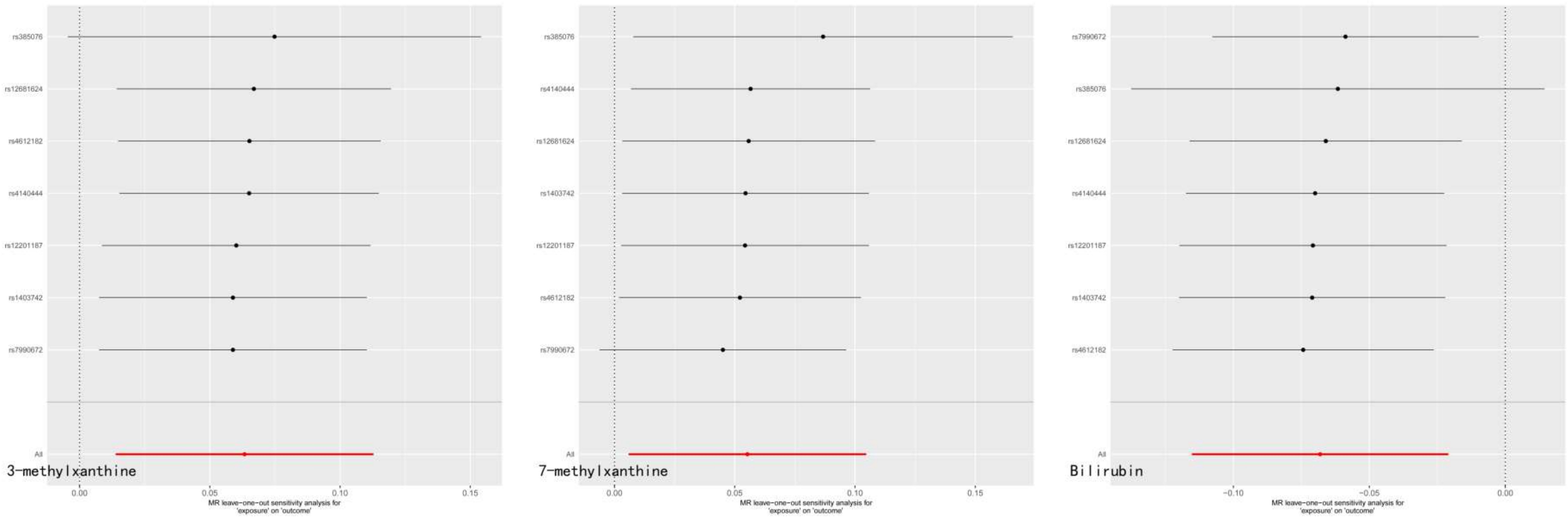
rs385076
rs12681624
rs4612182
rs4140444
rs12201187
rs1403742
rs7990672
All
3-methylxanthine
0.00
0.05
0.10
0.15
MR leave-one-out sensitivity analysis for
'exposure' on 'outcome'
rs385076
rs4140444
rs12681624
rs1403742
rs12201187
rs4612182
rs7990672
All
7-methylxanthine
0.00
0.05
0.10
0.15
MR leave-one-out sensitivity analysis for
'exposure' on 'outcome'
rs7990672
rs385076
rs12681624
rs4140444
rs12201187
rs1403742
rs4612182
All
Bilirubin
−0.10
−0.05
0.00
MR leave-one-out sensitivity analysis for
'exposure' on 'outcome'

D.

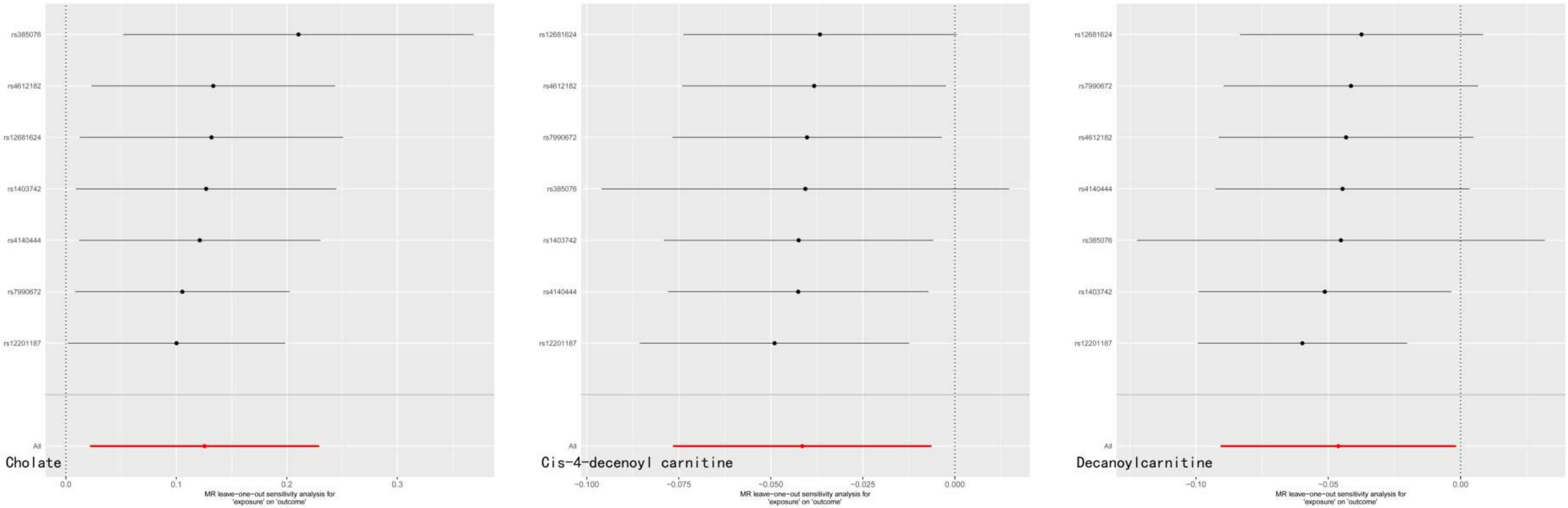
Cholate
rs385076
rs4612182
rs12681624
rs1403742
rs4140444
rs7990672
rs12201187
All
0.0
0.1
0.2
0.3
MR leave−one−out sensitivity analysis for 'exposure' on 'outcome'
Cis-4-decenoyl carnitine
rs12681624
rs4612182
rs7990672
rs385076
rs1403742
rs4140444
rs12201187
All
−0.100
−0.075
−0.050
−0.025
0.000
MR leave−one−out sensitivity analysis for 'exposure' on 'outcome'
Decanoylcarnitine
rs12681624
rs7990672
rs4612182
rs4140444
rs385076
rs1403742
rs12201187
All
−0.10
−0.05
0.00
MR leave−one−out sensitivity analysis for 'exposure' on 'outcome'

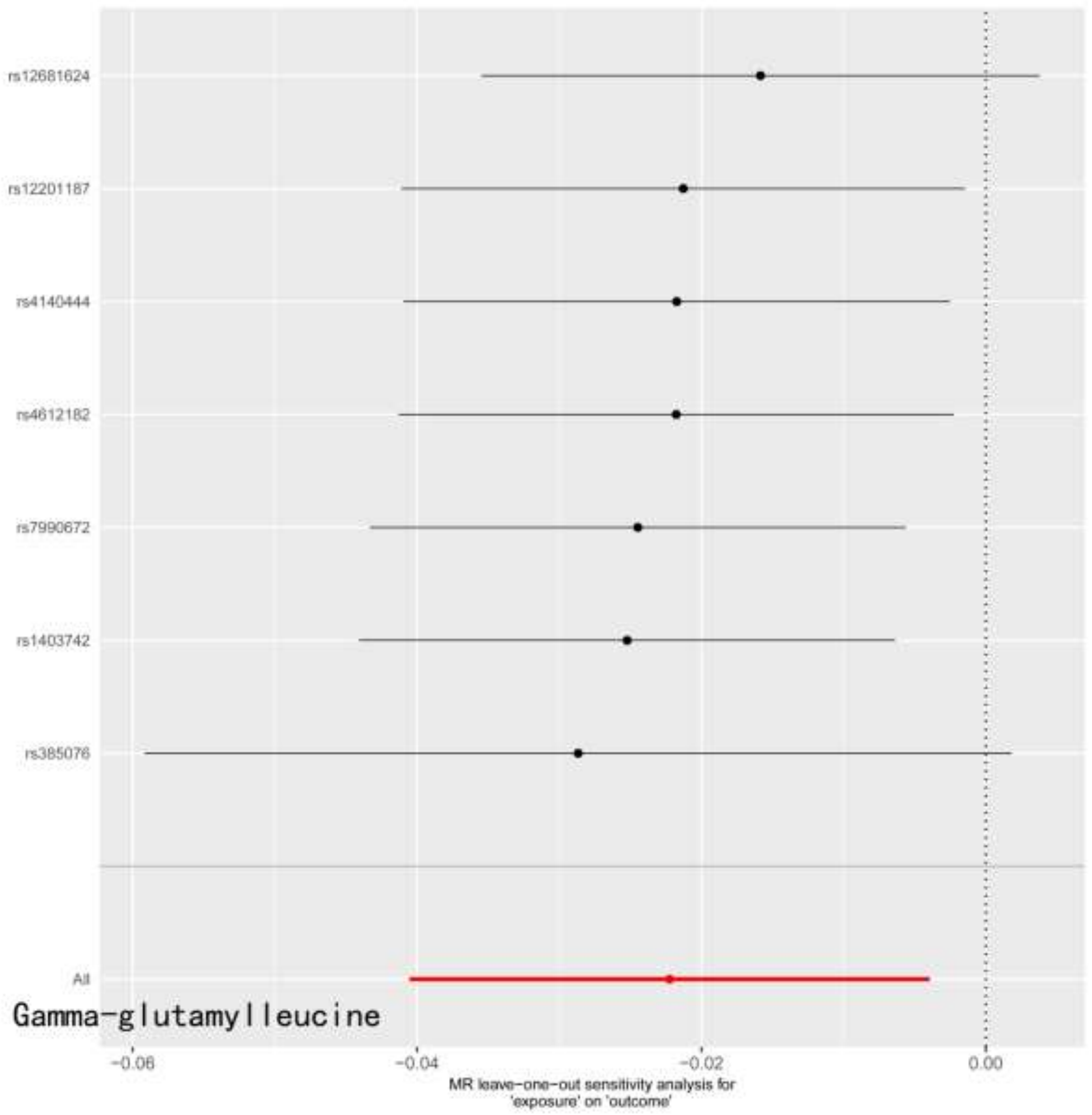
rs12681624
rs12201187
rs4140444
rs4612182
rs7990672
rs1403742
rs385076
All
Gamma-glutamylleucine
−0.06
−0.04
−0.02
0.00
MR leave−one−out sensitivity analysis for
'exposure' on 'outcome'

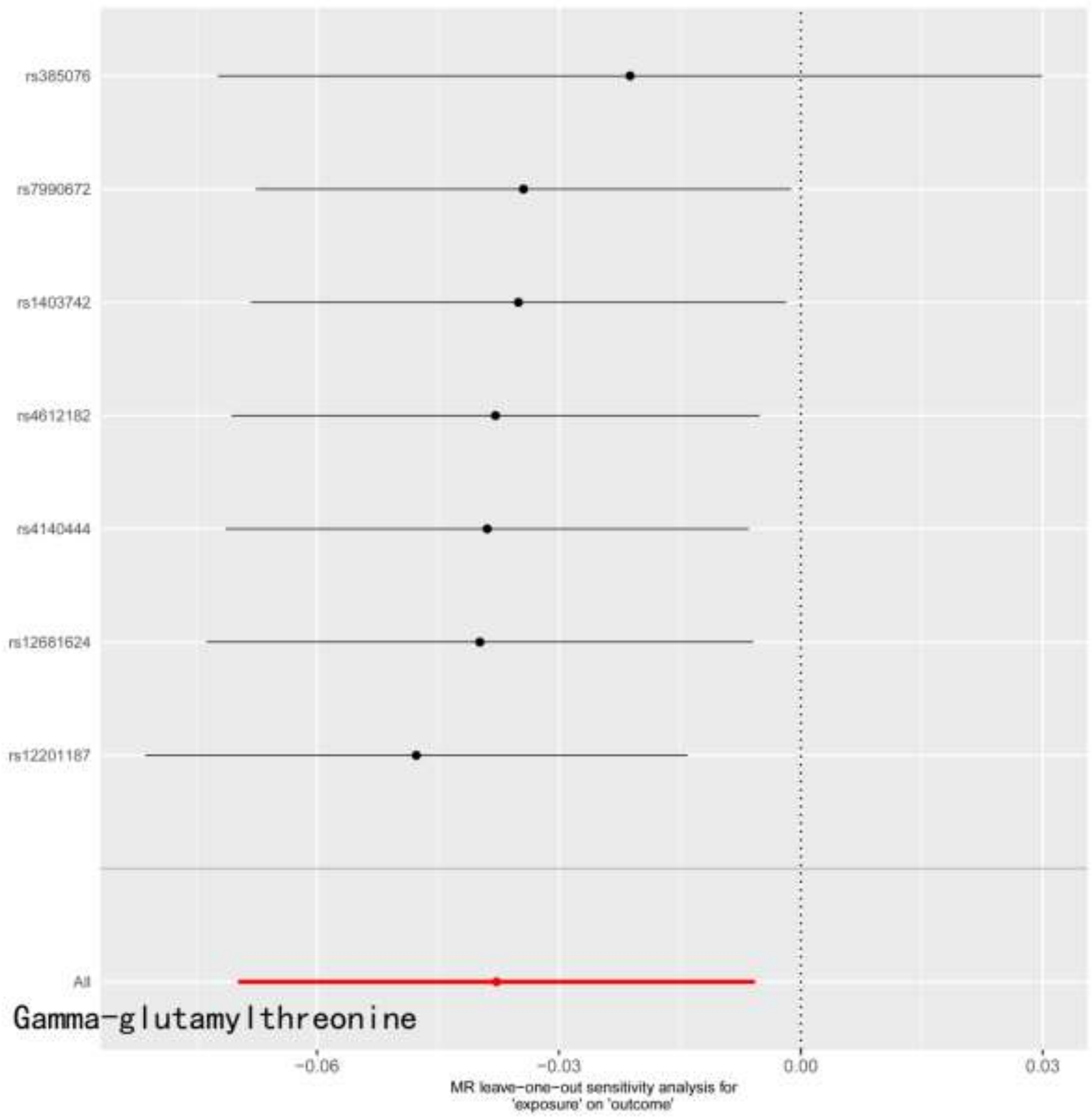
rs385076
rs7990672
rs1403742
rs4612182
rs4140444
rs12681624
rs12201187
All
Gamma-glutamylthreonine
−0.06
−0.03
0.00
0.03
MR leave−one−out sensitivity analysis for
'exposure' on 'outcome'

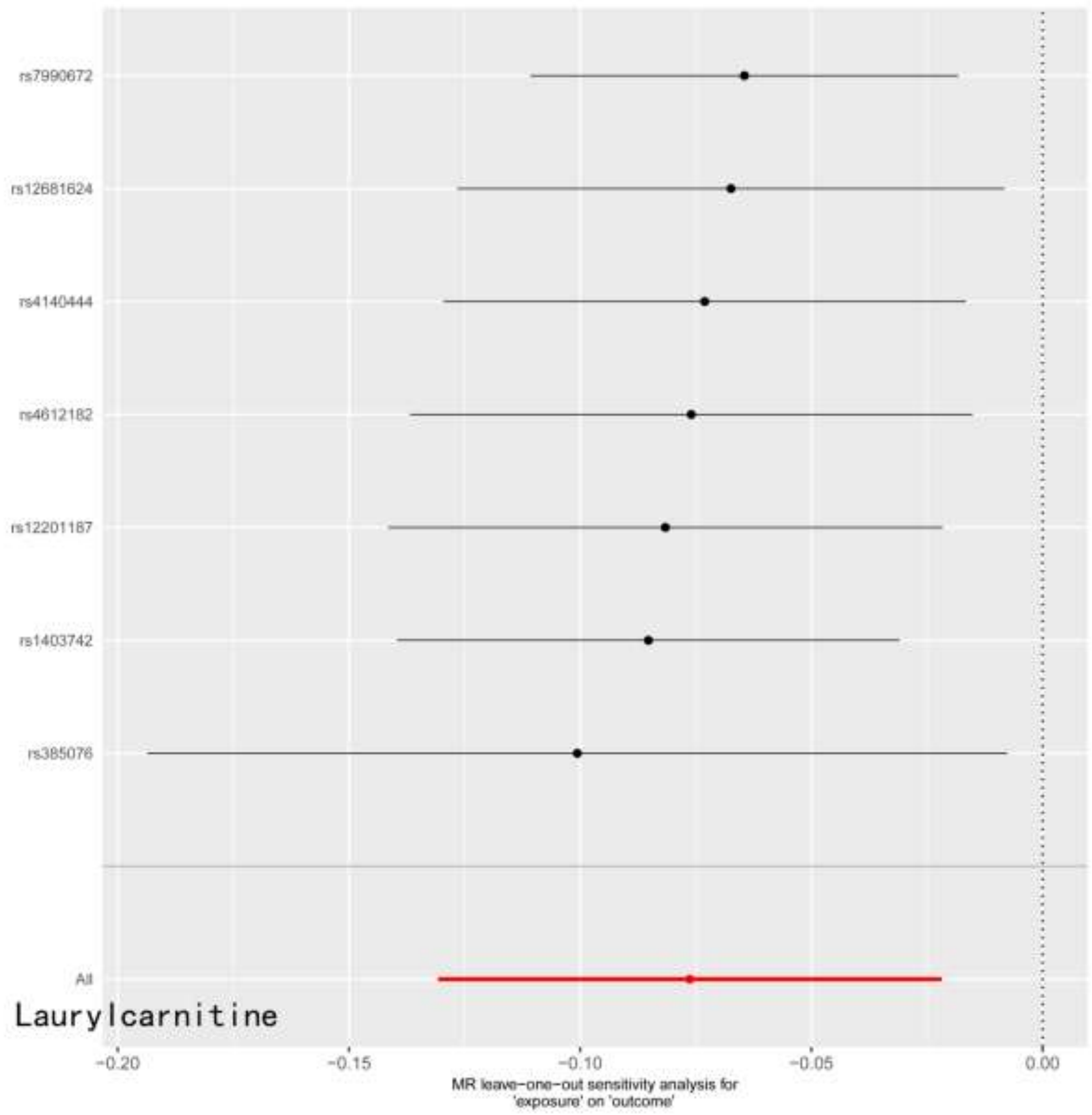
rs7990672
rs12681624
rs4140444
rs4612182
rs12201187
rs1403742
rs385076
All
Laurylcarnitine
−0.20
−0.15
−0.10
−0.05
0.00
MR leave−one−out sensitivity analysis for
'exposure' on 'outcome'

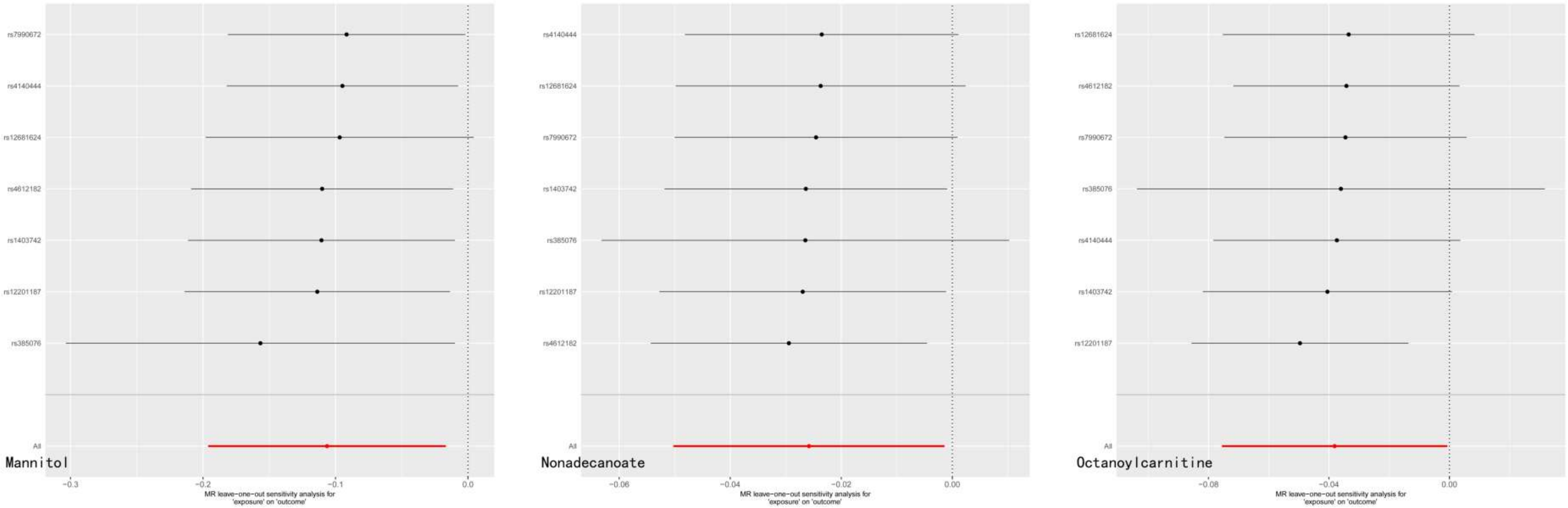
Mannitol
rs7990672
rs4140444
rs12681624
rs4612182
rs1403742
rs12201187
rs385076
All
−0.3
−0.2
−0.1
0.0
MR leave−one−out sensitivity analysis for 'exposure' on 'outcome'
Nonadecanoate
rs4140444
rs12681624
rs7990672
rs1403742
rs385076
rs12201187
rs4612182
All
−0.06
−0.04
−0.02
0.00
MR leave−one−out sensitivity analysis for 'exposure' on 'outcome'
Octanoylcarnitine
rs12681624
rs4612182
rs7990672
rs385076
rs4140444
rs1403742
rs12201187
All
−0.08
−0.04
0.00
MR leave−one−out sensitivity analysis for 'exposure' on 'outcome'

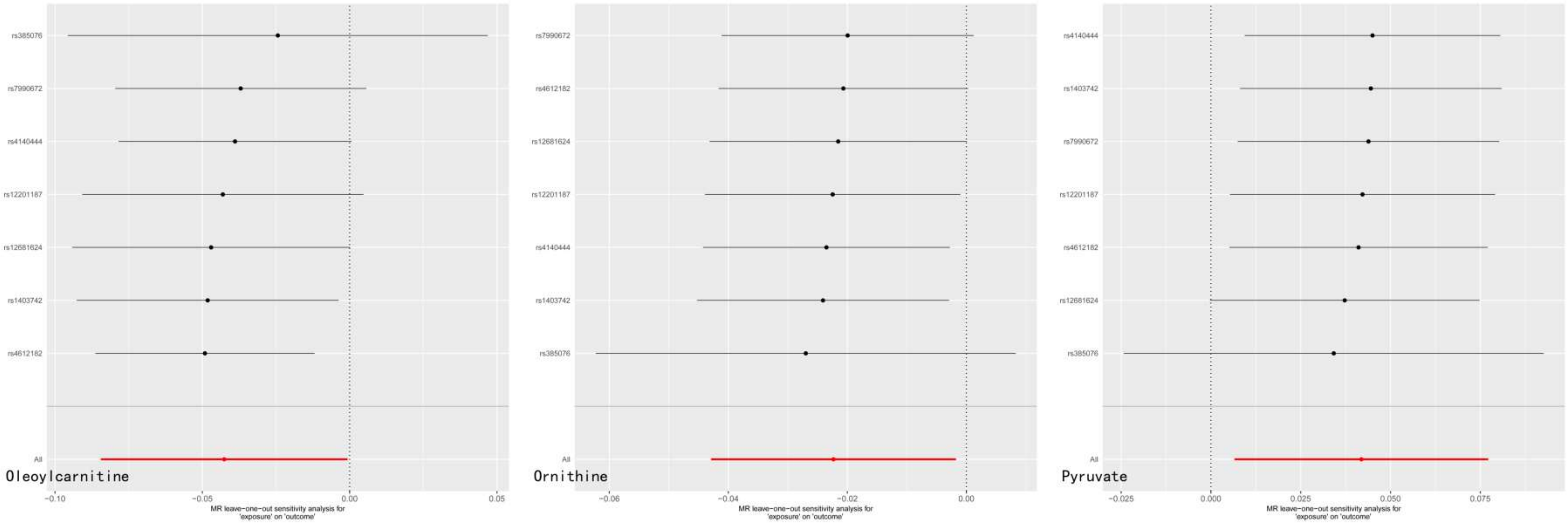

Oleoylcarnitine
rs385076
rs7990672
rs4140444
rs12201187
rs12681624
rs1403742
rs4612182
All
−0.10
−0.05
0.00
0.05
MR leave−one−out sensitivity analysis for
'exposure' on 'outcome'
Ornithine
rs7990672
rs4612182
rs12681624
rs12201187
rs4140444
rs1403742
rs385076
All
−0.06
−0.04
−0.02
0.00
MR leave−one−out sensitivity analysis for
'exposure' on 'outcome'
Pyruvate
rs4140444
rs1403742
rs7990672
rs12201187
rs4612182
rs12681624
rs385076
All
−0.025
0.000
0.025
0.050
0.075
MR leave−one−out sensitivity analysis for
'exposure' on 'outcome'

S4. Outcome plot of PD-L1 versus blood metabolites. (A), forest plot (B), funnel plot (C), scatter plot (D), leave-one-out plot

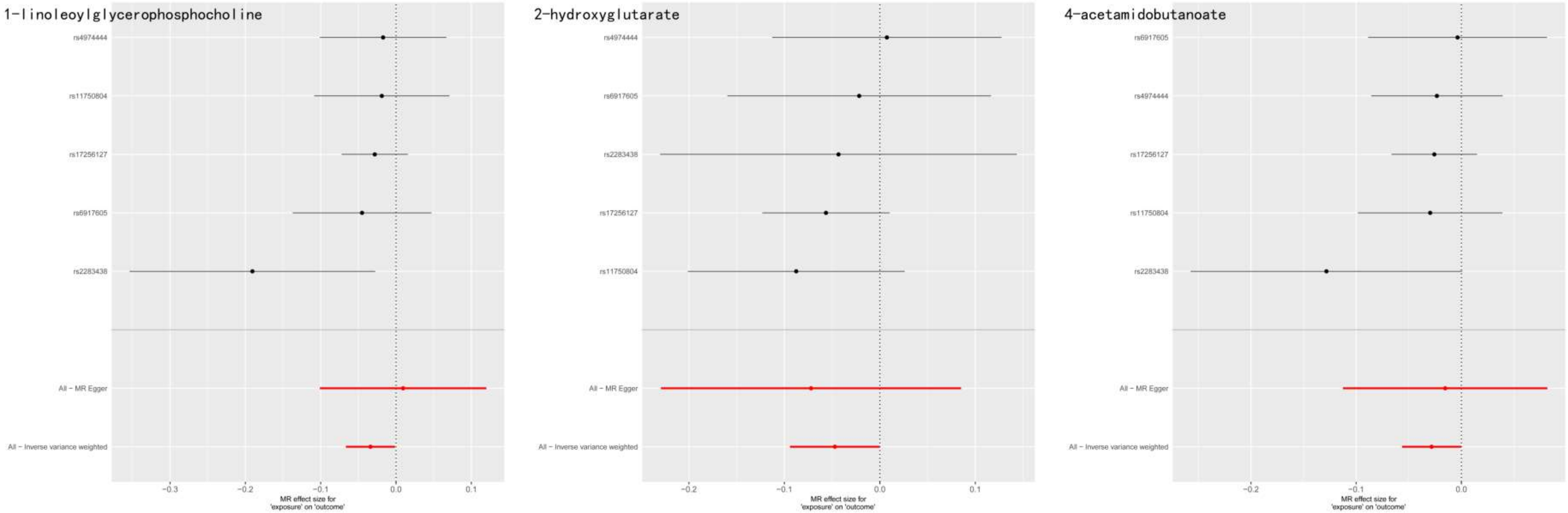

A.

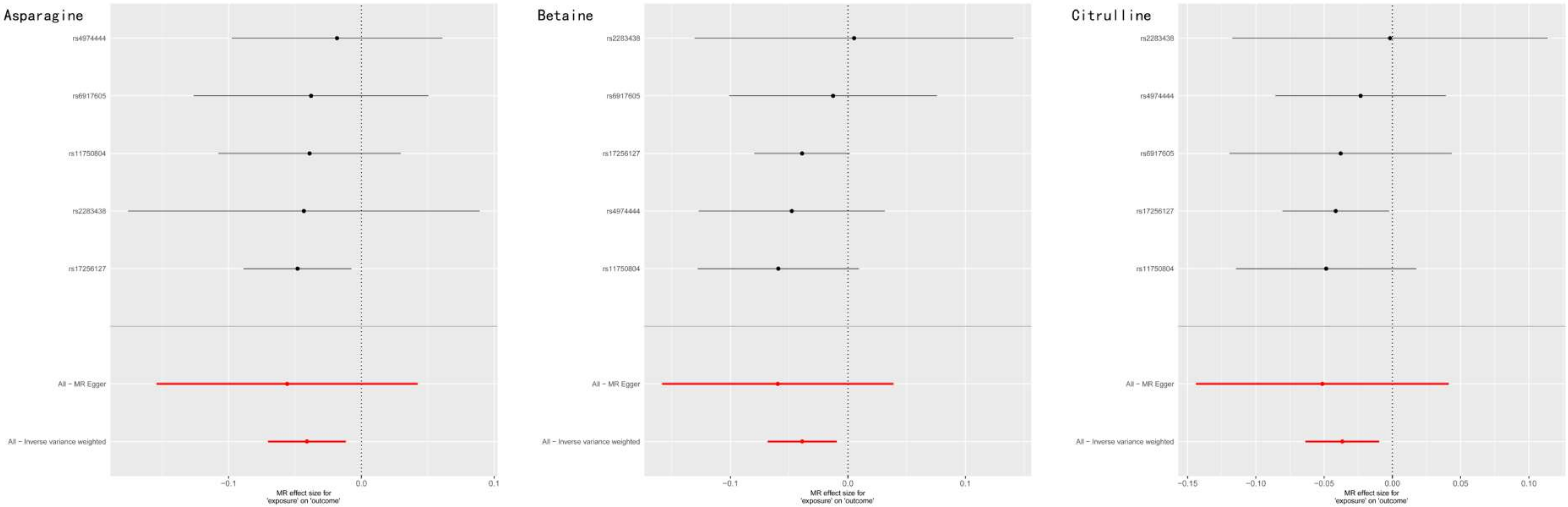
Asparagine
rs4974444
rs6917605
rs11750804
rs2283438
rs17256127
All − MR Egger
All − Inverse variance weighted
−0.1
0.0
0.1
MR effect size for
'exposure' on 'outcome'
Betaine
rs2283438
rs6917605
rs17256127
rs4974444
rs11750804
All − MR Egger
All − Inverse variance weighted
−0.1
0.0
0.1
MR effect size for
'exposure' on 'outcome'
Citrulline
rs2283438
rs4974444
rs6917605
rs17256127
rs11750804
All − MR Egger
All − Inverse variance weighted
−0.15
−0.10
−0.05
0.00
0.05
0.10
MR effect size for
'exposure' on 'outcome'

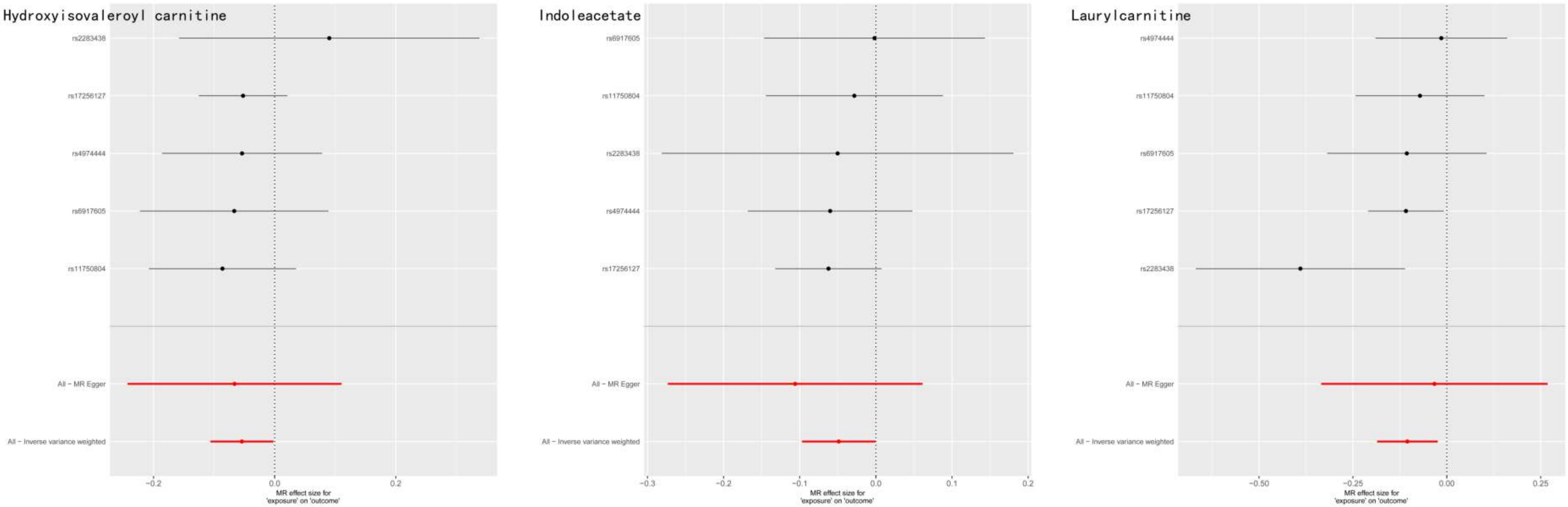

Hydroxyisovaleroyl carnitine
rs2283438
rs17256127
rs4974444
rs6917605
rs11750804
All − MR Egger
All − Inverse variance weighted
−0.2
0.0
0.2
MR effect size for
'exposure' on 'outcome'
Indoleacetate
rs6917605
rs11750804
rs2283438
rs4974444
rs17256127
All − MR Egger
All − Inverse variance weighted
−0.3
−0.2
−0.1
0.0
0.1
0.2
MR effect size for
'exposure' on 'outcome'
Laurylcarnitine
rs4974444
rs11750804
rs6917605
rs17256127
rs2283438
All − MR Egger
All − Inverse variance weighted
−0.50
−0.25
0.00
0.25
MR effect size for
'exposure' on 'outcome'

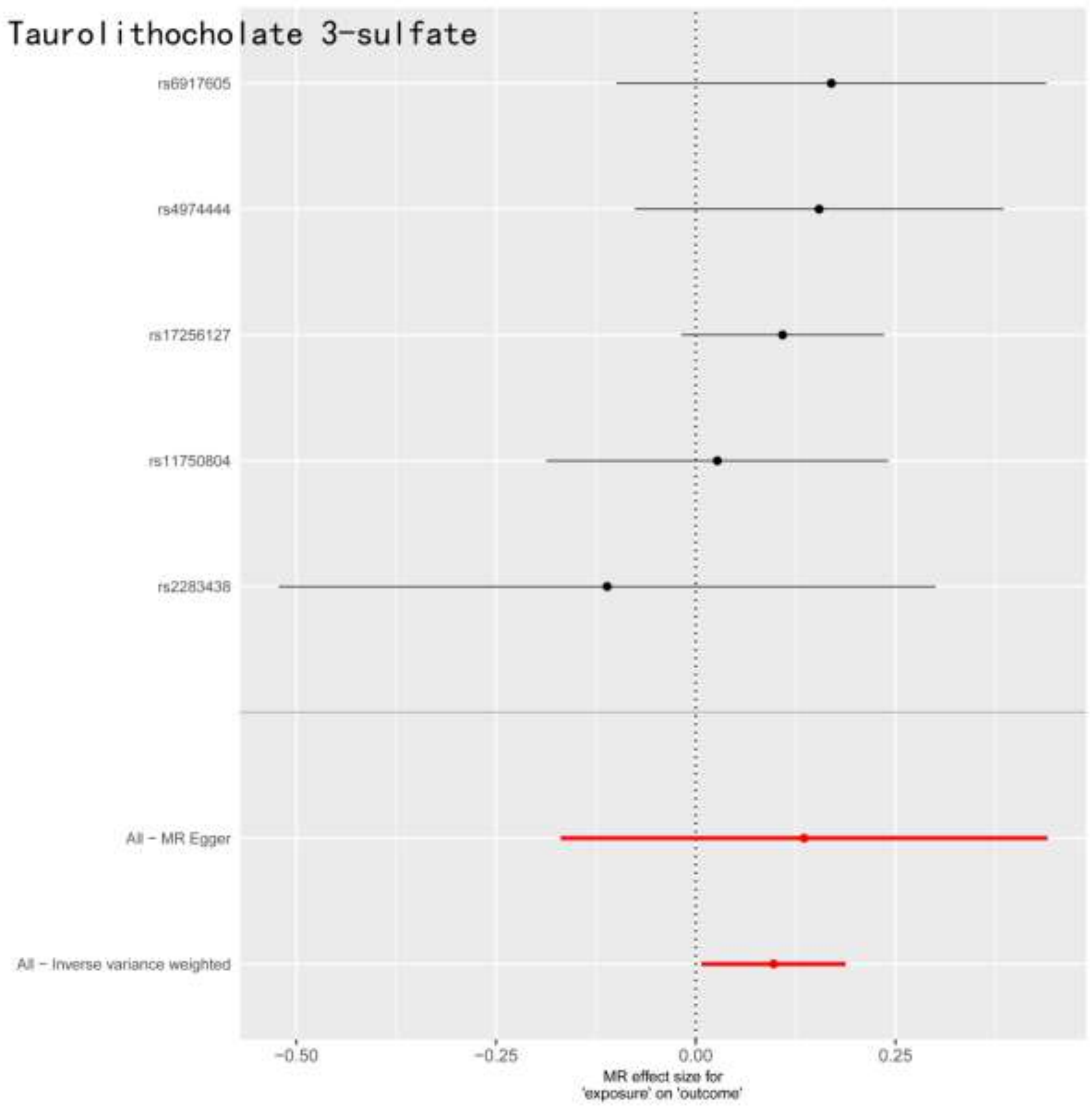

Taurolithocholate 3-sulfate
rs6917605
rs4974444
rs17256127
rs11750804
rs2283438
All − MR Egger
All − Inverse variance weighted
−0.50
−0.25
0.00
0.25
MR effect size for
'exposure' on 'outcome'

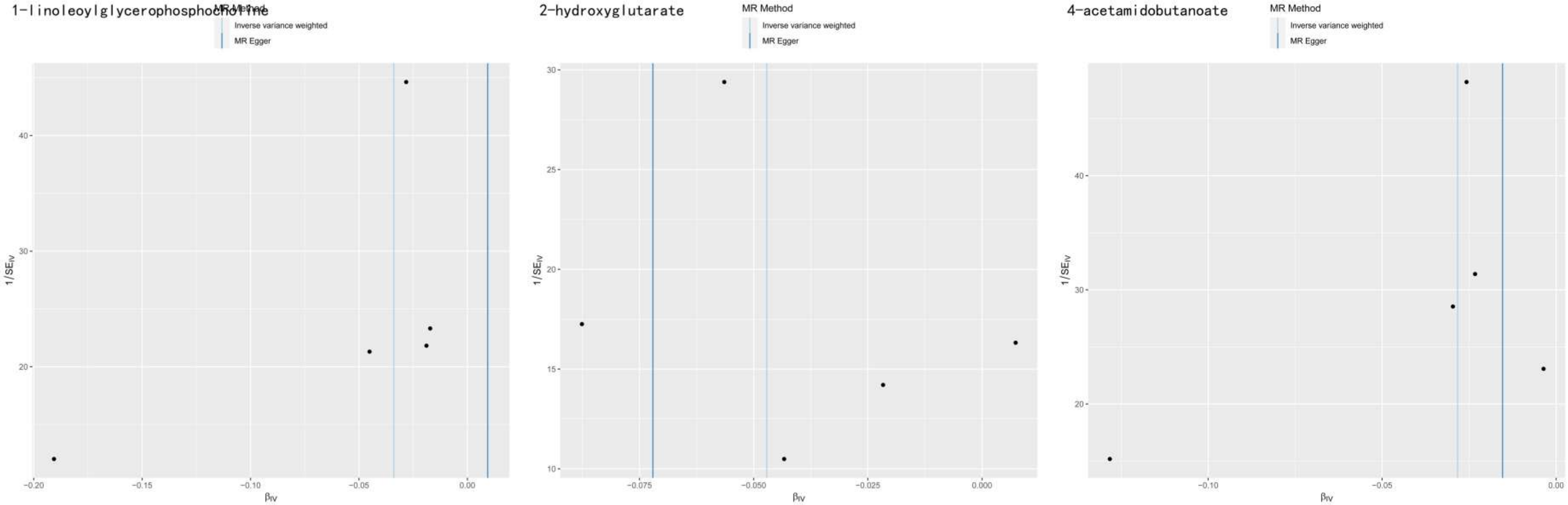
1-linoleoylglycerophosphocholine
MR Method
Inverse variance weighted
MR Egger
1/SE$_{IV}$
β_{IV}
40
30
20
−0.20
−0.15
−0.10
−0.05
0.00
2-hydroxyglutarate
MR Method
Inverse variance weighted
MR Egger
1/SE$_{IV}$
β_{IV}
30
25
20
15
10
−0.075
−0.050
−0.025
0.000
4-acetamidobutanoate
MR Method
Inverse variance weighted
MR Egger
1/SE$_{IV}$
β_{IV}
40
30
20
−0.10
−0.05
0.00

B.

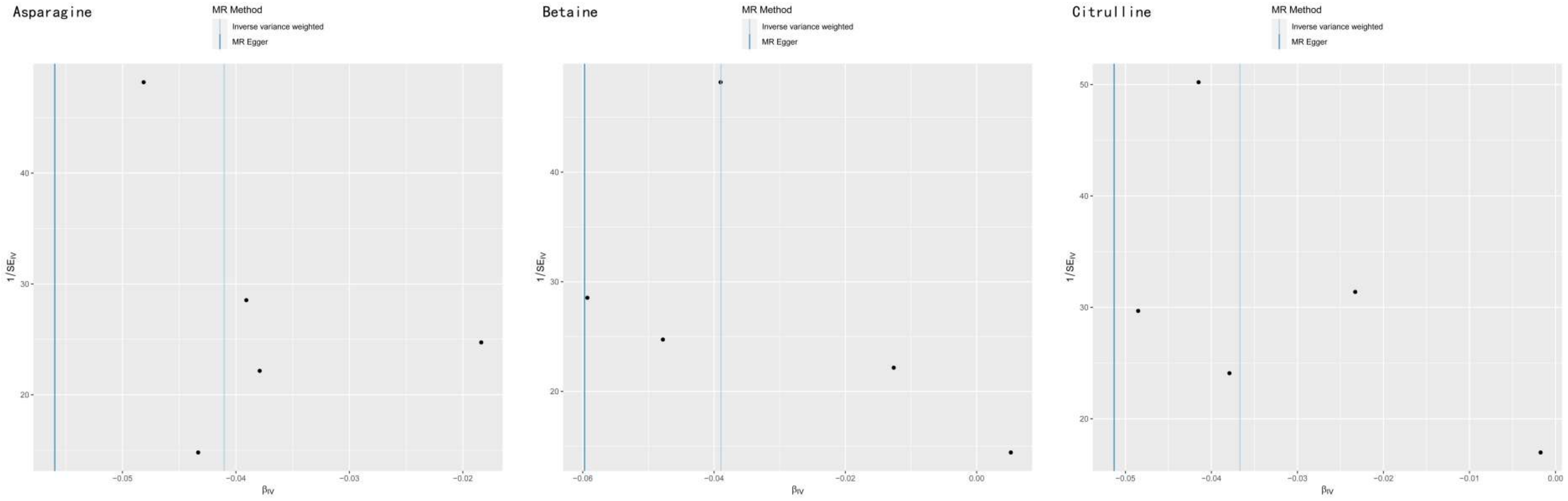
Asparagine
MR Method
Inverse variance weighted
MR Egger
1/SE_IV
β_IV
Betaine
MR Method
Inverse variance weighted
MR Egger
1/SE_IV
β_IV
Citrulline
MR Method
Inverse variance weighted
MR Egger
1/SE_IV
β_IV

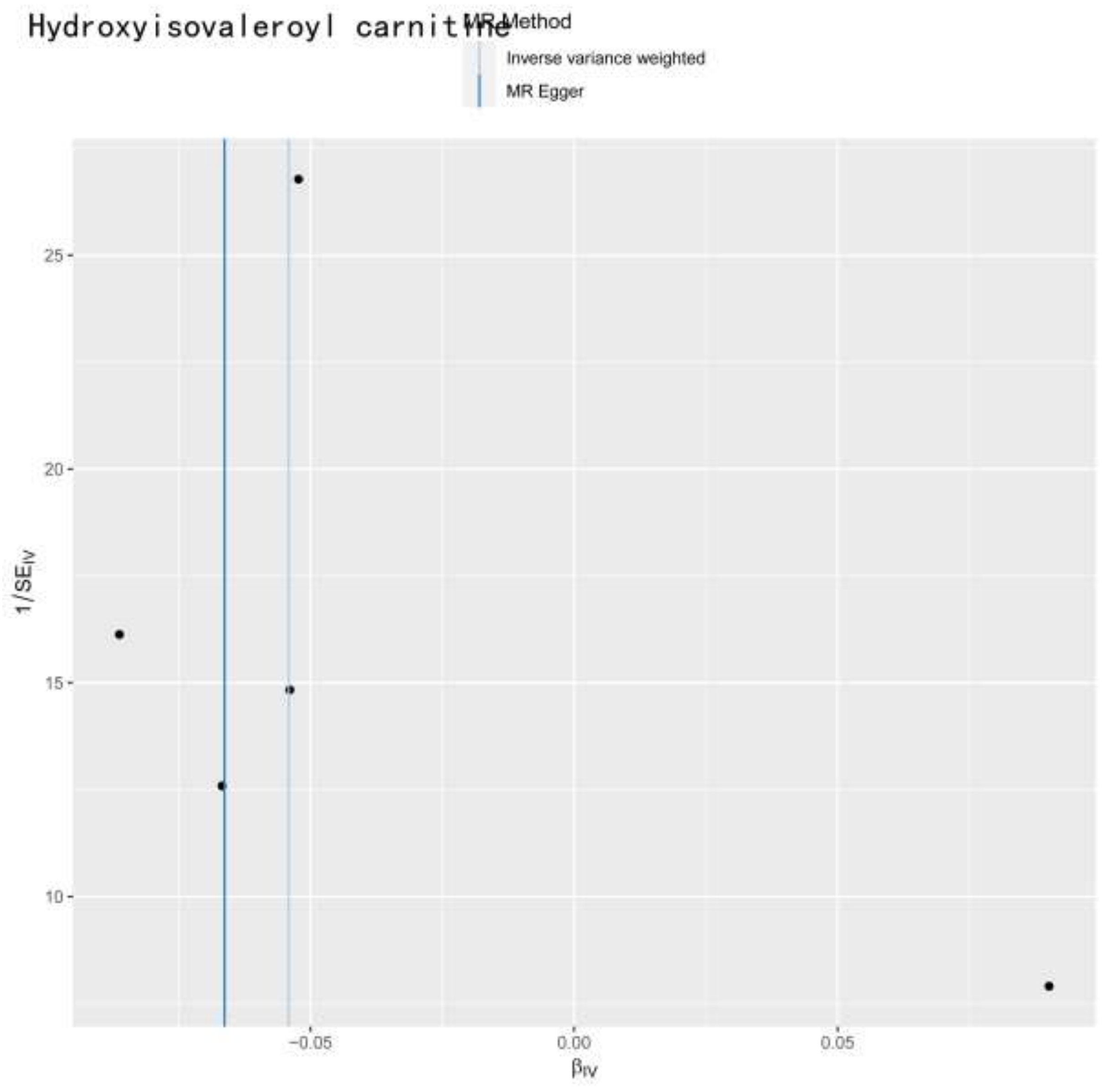

Hydroxyisovaleroyl carnitine
MR Method
Inverse variance weighted
MR Egger
1/SE_IV
β_IV

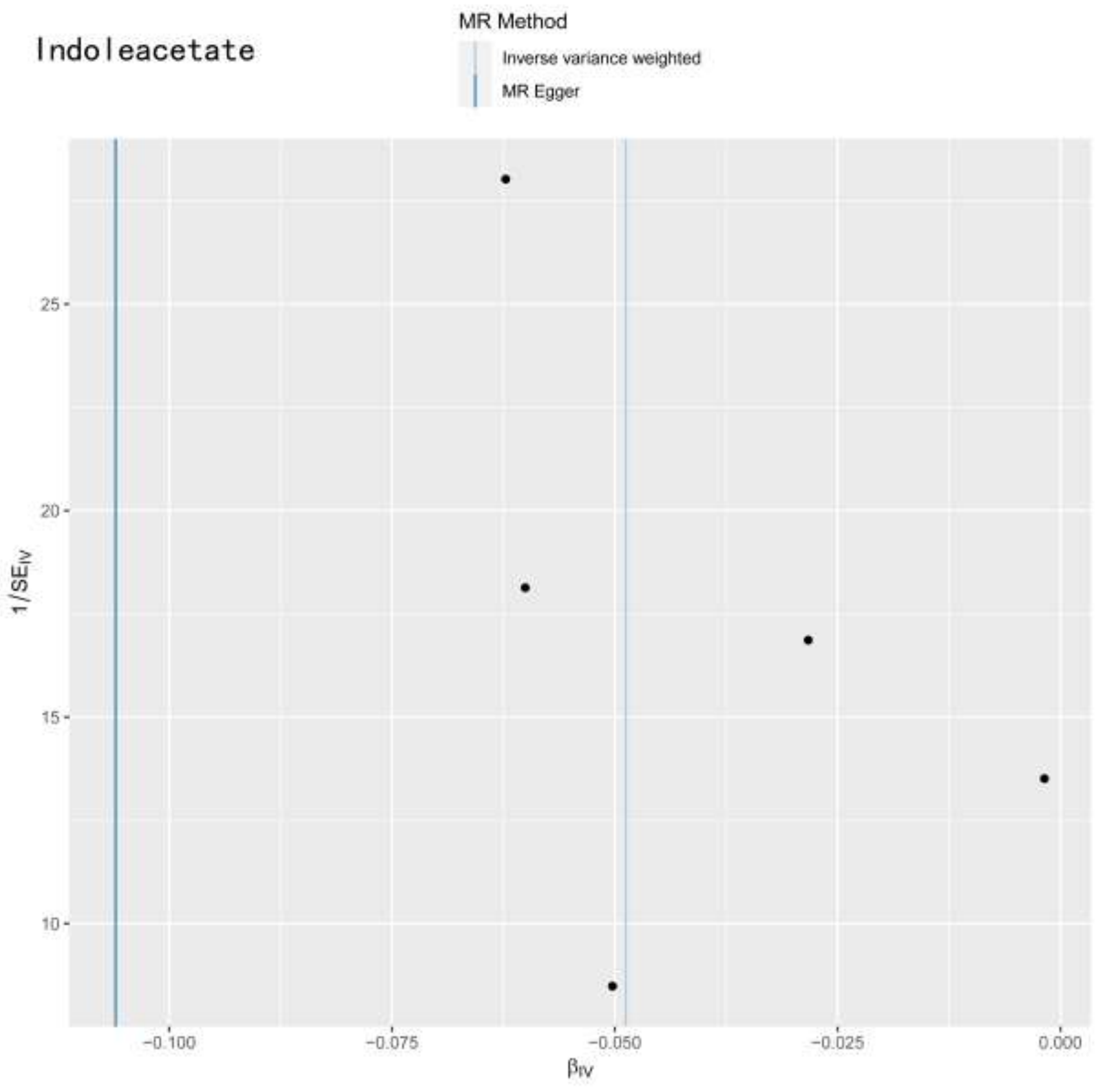

Indoleacetate
MR Method
Inverse variance weighted
MR Egger
1/SE_IV
β_IV

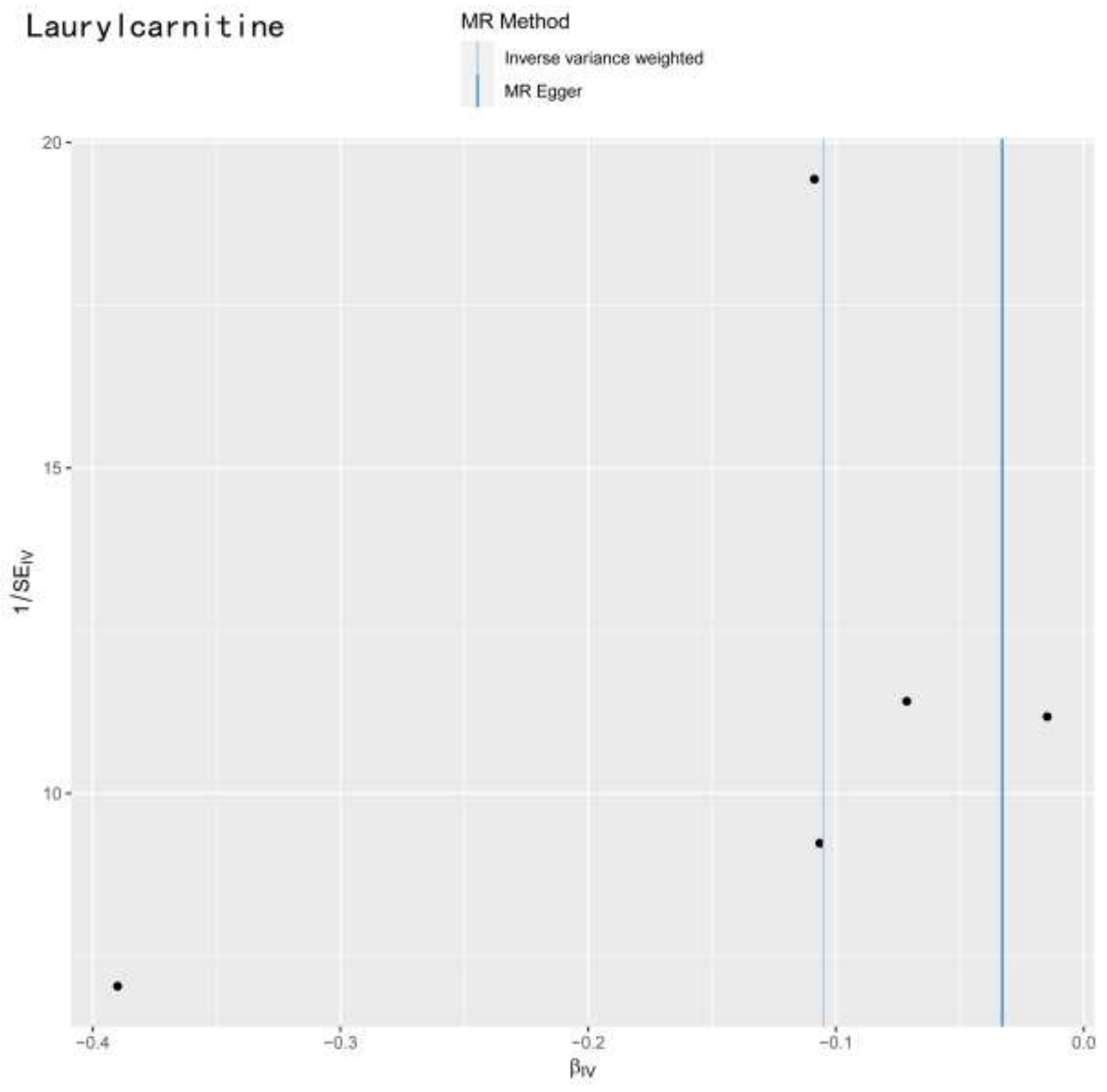

Laurylcarnitine
MR Method
Inverse variance weighted
MR Egger
1/SE_IV
β_IV

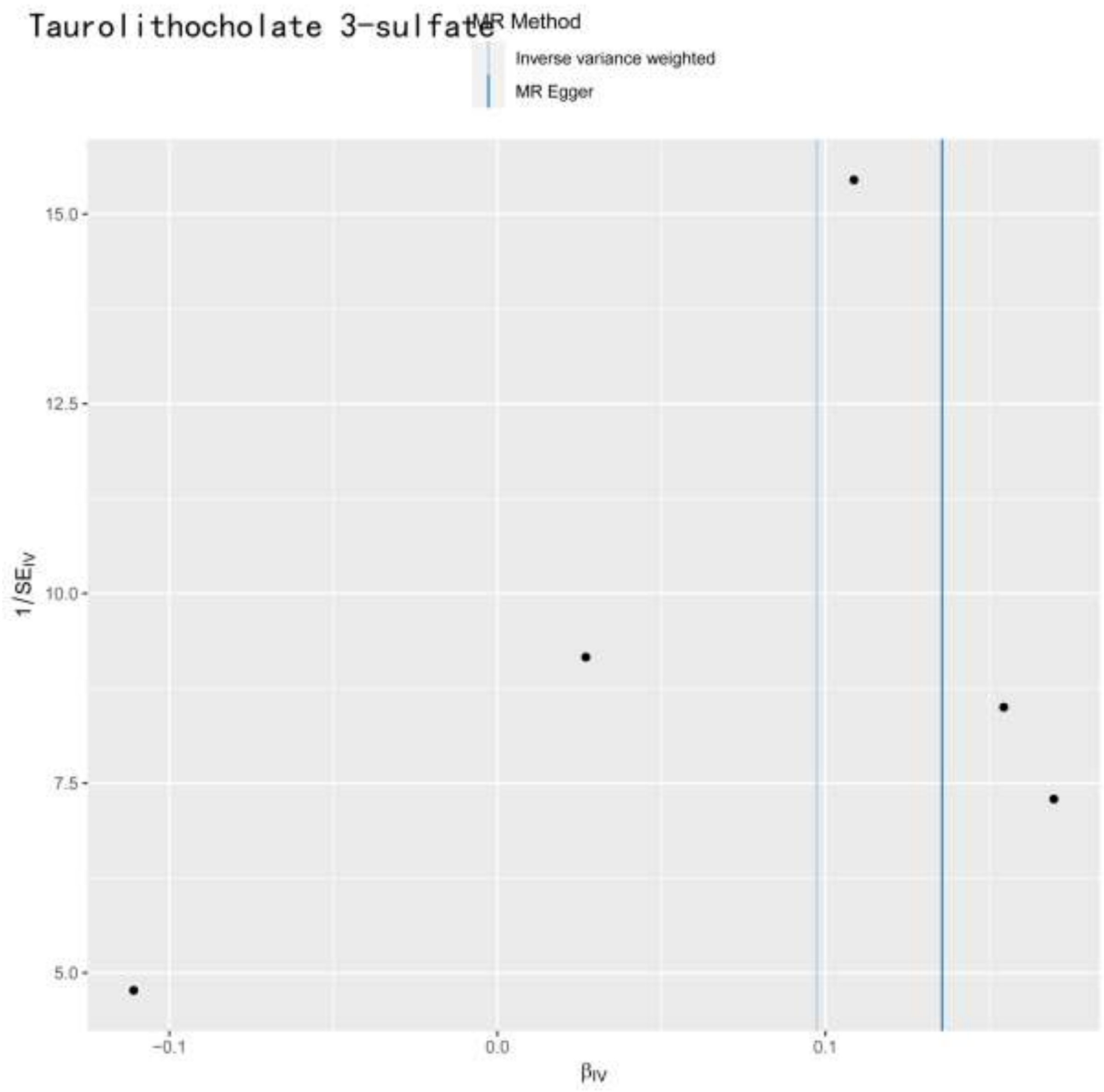

Taurolithocholate 3-sulfate
MR Method
Inverse variance weighted
MR Egger
15.0
12.5
10.0
7.5
5.0
1/SEIV
−0.1
0.0
0.1
βIV

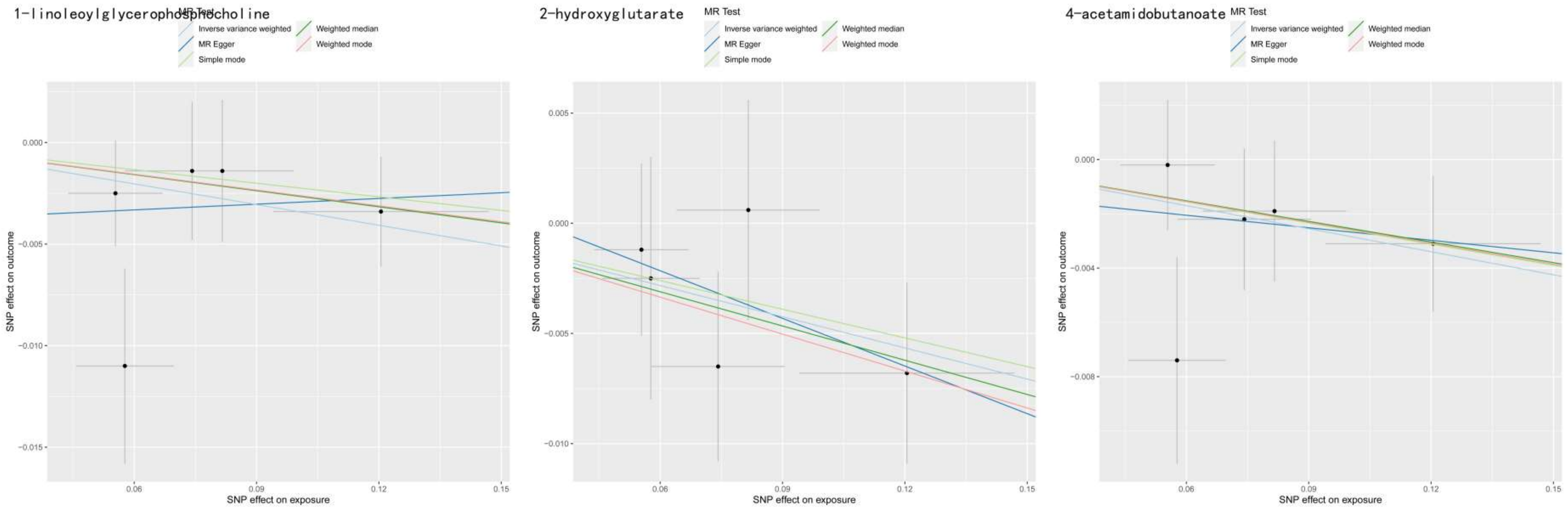

1-linoleoylglycerophosphocholine
2-hydroxyglutarate
4-acetamidobutanoate
MR Test
Inverse variance weighted
MR Egger
Simple mode
Weighted median
Weighted mode
SNP effect on outcome
SNP effect on exposure

c.

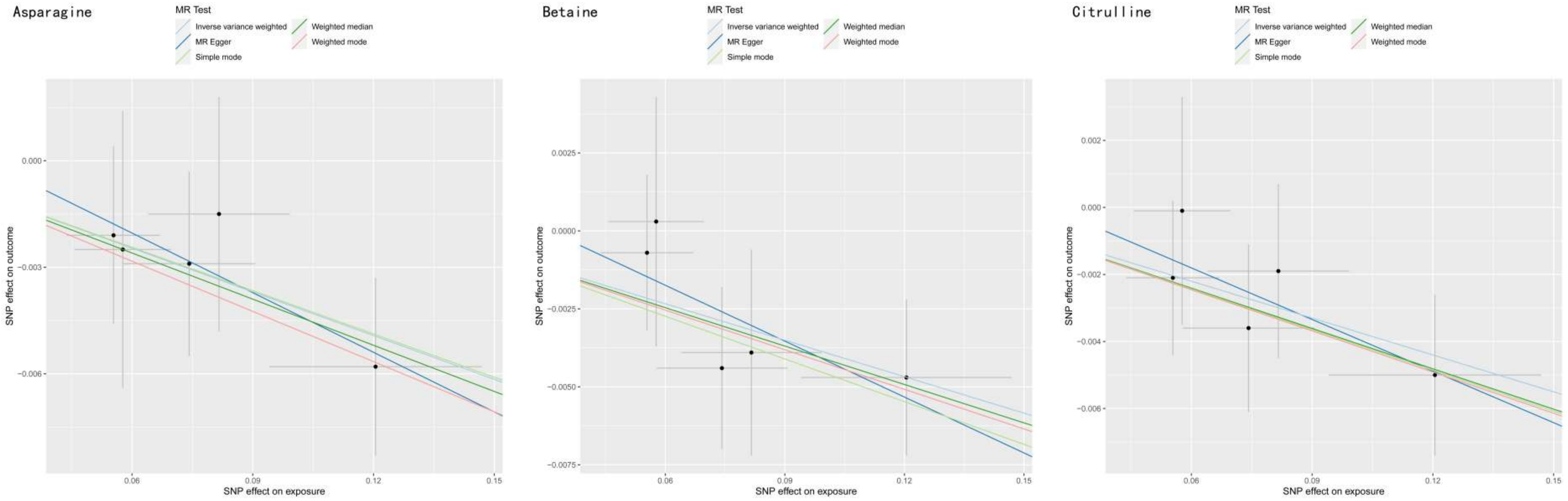

Asparagine
MR Test
Inverse variance weighted
MR Egger
Simple mode
Weighted median
Weighted mode
0.000
-0.003
-0.006
SNP effect on outcome
0.06
0.09
0.12
0.15
SNP effect on exposure
Betaine
MR Test
Inverse variance weighted
MR Egger
Simple mode
Weighted median
Weighted mode
0.0025
0.0000
-0.0025
-0.0050
-0.0075
SNP effect on outcome
0.06
0.09
0.12
0.15
SNP effect on exposure
Citrulline
MR Test
Inverse variance weighted
MR Egger
Simple mode
Weighted median
Weighted mode
0.002
0.000
-0.002
-0.004
-0.006
SNP effect on outcome
0.06
0.09
0.12
0.15
SNP effect on exposure

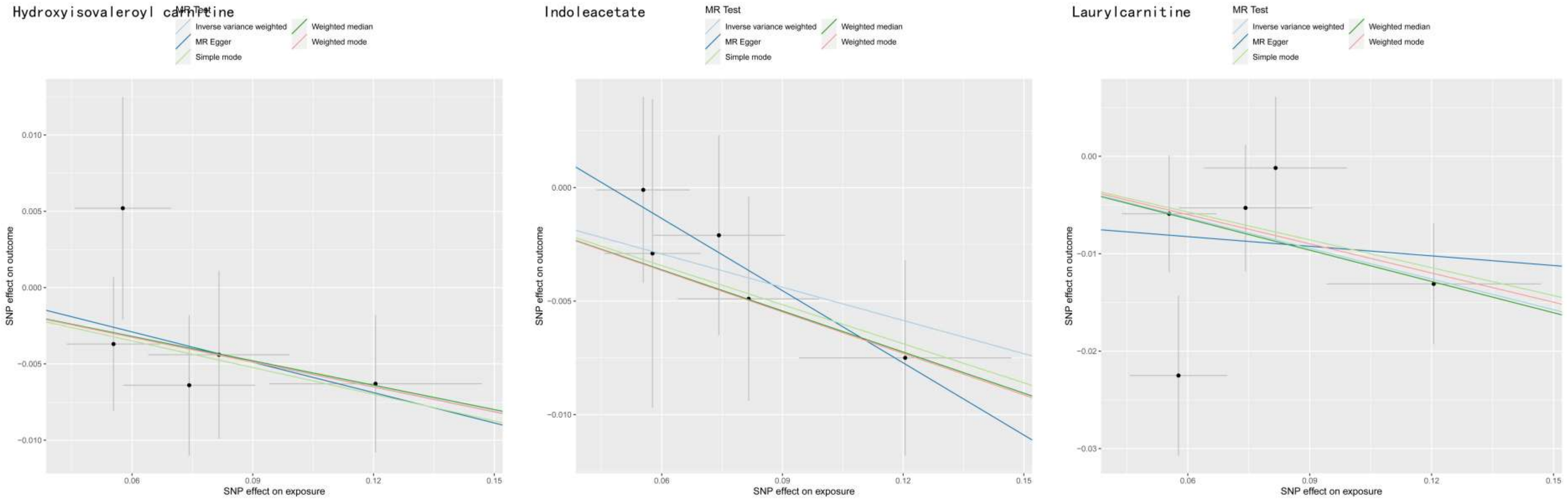

Hydroxyisovaleroyl carnitine
Indoleacetate
Laurylcarnitine
MR Test
Inverse variance weighted
MR Egger
Simple mode
Weighted median
Weighted mode
SNP effect on outcome
SNP effect on exposure
0.06
0.09
0.12
0.15
0.010
0.005
0.000
−0.005
−0.010
0.00
−0.01
−0.02
−0.03

Taurolithocholate 3-sulfate

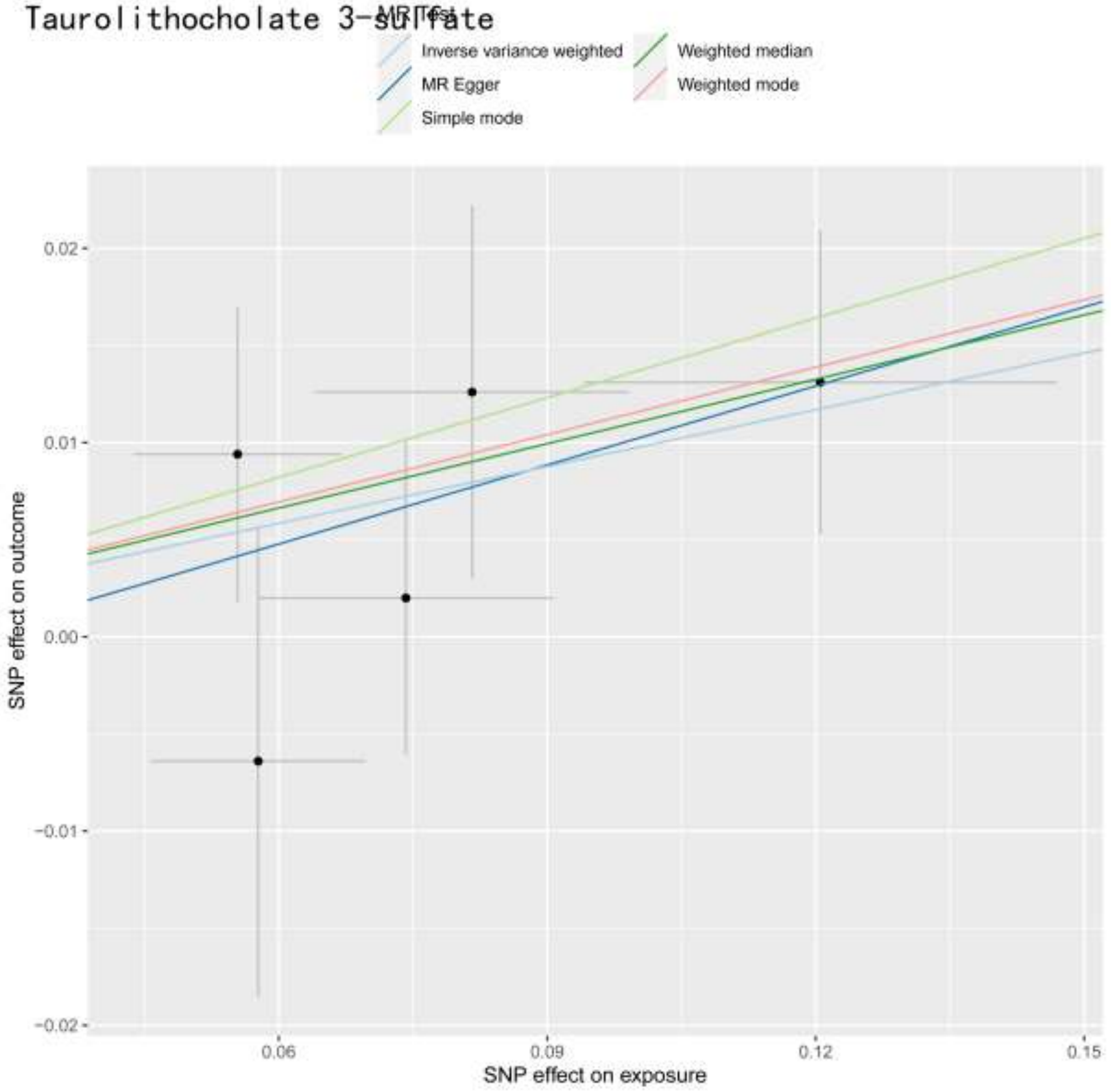

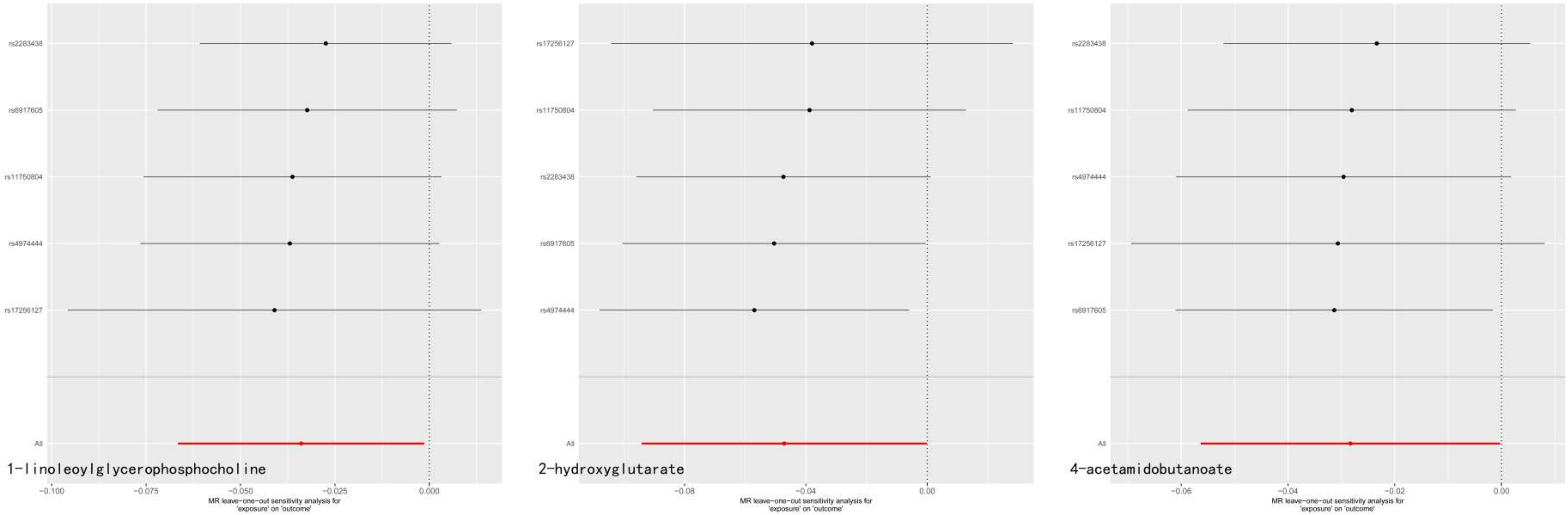
rs2283438
rs6917605
rs11750804
rs4974444
rs17256127
All
1-linoleoylglycerophosphocholine
−0.100
−0.075
−0.050
−0.025
0.000
MR leave−one−out sensitivity analysis for 'exposure' on 'outcome'
rs17256127
rs11750804
rs2283438
rs6917605
rs4974444
All
2-hydroxyglutarate
−0.08
−0.04
0.00
MR leave−one−out sensitivity analysis for 'exposure' on 'outcome'
rs2283438
rs11750804
rs4974444
rs17256127
rs6917605
All
4-acetamidobutanoate
−0.06
−0.04
−0.02
0.00
MR leave−one−out sensitivity analysis for 'exposure' on 'outcome'

D.

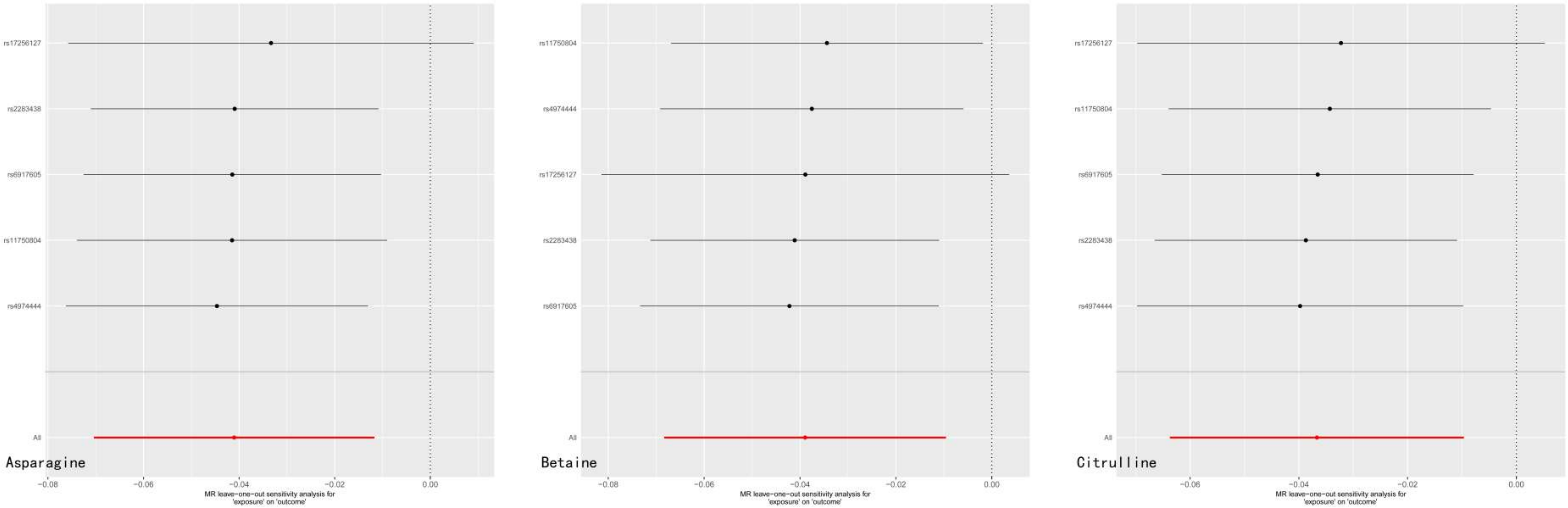
Asparagine
rs17256127
rs2283438
rs6917605
rs11750804
rs4974444
All
−0.08
−0.06
−0.04
−0.02
0.00
MR leave−one−out sensitivity analysis for
'exposure' on 'outcome'
Betaine
rs11750804
rs4974444
rs17256127
rs2283438
rs6917605
All
Citrulline
rs17256127
rs11750804
rs6917605
rs2283438
rs4974444
All

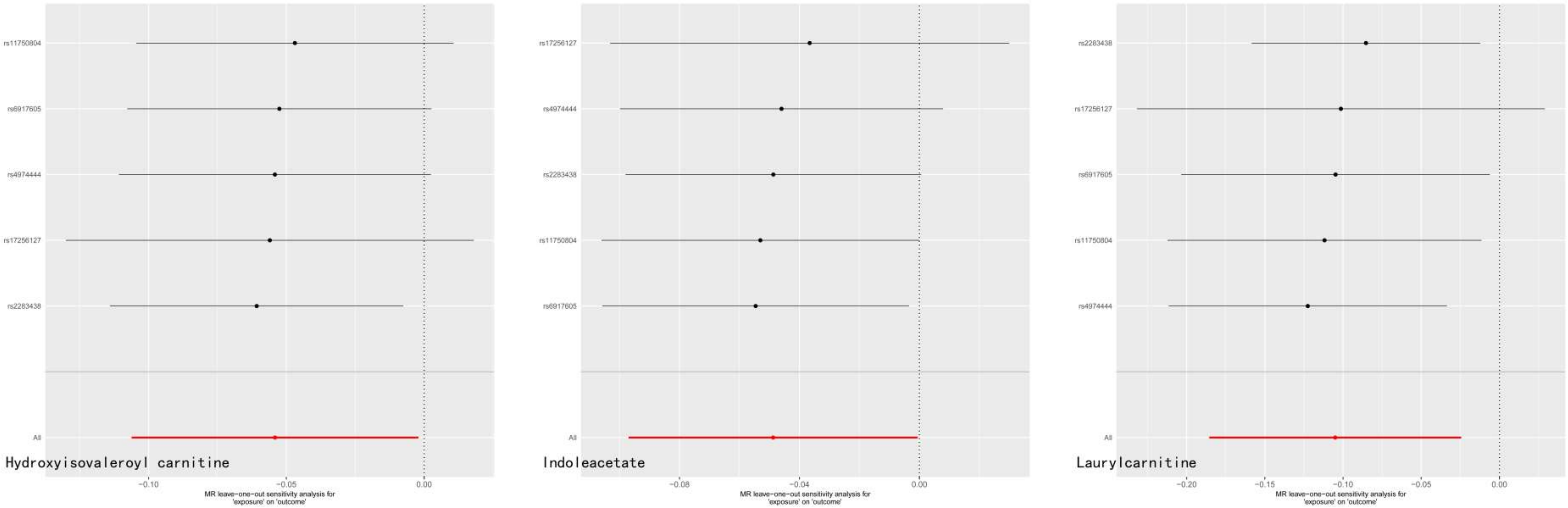

rs11750804
rs6917605
rs4974444
rs17256127
rs2283438
All
Hydroxyisovaleroyl carnitine
−0.10
−0.05
0.00
MR leave−one−out sensitivity analysis for
'exposure' on 'outcome'
rs17256127
rs4974444
rs2283438
rs11750804
rs6917605
All
Indoleacetate
−0.08
−0.04
0.00
MR leave−one−out sensitivity analysis for
'exposure' on 'outcome'
rs2283438
rs17256127
rs6917605
rs11750804
rs4974444
All
Laurylcarnitine
−0.20
−0.15
−0.10
−0.05
0.00
MR leave−one−out sensitivity analysis for
'exposure' on 'outcome'

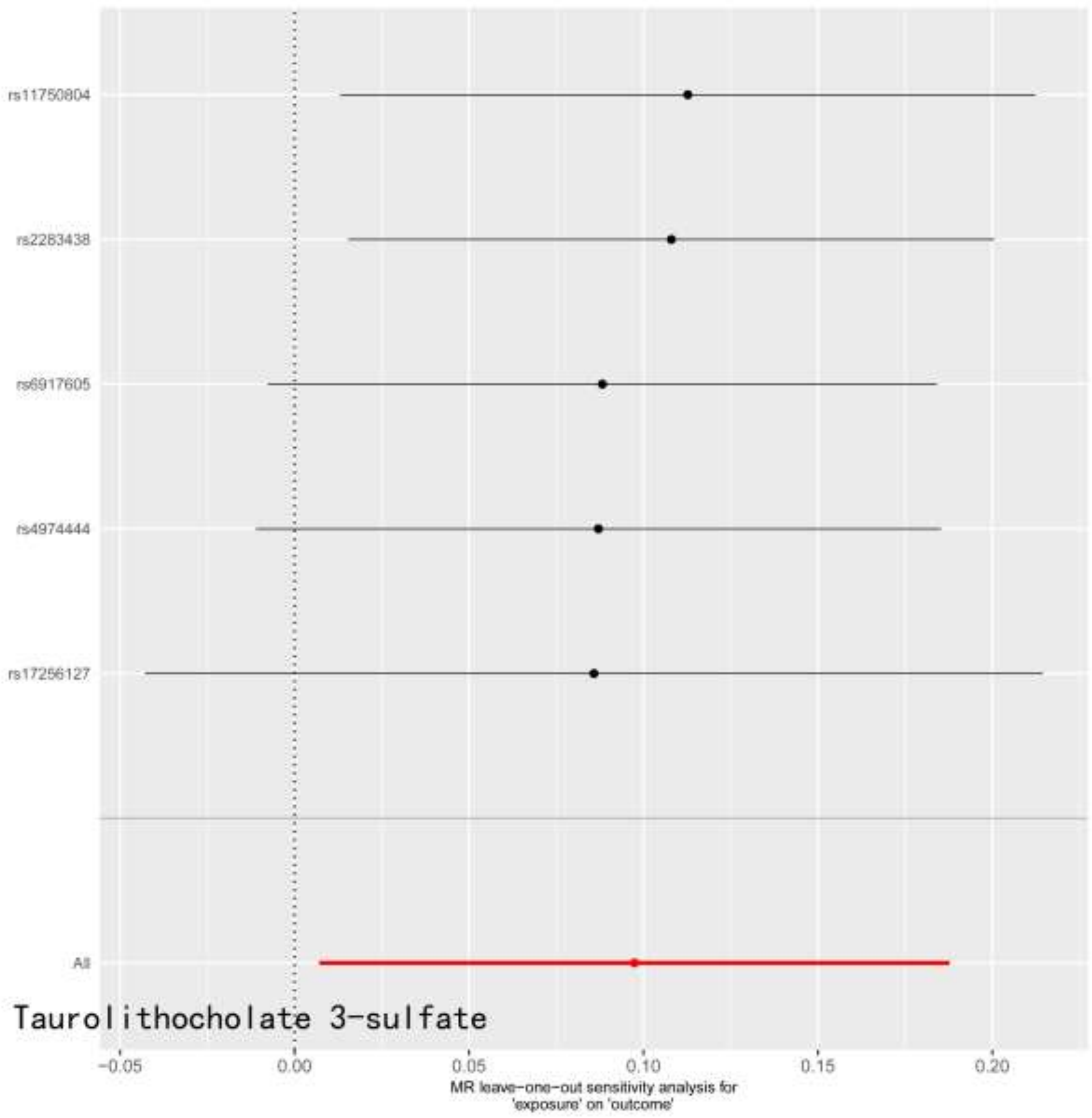
rs11750804
rs2283438
rs6917605
rs4974444
rs17256127
All
Taurolithocholate 3-sulfate
−0.05
0.00
0.05
0.10
0.15
0.20
MR leave−one−out sensitivity analysis for
'exposure' on 'outcome'
